# On an electromagnetic calculation of ionospheric conductance that seems to override the field line integrated conductivity

**DOI:** 10.1038/s41598-024-58512-x

**Published:** 2024-04-02

**Authors:** Russell B. Cosgrove

**Affiliations:** Center for Geospace Studies, SRI International, Menlo Park, CA USA

**Keywords:** Astrophysical plasmas, Aurora, Magnetospheric physics, Characterization and analytical techniques

## Abstract

The ionospheric conductance is the major quantity that determines the interaction of the magnetosphere with the ionosphere, where the magnetosphere is the large region of space affected by Earth’s geomagnetic field, and the ionosphere is its electrically conducting inner boundary, lying right on the edge of the atmosphere. Storms and substorms in space dissipate their energy through ionospheric currents, which heat the atmosphere and disrupt satellite orbits. The ionospheric conductance has, heretofore, been estimated using the staticized physics known as electrostatic theory, which finds that it can be computed by integrating the zero-frequency conductivity along the lines of Earth’s geomagnetic field. In this work we test this supposition by deriving an electromagnetic solution for collisional plasma, and applying it to obtain a first-ever fully-electromagnetic calculation of ionospheric conductance. We compare the results to the field line integrated conductivity, and find significant differences on all scales investigated. We find short-wavelength, mode-mixing, and wave-admittance effects that were completely unexpected. When this theoretical result is matched with recent observational findings for the scale of the magnetosphere-ionosphere coupling-interaction, there results a situation where small- to intermediate-scale effects really may contribute to global modeling of the Sun-Earth system.

## Introduction

Understanding the sun-earth interaction requires linking together the physics of a number of disparate regimes of earth and space, and this process can sometimes reveal inconsistencies in modeling and lead to learning opportunities. One of these opportunities comes in the coupling of the magnetosphere and ionosphere, where the magnetosphere is the large region of space affected by Earth’s geomagnetic field, and the ionosphere is its electrically conducting inner boundary, lying right on the edge of the atmosphere. From the perspective of global simulations the ionosphere serves as the electrical load that bounds the simulation space for the magnetosphere, dissipating the electrical energy from storms and substorms though currents that heat the atmosphere and accelerate its flow. These effects couple through the larger system, with one notable outcome being the disruption of satellite orbits^1.^ And similar effects are expected on other planets^2^.

The learning opportunity arises because the magnetosphere is modeled with electromagnetic physics, whereas the ionosphere is modeled with the staticized physics known as electrostatic theory, due to difficulty handling the ion-neutral collisions that create the electrical conductivity. While there exist strategies for coupling the regions, they involve a binary switch from electromagnetic physics to electrostatic physics that occurs at some chosen altitude. It would certainly be more natural to continue the full electromagnetic physics through the ionosphere, but failing this, it should at least be demonstrated that the electrostatic physics can be derived from the electromagnetic physics. For example, we should be able to find the electromagnetic modes that propagate in the ionosphere and calculate their properties like wavelength and characteristic admittance, and show that these are consistent with electrostatic theory. This validation was attempted in 2016^3^, but the results were not favorable.

In this paper we seek to respond to this challenge by generalizing the transmission line methods of electrical engineering, and using them to explain how a gradual transition can be accomplished from the electromagnetic regime to the electrostatic regime, as applicable for a collisional plasma. We apply this understanding to developing a new electromagnetic calculation for the collisional ionosphere. And we find that the traditional electrostatic paradigm for ionospheric science is, in fact, not applicable for transverse scales above about 100 m. However, the new calculation can serve as a replacement for a wide variety of space weather problems, from equatorial spread-F to traveling-ionospheric-disturbances and auroral phenomena. Thus the goals of this study are actually much broader than magnetosphere-ionosphere coupling, and we have chosen to emphasize the latter partly because it involves a well-known physical quantity that is amenable to calculation, and which is sensitive to the assumptions of electrostatic theory.

In regard to the term “electrostatic paradigm for ionospheric science,” we will compare the electromagnetic theory to both the “electrostatic-wave theory,” where the Poisson equation is substituted for the Maxwell equations, as well as to the much more simplified “electrostatic theory,” where all time derivatives are set to zero and what remains of the equations of motion are applied to a boundary value problem^4–6^. We will use these terms, “electrostatic-wave theory” and “electrostatic theory,” to make this important distinction. The former (electrostatic-wave theory) is used predominantly on scales below 100 m, such as for calculating the incoherent scatter radar spectrum, where we do not find any reason to question it. But the latter (electrostatic theory) is used over the larger scales that are relevant to magnetosphere-ionosphere coupling, and which also include a large variety of mid- and low-latitude phenomena where the mapping of electric field along geomagnetic field lines is usually assumed. In all of these cases we find that the ionosphere should be much more interesting than previously expected. Wave phenomena are predicted that are alien to electrostatic theory, and which greatly affect the mapping of electric field, which is also a crucial assumption in the electrostatic calculation of ionospheric conductance.

The new calculation originates from a 2016 study^3^ of a fully-electromagnetic but homogeneous ionosphere on smaller spatial scales, and is described in detail in a 50 page preprint that is included here as Supplementary Information. For convenience I will sometimes refer to the first of these as Cosgrove-2016^3^. The main paper provides a condensation of the salient features of the new calculation, with the Supplementary Information serving as a detailed manual.

### Focus on ionospheric conductance

Although the transmission line method includes calculation of the local field quantities like electric and magnetic field, discussion will be focused on the “ionospheric conductance”^7,8^, which is a linearized and non-local quantity the non-locality of which is normally handled with electrostatic theory, through the electric field mapping assumption. As such, the ionospheric conductance provides an appropriate differentiator for testing electrostatic theory, such as the scale-size-dependent electric-field mapping it entails. In addition, MHD and other models of the magnetosphere generally employ an inner boundary condition that is established by considering the ionosphere as an electrical load, characterized by this ionospheric conductance through a current continuity equation^9–15^,

1 $$\begin{aligned} j_{\parallel }= & {} \vec {\nabla }_{\perp }\cdot \left( \overleftrightarrow {\Sigma }\vec {E}_{\perp }\right) \;\text {inhomogeneous case}\nonumber \\= & {} \Sigma \;\vec {\nabla }_{\perp }\!\cdot \vec {E}_{\perp }\;\;\text {vertically-inhomogeneous case (horizontally uniform),} \end{aligned}$$

where $j_{\parallel }$ is the current density into the ionosphere, $\vec {E}_{\perp }$ is the perpendicular electric field, and $\overleftrightarrow {\Sigma }$ is the ionospheric admittance tensor, which is usually assumed to be real and thus called the “ionospheric conductance tensor,” and which becomes the scalar “ionospheric conductance,” $\Sigma$, in the horizontally uniform case that we consider here. Thus, from the perspective of circuit theory, the ionospheric conductance is meant to represent the input admittance seen from above the ionosphere, and this input admittance is an important quantity for global modeling of the Sun-Earth interaction. Under electrostatic theory it is derived by integrating the (zero frequency) Pedersen conductivity along the geomagnetic field, based on the mapping assumption. Dropping the assumption that it is purely real, and dropping the assumption that it can be derived from electrostatic theory, we arrive at an important problem that is well suited to transmission line theory: that of deriving the ionospheric input admittance, $\Sigma$.

### Lack of previous research

I am aware of only one reference^16^, and possibly its predecessor^17^ should be included, which at first appears to have comparable results, and these actually do appear to support the estimation of conductance as the field line integrated conductivity. However, it turns out that these calculations actually rely on electrostatic theory in certain key areas. In fact, the identification of these boundary-value-problem type solutions as being electrostatic was one of the major results of Cosgrove-2016^3^, and this is what clarified the need for the current study. The equations of motion are named as such because they describe time evolution; they exist in the context of a causal structure, which determines what data constitute a complete set of initial conditions. Initial conditions are data on a spacelike surface, and a boundary is not a spacelike surface and so cannot be reliably used. While it can sometimes be useful to solve boundary value problems, which is electrostatics, if there is ever a conflict it is always the solution to the time evolution problem that should be taken as the physical prediction of the equations^3,18,19^. So that is what we provide in this paper.

There are also a number of time domain simulations that might seem applicable^20–27^. But I have not been able to find where any actually compute the ionospheric conductance, at least not as a function of the transverse wavelength, which is necessary for the results to be of interest. I have discussed some likely reasons in the Background Section 2 of the Supplementary Information, although with humility as I am not in a position to properly critique the full collection of complex and sophisticated modeling efforts. The summary is that these time-domain simulations are addressed primarily to complex transient phenomena on global scales, and are not optimized for calculating the wavelength-dependent conductance, which is only accessible to them through potentially impractical boundary conditions, dramatically increasing the vertical resolution, and then running the simulation for a long time, until everything stops changing (steady state, which may require linearization). And even so, there is some question of whether the approximations used to remove the radio-frequency modes might compromise the unexpected collisional effects found below, which greatly modify the contribution from lower E-region conductivity.

### Small-scale effects and global modeling

An additional motivation for this work comes from recent observational findings that the magnetosphere-ionosphere interaction may actually occur over scales significantly shorter than previously expected. For example, using data from the Swarm satellites, it has been found^28^ that half the Poynting flux may be associated with events having transverse-scale-size less than 250 km. If this is true then the results presented here take on an additional level of importance, because they mean that the varied-effects found at the lower-end of the scale-size regime studied below, may in fact be very important for global modeling; it is not only the upper-end that matters. This may be a situation where small- to intermediate-scale effects really do contribute to global modeling.

## Results

### Gedankenexperiment and criteria for electrostatic theory

**Figure 1:**
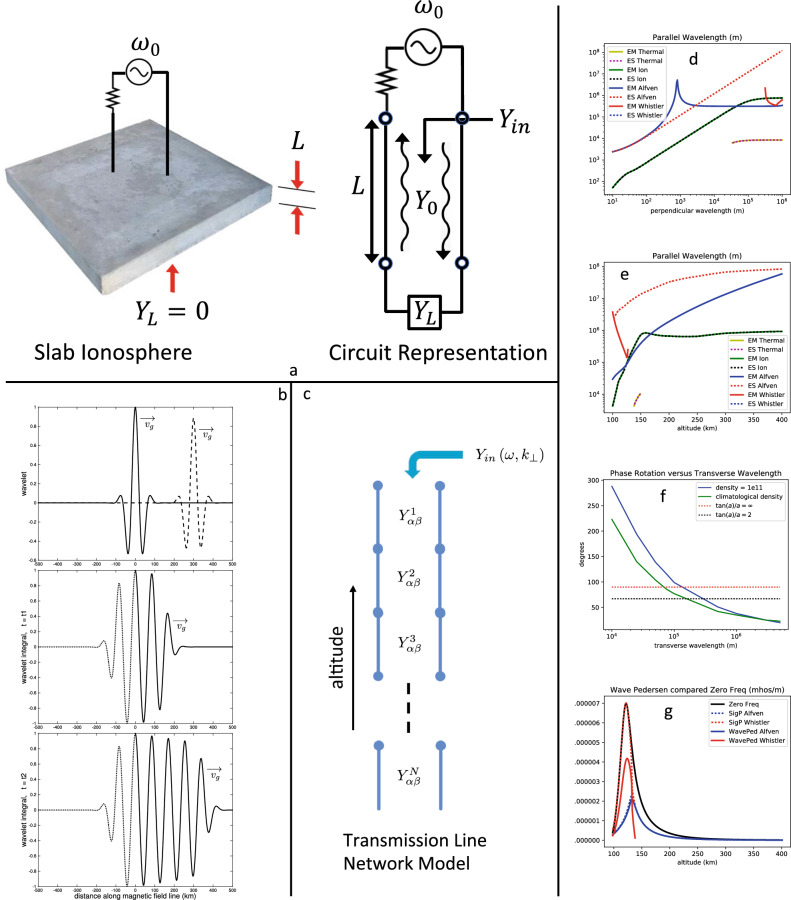
Panels (**a**–**c**): Development of the model calculation with (**a**) Gedankenexperiment; (**b**) wave packet interpretation; and (**c**) cascaded transmission line model. Panels (**d**) and (**e**): Parallel-wavelength including for electrostatic-wave theory ($n_{e}=10^{11}\,\text {m}^{-3}$ for 145 km altitude [panel (**d**)], and 100 km wavelength [panel (**e**)]). Panel (**f**): Phase rotation through the ionosphere as a function of transverse wavelength, with benchmarks demarcated by dashed lines. Panel (**g**): Wave-Pedersen conductivity compared to the full and zero-frequency Pedersen conductivities, for the Alfvén and Whistler waves ($\lambda _{\perp }=1000\,\text {km,}\,n_{e}=4.7\times 10^{9}\,\text {m}^{-3}$).

To get us started, consider a sort of simplest-case gedankenexperiment where the ionosphere supports only one mode of propagation (wave mode), and consists of a uniform slab of plasma of thickness *L*, as shown in Fig. 1a. The left hand side of the figure shows the uniform slab of plasma, with a harmonic source above that we can think of as the magnetosphere, and an open circuit boundary condition at the bottom of the conducting region. The right hand side of the figure shows the electrical schematic applicable to this case where, because only one wave mode is supported, a standard transmission line^29^ can be used. The length of the transmission line (*L*) is the thickness of the ionosphere, and its characteristic admittance $(Y_{0})$ describes the wave mode for the plasma. Usually $Y_{0}$ is not a function of frequency, but for the ionosphere $Y_{0}$ is a function of the frequency and wavelength.

In transmission line theory, the usual formula for the input admittance $(Y_{in})$ is found by the complex-plane superposition of two oppositely propagating waves, such that the load admittance $Y_{L}$ is realized at the end of the transmission line. This boundary condition determines the waves up to a single complex constant, which can be set to match the amplitude and phase of the source, if it is desired to know the fields inside the transmission line. The derivation of the equation for $Y_{in}$ is given in many textbooks, and I give here equation 3.88 from Section 3.5 of the classical textbook by Collin^29^,

2 $$\begin{aligned} Y_{in}=Y_{0}\frac{iY_{0}\tan \phi +Y_{L}}{iY_{L}\tan \phi +Y_{0}}, \end{aligned}$$

where the “electrical length” $\phi _{r}=2\pi L/\lambda _{z}$ is the phase rotation along the transmission line, and in this case $\phi =\phi _{r}+i\phi _{i}$ also has an imaginary part, $\phi _{i}=-L/l_{dz}$, which uses a dissipation scale length $(l_{dz})$ to characterize the attenuation of the wave due to dissipation. Equation (2) is the basis for the classical “Smith chart” from electrical engineering, which provides a graphical representation that was a staple of microwave laboratories in the days before computers were widely available^29^.

To treat the ionosphere we set $Y_{L}=0$, and then compare the results to electrostatic theory, where as described in the Introduction, $Y_{in}$ is to be compared to the field line integrated Pedersen conductivity $(\Sigma _{P})$,

3 $$\begin{aligned} \left. Y_{in}\right| _{Y_{L}=0}= & {} iY_{0}\tan k_{z}L\nonumber \\ \Sigma _{P}= & {} \sigma _{P}L, \end{aligned}$$

where $\sigma _{P}$ is the zero-frequency Pedersen conductivity, and I have used the notation $k_{z}=2\pi /\lambda _{z}-i/l_{dz}$. The equation for $\Sigma _{P}$ assumes that electrostatic theory predicts that the electric field will map unchanged through the ionosphere. This “mapping condition” has been found to hold for transverse scales greater than about 10 km^5,6,30^. Equating the right-hand-sides of the two equations in (3), and requiring that the match hold over a range of *L*, gives the criteria for electrostatic theory under the mapping condition,

4 $$\begin{aligned} \lambda _{z}\gg & {} L,\nonumber \\ l_{dz}\gg & {} L,\quad \text {and}\nonumber \\ iY_{0}k_{z}= & {} \sigma _{P}. \end{aligned}$$

**Figure 2:**
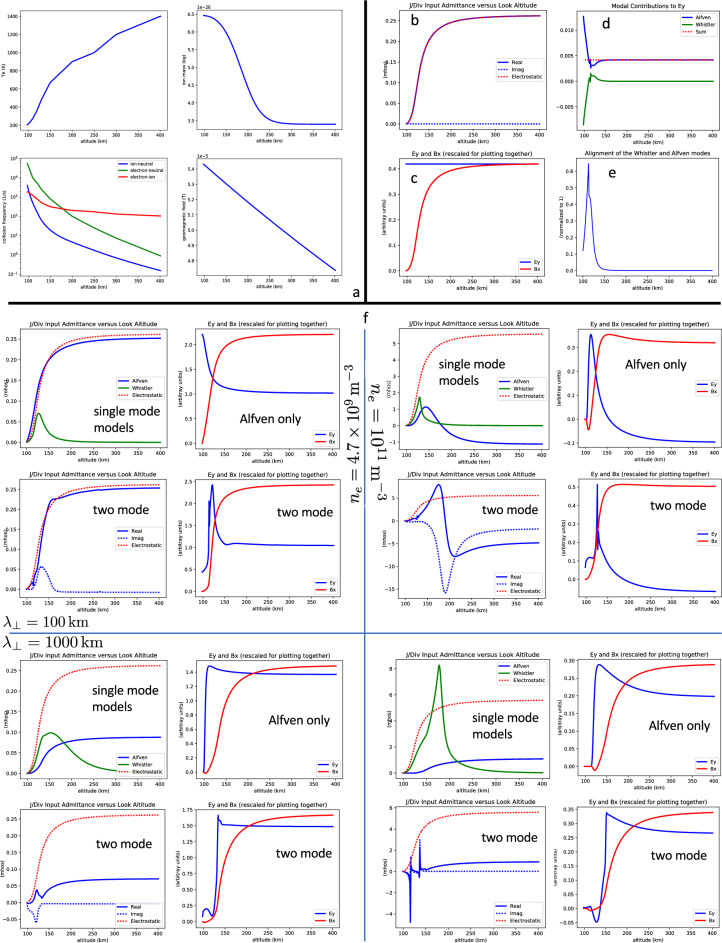
Panel (**a**): Background ionospheric parameters used for calculations, where $\nu _{ei}$ is shown for the case $n_{e}=10^{11}\,\text {m}^{-3}$. Panels (**b**) and (**c**): Validation results showing reproduction of electrostatic theory for conductance (Panel (**b**)); and for electric-field and parallel-current (Panel (**c**)). Panel (**d**): Modal contributions to electric field for the validation. Panel (**e**): Alignment of the Whistler and Alfvén waves, showing the near degeneracy, which causes the kinky features. Panel (f): Actual model results with the 100 km transverse wavelength on top, 1000 km on the bottom, $n_{e}=10^{11}\,\text {m}^{-3}$ on the right, and $n_{e}=4.7\times 10^{9}\,\text {m}^{-3}$ on the left ($n_{e}$ is kept constant with altitude).

The first two criteria are well known in electrical engineering, and are the conditions for the transmission line to be transparent, that is, $Y_{in}\rightarrow Y_{L}$ as $\phi =k_{z}L\rightarrow 0$. The third criterion equates the electrostatic Pedersen conductivity $(\sigma _{P})$ to what has previously^3^ been called the wave-Pedersen conductivity $(iY_{0}k_{z})$. This criterion implies that $Y_{0}k_{z}$ is an imaginary number, which, since this must hold across a range of conditions, suggests a likelihood that either $Y_{0}$ or $k_{z}$ is strongly imaginary, with the other being strongly real. As it turns out, it is the former case that has been found (e.g., see Table 2 of Cosgrove-2016^3^; and Fig. 2, panels (g) and (h) of the Supplementary Information) to be the actual one for the ionosphere, that is, $Y_{0}$ is strongly imaginary, $\lambda _{z}\ll l_{dz}$, and $l_{dz}$ is almost always long enough to be ignored. This means that the tangent function dependence in Eq. (3) can cause some very unexpected resonant effects, such as a conductance that passes through zero.

### Deriving transmission line theory from the general solution

The derivation of transmission line theory from the electromagnetic fluid equations may be found in the Methods Section “Methods”, including additional physical interpretation. Perhaps the key thing to understand is that there is no separate DC or electrostatic component missing from transmission line theory. The solution appears as a sum of waves simply because it is obtained in the Fourier domain, and some waves, such as the Alfvén wave, can operate at zero frequency.

In Section “Methods” we find that two wave-modes need to be included in the transmission lines for the collisional plasma of the ionosphere, which we call the Alfvén and Whistler waves. Two other wave-modes, which we call the Ion and Thermal waves, are sometimes able to propagate, but are highly-lossy and decoupled on the scales we examine in this work.

The wave modes are expressed in terms of the eigenvalues $\omega _{j}$ and eigenvectors $\vec {h}_{j}$ of the matrix $H_{5}$, which contains the linear parts of the electromagnetic fluid equations (without approximations). The eigenvector describes the disturbance of the plasma and determines such quantities as the characteristic admittance $(Y_{0})$ for the *j*th mode. The eigenvalue is the complex frequency whose real part must match the frequency of the source, $\omega _{jr}(\vec {k}=\vec {k}_{0j})=\omega _{0}$, and this determines the vertical wavevector $(k_{0jz})$ as a function of the chosen transverse wavevector ($k_{0jy}$, with the convention $k_{0jx}=0$). The imaginary part of the eigenvalue is the dissipation rate, which enters into the formula for the dissipation scale length, $l_{dj}=\left. -\left( \partial \omega _{jr}/\partial k_{z}\right) /\omega _{ji}\right| _{\vec {k}=\vec {k}_{0j}}$. Although it is not necessary, for simplicity we will take the geomagnetic field to be in the vertical (*z*) direction.

Different $k_{0jy}$ and $\omega _{0}$ are associated with different transmission lines, where each can be understood as describing the response to an incoming multi-modal signal with a particular transverse-wavelength and frequency. To treat a general incoming signal (e.g., localized in the transverse dimension) we can superpose the solutions for many such transmission lines (e.g., to form a wavepacket). However, we will not attempt the superposition in this work. In what follows we will consider transverse-wavelengths from 100 km to 1000 km, and then assume a representative 40 m/s transverse-velocity to calculate the associated frequency (i.e., $40\,\text {m/s}=\omega _{0}/k_{0y}$).

### Extension to a vertically inhomogeneous ionosphere

Following the usual approach from transmission line theory, we extend the model to one for a vertically-inhomogeneous ionosphere by cascading a number of short transmission-line sections as depicted in Fig. 1c, where the characteristic admittances have been replaced with tensors $Y_{\alpha \beta }$ (determined by the eigenvectors), since we will be using two modes. The transmission-line sections are joined by enforcing boundary conditions, where two boundary conditions are required for each included wave-mode, in order to have a unique solution. In the case of two wave-modes, the boundary conditions to be enforced are continuity of the two transverse components of electric field, continuity of the parallel component of magnetic field, and continuity of the parallel current. The first three of these are rigorously required, and the fourth is required in order to allow for the possibility of reproducing electrostatic theory, which closes the field aligned current through the Pedersen current for this horizontally uniform case.

A external boundary condition is assumed at the bottom of the E-region, where each mode is assumed to reflect such that the resultant parallel current is zero. The sensitivity to this condition has been tested for a few examples, and there is found to be very little sensitivity. Taking the cascade deeper would allow for solving for the coupling into the ducted modes that propagate in the ionospheric waveguide^31^, but that should create only a modest perturbation at higher altitudes and so we do not include this coupling.

The cascade is solved by bootstrapping from the last section of line, and the procedure may be understood by referring to Eq. (2), where $Y_{in}$ for a second section attached on top of the first can be derived by taking $Y_{in}$ for the first section as $Y_{L}$ for the second section, and etc. This procedure may be generalized for the case of two or more wave modes, and a detailed description is given in Section 5 of the Supplementary Information.

The result of this calculation allows the ionosphere (as seen looking downward from above) to be regarded as a circuit element that may be driven by any arbitrary source of the same transverse wavelength and frequency. And after application of such a source to the top of the cascade, the excitation may be traced from section to section, down to the bottom of the cascade. This allows for making the altitude-resolved plots of electric and magnetic field that are presented later, where the source is specified by assuming an Alfvén wave incident from the magnetosphere.

Generally, the ionospheric circuit-element may be described as a semi-infinite piece of transmission line that supports two modes of propagation, where we might refer to these as “equivalent modes”, since they encompass the effects of the many internal reflections that happen at each boundary in the cascade. However, under the conditions considered the Whistler mode does not propagate at the top of the ionosphere, and so looking down from the top of the ionosphere there is only one equivalent mode to consider. (See Section “Extending the whistler wave” for some technical details on how the cutoff of the Whistler mode is handled.) In this case the ionospheric circuit element may be regarded as an ordinary, semi-infinite piece of transmission-line characterized by the characteristic-wave-admittance of the (single) equivalent-wave-mode. This characteristic wave admittance is then the ionospheric admittance, $\Sigma$, from Eq. (1), which is commonly referred to as the “ionospheric conductance”.

Note that having only one equivalent mode does not mean that mode coupling is not included in the results. Rather, the very significant coupling between the Whistler and Alfvén modes that happens at lower altitudes is included through its affect on the equivalent mode; the nature of the single equivalent mode is a composite of the effects from wave propagation and reflection at all altitudes, in all the modes that get excited.

Looking downward from lower altitudes where the Whistler mode does propagate, there are in fact two equivalent modes, and so a single scalar input admittance is not sufficient to fully determine the four boundary conditions that are enforced in the calculation. However, in the plots presented below we plot only a single scalar input admittance at all altitudes by assuming an Alfvén wave incident in the section immediately above, regardless of what mode or combination of modes would actually be in that section based on an Alfvén wave incident at the top of the ionosphere.

### Findings using only the eigenvectors and eigenvalues

Before discussing the model validation and results we investigate a few of the findings that follow simply from the eigenvalues $\omega _{j}$ and eigenvectors $\vec {h}_{j}$, which we calculate numerically. First is to evaluate the electrostatic-wave theory, by which is meant replacement of the Maxwell equations by the Poisson equation as explained in the Introduction. Making this replacement causes the matrix $H_{5}$ to be replaced by another matrix that we call $H_{5ES}$, both of which may be found in the Supplementary Information (Appendix A). To compare the physics that follows from these two different sets of equations of motion, we use the 40 m/s drift velocity as described in Section “Deriving transmission line theory from the general solution”, and then either assume a 145 km altitude to plot parallel-wavelength versus transverse-wavelength (Fig. 1d), or assume a 100 km transverse-wavelength to plot parallel-wavelength versus altitude (Fig. 1e). The geophysical parameters are set to the typical high-latitude nighttime values shown in Fig. 2a, with $T_{i}=T_{e}$, and with the electron density held constant at $10^{11}\,\text {m}^{-3}$. Except that the electron density is held constant, these are the same geophysical conditions used in Cosgrove-2016^3^, where they are described further in Appendix B. These same parameters will be used in all the results below, except that the electron density will be separately identified. Note that it is the altitude-dependence of the ion-neutral collision frequency that causes the majority of the altitude-dependence in what follows, and to help make this clear, the electron density will always be kept constant with altitude in these examples.

The plots include all the wave-modes that are able to propagate under the assumed conditions, over the ranges they are able to propagate, and the first finding is that the electrostatic-wave equations support one fewer propagating wave-mode. Both Fig. 1d and 1e show that the parallel-wavelengths are in exact agreement for the Ion and Thermal waves, and Fig. 1d shows that for transverse wavelengths less than about 100 m they are also in agreement for the Alfvén wave. So the missing wave-mode appears to be the Whistler wave, which means that the electrostatic-wave equations do not encompass the multi-modal effects that are found to be very important below. With respect to the Alfvén wave, Fig. 1e shows that the wavelength in the E region is more than an order of magnitude longer for the electrostatic analogue, and so neither will the electrostatic-wave equations encompass the short-wavelength effects that are also found (just below) to be very important, in this case without relying on anything other than numerical calculation of the eigenvalues $\omega _{j}$.

Very short parallel-wavelengths have been found for the Alfvén wave for transverse-wavelengths below 50 km^3^, and here we add an estimation of the phase rotation for longer-wavelength signals traversing the ionosphere, by integrating the inverse of the parallel wavelength over altitude. Fig. 1f summarizes these results (details are in the Supplementary Information, Sect. 6.2) by plotting phase-rotation versus transverse-wavelength for two different electron density profiles (a profile with a constant density of $10^{11}$ $\text {m}{}^{-3}$, and the phenomenological profile for nighttime high-latitude conditions from Appendix B in Cosgrove-2016^3^). The horizontal dashed lines in Fig. 1f identify the effect implied for the tangent function factor in Eq. (3). The phase rotation can be so large as to produce a resonance in Eq. (3) for a 100 km transverse wavelength (i.e., $\tan k_{z}L=\infty$). And to emphasize, this result is derived without using the cascaded transmission line model of Fig. 1c, only the eigenvalues $\omega _{j}$ are used.

The third of the electrostatic criteria (4) can be evaluated by using the eigenvectors to calculate $Y_{0}$. In Cosgrove-2016 (e.g., their Figure 16) the wave-Pedersen conductivity $(iY_{0}k_{z})$ for the Alfvén wave was compared to the usual (i.e., zero-frequency) Pedersen conductivity, and it was found that they are not the same. Here we plot the wave-Pedersen conductivities for both the Alfvén and Whistler waves versus altitude, for a transverse wavelength of 1000 km and density of $4.7\times 10^{9}\,\text {m}^{-3}$. The results are shown in Fig. 1g, which also shows the zero-frequency Pedersen conductivity and the “full Pedersen conductivities,” which are the Pedersen conductivities evaluated with the imaginary part of $\omega _{j}$ included in the conductivity equations. It is found that the full Pedersen conductivities are also not equal to the usual, zero-frequency Pedersen conductivity, although the issue mostly affects the Alfvén wave. However, from Eq. (4), it is the wave-Pedersen conductivity, and not the full Pedersen conductivity that enters the single-mode electrostatic criteria (4); and according to Fig. 1g the criteria are not satisfied for either mode.

### Model validation

Without actually constructing the cascaded transmission line model we have learned, in Section “Findings using only the eigenvectors and eigenvalues”, that it will not and should not recover electrostatic theory. Therefore, to validate the model we will modify the Alfvén and Whistler waves so that the electrostatic criteria (4) are satisfied, and see if we can recover electrostatic theory using these modified waves in the cascaded transmission line model. Hence, Fig. 2b–e shows results where, in Eq. (9), the *z*-directed wavelengths $(2\pi /k_{0jz})$ for both modes have been increased to $5\times 10^{8}$ m, the z-directed dissipation scale lengths $(l_{dj})$ have been reset to $\infty$, and the *y*-components of the complex electric fields (in $\vec {h}_{j}$) have been rescaled to make the wave-Pedersen conductivities equal the zero-frequency Pedersen conductivity (with our convention $(k_{x}=0)$ the characteristic admittance for each wave-mode is of the form $Y_{0}=j_{\parallel }/\vec {\nabla }\cdot \vec {E}_{\perp }=-{\tilde{B}}_{x}/(\mu _{0}{\tilde{E}}_{y})$, where the displacement current is ignored^32^, and the tildes are used to indicate the complex electric and magnetic fields). The remaining modal properties are not modified, and the validation results are found to be independent of the starting point for the modifications, at least within the parameter space addressed in this article. The length of the transmission-line sections is one kilometer for all of the results presented below.

Figure 2b shows the downward-looking conductance as a function of altitude (blue line, solid for real part and dashed for imaginary part) together with the field-line-integral of $\sigma _{P}$ over the region up to that altitude (dashed red line). It is seen that the dashed red line lies exactly on top of the solid blue line, while the dashed blue line is at zero, meaning that the electrostatic prediction for conductance is exactly reproduced.

Figure 2c shows the *y*-component of the instantaneous electric field ($E_{y}$, blue line), and the *x*-component of the instantaneous magnetic field ($B_{x}$, red line), as a function of altitude. The time instant is chosen to be the one when the downward Poynting flux maximizes, and the units for $E_{y}$ and $B_{x}$ are arbitrary, so that they may be placed on the same axis with the same scale. With our conventions $B_{x}$ is, essentially, the parallel current, although with a $90^{\circ }$ phase offset. It is seen that $E_{y}$ maps perfectly through the ionosphere, while the parallel current falls off to zero, in perfect conformance with electrostatic theory.

Figure 2d illustrates the complexity of the modal interaction that occurs in spite of the good behavior of $E_{y}$ and $B_{x}$. Shown are the contributions to $E_{y}$ from the Alfvén (blue line) and Whistler (green line) waves, along with the resultant (dashed red line). It is seen that the Whistler wave makes a significant contribution in the lower E-region, and there is a kinky feature.

Figure 2e illustrates the apparent cause for the kinky feature. Shown is the complex-conjugate dot-product of the (normalized) eigenvectors for the two modes, and it is seen that they become nearly aligned right at the altitude of the kinky feature. This altitude also corresponds to the kink in parallel wavelength for the Whistler wave that can be seen in Fig. 1e (although with different density), and this plot also shows that the kink is below the propagation-cutoff altitude (upper end of the red trace, and in other examples the separation is quite a bit more). The sharpness of the alignment and parallel-wavelength features both mirror the sharpness of the kinky feature, even though the former are both derived independently of the cascaded transmission line model. The extreme sharpness of the alignment feature begs the question of whether there may actually be a degeneracy in $H_{5}$ at this altitude, although our numerical approach cannot truly address this question. Regardless, the near-alignment can be expected to produce rapid mode mixing. The effect of this mode mixing has been examined in detail in the Supplementary Information (Fig. 6, leftmost panels), where it was shown that the scale for variations can become much less than a kilometer, and this seems to be a good explanation for the kinky feature.

It turns out that electrostatic theory cannot be reproduced when the waves have different wavelengths, because in this case, in-order to have the same wave-Pedersen conductivity they must have different $Y_{0}$, and so the rapid mode-mixing can produce a rapid change in the downward-looking conductance. This mode-mixing effect figures prominently in the results to follow. That the near degeneracy and associated kinky features can exist without corrupting the accurate agreement with electrostatic theory, in the equal-wavelength synthetic case shown in Fig. 2b–e, is strong evidence that the cascaded transmission line model is implemented correctly and with sufficient numerical accuracy.

### Model results

Turning to the results when the real waves are used in the cascaded transmission line model, Fig. 2f shows a compilation of results for two different transverse wavelengths (100 km and 1000 km, top and bottom, respectively), and two different electron densities ($4.7\times 10^{9}\,\text {m}^{-3}$ and $1.0\times 10^{11}\,\text {m}^{-3}$, left and right, respectively, and constant with altitude). Results are shown for conductance as well as for $E_{y}$ and $B_{x}$, for both the two-mode model, and also, for pedagogical purposes, for models using just one of the two modes in the cascade of transmission lines. However, $E_{y}$ and $B_{x}$ are not shown for the Whistler-wave only case, since it does not propagate above the E region, and so by itself does not support conductance at any appreciable distance above the E region. (See the Methods Section “Methods” about modeling the Whistler wave above its cutoff altitude.) In order to accommodate the single mode models we must drop two of the boundary conditions, and so the single mode models do not enforce continuity of $E_{x}$ or $B_{z}$.

The top left quadrant of Fig. 2f shows the results for the shorter wavelength and lower density. In the case of conductance (leftmost panels), it is seen to agree quite well with electrostatic theory (blue and dashed-red lines are nearly coincident), for both the two-mode model, and also for the model using only the Alfvén wave (only real part is shown). A kinky feature can be seen in the two-mode case, but it is low enough in altitude so as not to affect the conductance very much. However, in neither case does the electric field map unchanged through the ionosphere (rightmost panels, blue lines), and this is especially true in the two-mode case, where the kinky feature is associated with a cutoff of $E_{y}$.

The bottom two quadrants of Fig. 2f show the results for the longer wavelength. In this case the conductance (leftmost panels in both quadrants) is found to be much less than as calculated using electrostatic theory (blue as compared with dashed-red lines, a 60% to 80% reduction). This is true in all of the cases, although the reduction in the two-mode case is a little more than in the Alfvén-only case. Some additional understanding can be gleaned from the plots of electric field (rightmost panels in both quadrants, blue lines), where it is seen that now, for all cases, the electric field is cutoff before reaching the bottom of the E region. So the electric field cutoff happens even without mode mixing, in which case it appears that the Alfvén wave is reflected by rapidly changing $Y_{0}$ (this has been tested by artificially increasing $l_{dj}$, to rule out dissipation). However, the cutoff happens earlier and is sharper for the two-mode cases.

Detailed analysis described in the Supplementary Information (Section 7.3) finds that the sharpened cutoffs are associated with rapid mode-mixing in the vicinity of the kinky features, caused by the rapid and strong alignment of the Alfvén and Whistler waves, as was already mentioned in the discussion of Fig. 2e. The altitude of this strong alignment, which appears to represent a near degeneracy of the two waves, constitutes an effective bottom of the conducting ionosphere (as seen from above). Discussion of the additional kinky features that occur in one of the conductance curves can also be found in the Supplementary Information (Section 7.3).

Perhaps the most interesting of the results is shown in the top-right quadrant of Fig. 2f, which shows the shorter-wavelength and higher-density case. The significant phase rotation that was discussed in reference to Fig. 1f appears to be producing a resonance of the ionosphere, where both the electric field (rightmost panels, blue lines) and the conductance (leftmost panels, blue lines) pass through zero. This effect can be understood as a generalized manifestation of the tangent function behavior of conductance for an open circuited transmission line, which was given in Eq. (3). Indeed, for the two-mode case the conductance curve looks very much like a tangent function, although with the infinities suppressed as would happen if the argument had a small imaginary part. The small imaginary part is caused by wave dissipation, so that the electric field of the reflected wave can never completely cancel that of the incident wave.

Although the Alfvén-only case does not look as much like a tangent function, it still passes through zero. The fact that the resonance occurs also in the Alfvén-only case shows that it has nothing to do with mode mixing, and is mostly just a feature of the short parallel wavelength of the Alfvén wave in the highly-collisional E region.

To understand the resonance energetically it is important to remember that a plasma also supports non-electrical forms of energy, such as kinetic energy. Energy may pass back and forth between the electrical and non-electrical forms, and so in following only the electrical form we cannot expect energy conservation to hold^33^. For example, Cosgrove-2016^3^ (their Table 3) found a plasma resonance associated with a reversal in the relative direction of the electric field and ion velocity, such that the Pedersen conductivity became negative, with ion kinetic energy being converted into electrical energy.

## Discussion

### Summary of results

Specific results have been presented in Section “Model Results”, such as the greatly reduced conductance at longer scales. However, the most reliable results are of a general nature. Transmission line theory is very useful for designing electrical (usually microwave) circuits because it allows for understanding how they work, and therefore how they can be tuned. In bringing this theory to the ionosphere it is important to distinguish between the detailed results that are presented herein as examples, and the physical conclusions that are far more robust.

We have found that (A) the wavelength of the propagating modes becomes very short in the E region, such that phase rotation should sometimes be important; (B) the ionospheric conductance is not determined by either the usual (zero-frequency) or full Pedersen conductivity, but is instead determined by the wave-Pedersen conductivity in Eq. (4), which is often smaller (Fig. 1g); (C) a second propagation mode arises in the *E* region, with the two modes becoming nearly degenerate at what appears to be a single specific altitude (specific collision frequency); and (D) the electrostatic-wave theory does not support the Whistler wave, and the parallel-wavelength for the Alfvén-wave analogue is orders of magnitude too large. These four conclusions follow directly from the electromagnetic fluid equations (i.e., their eigenvalues and eigenvectors), and do not rely on the cascaded transmission-line model, which is used only to make the results more quantitative: for example, to calculate the conductance, and to show that the near degeneracy leads to rapid mode-mixing with associated significant effects.

### Applications in global modeling

The results modify the way that the ionospheric conductance is computed from a known vertical profile of electron density, and inject an important scale-size dependence. While these results suggest some possible improvements to MI coupling algorithms, there is more work needed to make these improvements practical.

The ideal approach would be to implement dynamical filtering according to scale size, so that the signal modeled on the inner boundary of the magnetosphere can be properly loaded by the scale-size-dependent conductance. However, this proposition runs into the problem of the complex behavior of the auroral acceleration region, and the modifications to scale-size that it may introduce for a signal passing through. Thus it might be preferable to begin with a simplified approach, such as to identify a characteristic scale-size for the interaction, and use it to inform a single conductance quantity to be inserted into Eq. (1).

Both of these approaches suggest the need for observational studies of the scale-sizes associated with MI coupling. These might derive from studies of Poynting flux measured by satellites^28,34^. Because Poynting flux represents the transfer of electromagnetic energy, the scale-size distribution for Poynting flux events should be representative of the scale-size distribution for the MI coupling interaction.

However, our results also indicate that there is considerable complexity and/or sensitivity associated with calculating conductance from ionospheric parameters alone, such as might be measured by incoherent scatter radar (ISR). This suggests that there should also be an effort to develop techniques that actually measure conductance directly, from above the ionosphere. These techniques might involve constellations of satellites capable of sampling a sufficiently large region, such that the signal composition can be determined and processed for input admittance^35^.

### Validation and roll for observational science

The calculation presented is more general than electrostatic theory, and includes the latter as a special case, which we have found does not normally arise in the ionosphere. Much of this conclusion follows directly from computation of the eigenvalues and eigenvectors of a matrix, where the matrix is simply an exposition of the Maxwell and fluid equations. So given that the algorithms for calculating eigenvalues and eigenvectors are very well established, it would seem that the appropriate question for observational science is *not* “Is electrostatic theory nevertheless correct?”, because such a result would contradict our most basic theories. Rather, the appropriate question is “What kinds of effects might we observe given the new understanding?”

In this regard we should be somewhat careful, because there is a good deal of parameter space to be explored. In this paper we have presented results for 100 km and 1000 km transverse wavelength signals, and in this case we have found that the shorter wavelength signal actually penetrates deeper into the ionosphere (Fig. 2f), even though its parallel wavelength is shorter. If this trend continues to smaller scales it may produce an observable effect in the shifting of the electric-field spatial-spectrum with altitude.

On the other hand, it might be that this baseline, scale-size dependent ionospheric response is associated with a shortening of the scale-size for MI coupling, which affects all altitudes in the ionosphere. There may be coupling to processes in the auroral acceleration region, manifesting as auroral arcs, and etc. In this case we cannot really expect to find an ionospheric condition in the auroral region that can be reliably analyzed using our steady-state, horizontally uniform calculation. In this case, it could be that validation efforts are better addressed to lower latitudes, where the field lines spend more “time” in the *E* region. There is a lot of ground to cover here, and a good topic for additional research. But at any rate, our results do motivate discovery science in the auroral region.

We should seek to evaluate the degree to which electric fields map along magnetic field lines from just above the *E* region, down through to its very bottom, and we should do this as a function of scale size. This question is central to the question of how much of the large amount of conductivity that exists in the lower *E* region, actually contributes to the ionospheric conductance seen from above. While ISR is the main instrument for this region, it unfortunately cannot answer this particular question, because there are persistent sheared winds that dominate the ion-drift^36^. Instead, data from rocket experiments seems more promising. But here there is also a problem in that rockets do not follow magnetic field lines. Nevertheless, experiments involving multiple rockets might provide an important window into the mapping, or not, of electric field through the lower *E* region; especially when there is supporting contextual information such as ISR and/or all-sky (optical) data.

## Conclusion

Overall, these results create an unexpected situation where any experimental finding that seems to support electrostatic theory, over the results presented here, would itself have unexpected implications. One possible explanation might be that non-linear effects somehow produce a state that resembles the electrostatic state. It is hard to imagine that horizontal inhomogeneity could somehow have this effect. And the only other alternative would seem to be a finding against the fluid equations themselves, that is, a kinetic effect, possibly relating to the (near?) degeneracy.

But since none of these alternatives seems very likely, our final conclusion must be a prediction that the physics of the collisional ionosphere is not well-described by the traditional electrostatic paradigm (above the $\sim$ $100$ m scale), and that it actually involves significant phase-rotation, mode-mixing, and conductivity effects. These all lead to an altered ionospheric-conductance, and thus to modification of the magnetosphere-ionosphere interaction. Transmission line theory provides an understanding of the effects, and allows for quantitative calculations for the vertically-inhomogeneous geometry.

## Methods

This Methods section is introduced in Sections “Gedankenexperiment and criteria for electrostatic theory” through “Extension to a vertically inhomogeneous ionosphere” above.

### Deriving transmission line theory from the general solution, intuitive and formal

We begin by deriving the general linearized solution for a uniform collisional plasma described by fluid equations, which turns out to produce a generalized form of transmission line theory. Taking the Fourier transform in space, the electromagnetic fluid equations may be expressed in matrix form as,

5 $$\begin{aligned} \frac{\partial \vec {X}}{\partial t}-iH_{5}\vec {X}=\vec {F}(t), \end{aligned}$$

where the matrix $H_{5}$ has been given in the previous work, both for the 5-moment fluid equations (Fig. A.1 of the Supplementary Information), and for the reduced set where the continuity and energy equations are omitted (Equation 7 of Cosgrove-2016^3^). $\vec {F}(t)$ is a time-dependent vector containing the nonlinear terms. As they are for electrostatic theory, the nonlinear terms will be dropped, meaning that we will describe the evolution of small variations about a background state that is assumed to satisfy the electromagnetic fluid equations. Initially we will treat a homogeneous plasma, in which case the background state is thermal equilibrium, which is in fact an exact solution of the electromagnetic fluid equations. The solution for a homogeneous plasma will then be extended to a solution for the vertically inhomogeneous ionosphere, in which case the background state becomes a heuristic representing the observed state of the ionosphere, which may or may not be adequately described by the equations of motion (5). (See Section 5 of the Supplementary Information for more details.) This same heuristic is used in the development of electrostatic theory.

Without the nonlinear terms, the exact solution for the equations of motion (5) can be written,

6 $$\begin{aligned} \vec {X}\left( t,\vec {k}\right)= & {} \sum _{j=1}^{16}a_{0j}(\vec {k})\,\vec {h}_{j}(\vec {k})\,\text {e}^{i\omega _{j}(\vec {k})t},\;\text {or, taking the inverse Fourier transform,}\nonumber \\ \vec {X}\left( t,\vec {r}\right)= & {} \sum _{j=1}^{16}\int \text {d}^{3}k\,a_{0j}(\vec {k})\,\vec {h}_{j}(\vec {k})\,\text {e}^{i\left( \omega _{j}(\vec {k})t+\vec {k}\cdot \vec {r}\right) }, \end{aligned}$$

where $\vec {h}_{j}$ and $\omega _{j}$ are the eigenvectors and eigenvalues of $H_{5}$, respectively, $\vec {k}$ is the Fourier transform variable (the wavevector), and the $a_{0j}$ are 16 arbitrary functions of $\vec {k}$. The number 16 arises for the case of the 5-moment fluid equations, which together with the Maxwell equations comprise 16 scalar equations. The solution is easily verified by direct substitution. Because these are physical equations, we will assume that $H_{5}$ is diagonalizable, and so its eigenvectors are linearly independent, and the solution (6) is the complete initial-value solution^37^; an arbitrary initial state can be fixed by choosing the 16 $a_{0j}(\vec {k})$, and then evolution consists of 16 independently evolving eigenmodes. However, we do find evidence for a possible degeneracy of $H_{5}$ that occurs at one particular collision frequency, and whether this is actually true, and what might be its meaning, is a question for future research. Regardless, the solution (6) applies “almost everywhere,” and this shows that, generally speaking, any sufficiently-small disturbance can be described as a sum of independently evolving waves.

In order to evaluate the conductance we will set the wind to zero in the fluid equations, and will not include any background electric field. In this case the frequencies $\omega _{j}$ are complex numbers with positive imaginary parts, so that the disturbance (6) obeys the physical constraint that it decays over time. These are source free equations, and so there cannot exist a steady-state solution that dissipates energy.

However, since electrostatic theory is generally applied to driven cases where there is a steady-state solution that dissipates energy, the solution (6) is not directly applicable to testing electrostatic theory. The relevance of the solution (6) to electrostatic theory was discussed in Cosgrove-2016^3^, where it was suggested to add a causal source that may be imagined as an antenna with transmitter that turns on, and then continues transmitting indefinitely. This source can represent either physical processes in the magnetosphere, E-region dynamo activity, or any other physical source. Over time, the response to the source may approach a steady state, which may or may not be described by electrostatic theory (See, for example, Sections 1 and 7 of Cosgrove-2016^3^).

Thus, it is the steady state response to this source that needs to be characterized, and which should be evaluated to see whether it conforms to electrostatic theory. This source may be formalized as,

7 $$\begin{aligned} \vec {f}_{A}\left( t,\vec {r}\right) =\vec {A}u(t)\text {e}^{i\omega _{0}t}\delta (z)\text {e}^{ik_{0y}y}, \end{aligned}$$

which is a harmonic and planar source that turns on at $t=0$, oscillates with real temporal frequency $\omega _{0}$, oscillates in the *y* direction with real wave-vector $k_{0y}$, and is localized in the *z* direction (*u*(*t*) is the unit step function and $\delta (z)$ is the Dirac delta function). Note that this source can be thought of as a large planar antenna that is selective of the transverse wavevector ($\vec {k}_{\perp }={\hat{y}}k_{0y}$). The response to this particular source can be considered as a Fourier component of the response to a general planar source.

As explained in Cosgrove-2016^3^, in order to understand the response to the source (7) it is useful to consider a case where the transmitter turns off again, after transmitting for a couple of cycles. Shortly after turnoff, and at any significant distance from the antenna, there will remain only localized disturbances consisting of the modes that are able to propagate at the transmitter’s frequency and wavelength ($\lambda _{\perp }=2\pi /k_{0y}$, the transverse wavelength). Finding the set $\left\{ a_{0j}\right\}$ that fits the initial-value-solution (6) to the disturbances should produce a superposition of a few propagating wave-packets centered on wavevectors $\vec {k}_{0j}$ such that each $\omega _{jr}(\vec {k}_{0j})=\text {real}\left( \omega _{j}(\vec {k}_{0j})\right)$ matches the transmitter frequency, $\omega _{0}$, and the transverse part of each $\vec {k}_{0j}$ matches the antenna wavelength. (Note that while the geomagnetic field has for convenience been assumed perpendicular to the plane of the source, this is not a necessary assumption, and by “transverse” we actually mean in the plane of the source).

Thus, using the standard interpretation of wave-packet propagation (top panel of Fig. 1b), we might imagine the subsequent evolution as the superposition of a few independently evolving wave packets centered on wavevectors $\vec {k}_{0j}$, propagating with group velocities $v_{gjrz}=\left. -\left( \partial \omega _{jr}/\partial k_{z}\right) \right| _{\vec {k}_{0j}}$, dissipating with time scales $\omega _{ji}^{-1}=1/\text {imag}\left( \omega _{j}(\vec {k}_{0j})\right)$, oscillating with frequencies $\omega _{jr}(\vec {k}_{0j})=\omega _{0}$, and disturbing the plasma with polarization vectors $\vec {h}_{j}(\vec {k}_{0j})$. If the transmitter did this repeatedly, that is, if it did not turn off, there would eventually result a steady-state disturbance around the source that decreases with distance according to the dissipation scale length $v_{gjrz}/\omega _{ji}$ (for the *j*th mode). The evolution to this steady-state is depicted in the second and third panels of Fig. 1(b).

This interpretation has been formalized in the Supplementary Information (Section 4), where the exact (linearized) driven steady-state solution with the source (7) placed on the right hand side of Eq. (5), has been derived as,

8 $$\begin{aligned} \vec {X}\left( t,\vec {r}\right) =\text {e}^{i(\omega _{0}t+k_{0y}y)}\frac{i}{2\pi }\sum _{j=1}^{16}\int \limits _{-\infty }^{\infty }\text {d}k_{z}\frac{\vec {h}_{j}(0,k_{0y},k_{z})a_{j}(0,k_{0y},k_{z})}{\omega _{j}(0,k_{0y},k_{z})-\omega _{0}}\text {e}^{ik_{z}z}, \end{aligned}$$

where $\vec {a}=U^{-1}\vec {A}$, and *U* is the matrix having the eigenvectors of $H_{5}$ as columns. From this solution we can verify the harmonic oscillation of the entire plasma in *t* and *y*, and that the solution consists of a sum of 16 independent modal contributions. And to emphasize, Eq. (8) is the complete solution, which necessarily includes any DC or electrostatic components. The temporal and/or spacial frequencies may be zero, although in practice we will confine ourselves to analyzing problems that are “pseudo DC.”

The Supplementary Information (Section 4) includes an extensive discussion of how the wavepacket interpretation can be recovered from the exact form (8). Essentially, the integrands are assumed peaked around $\omega _{jr}(0,k_{0y},k_{z})=\omega _{0}$, so that the residue theorem can be applied with the $k_{z}$ dependence of $\omega _{j}$ linearized, and $\vec {h}_{j}$ and $a_{j}$ stationary. If the imaginary part of the group velocity is ignored the residue theorem recovers the usual wavepacket interpretation.

The Supplementary Information also describes a validation of this result that was done by evaluating a selection of the integrals numerically and comparing the critical properties of wavelength, dissipation scale length, and polarization vector. The results of this validation are highly satisfactory, although there are some deviations that are judged to be either inconsequential or not applicable, and there is additional discussion in the Supplementary Information (Section 4) .

The solution (8) also includes integrals for non-propagating modes, that is, modes that cannot satisfy $\omega _{jr}(0,k_{0y},k_{z})=\omega _{0}$ for any $k_{z}$. In this case the integrands are not peaked around any particular $k_{z}$, and so the residue theorem cannot be applied. Instead, the integrals may be viewed as Fourier transforms of very broad functions, which, of course, should produce very narrow functions. Thus, the non-propagating modes cannot transport energy any significant distance, and are associated only with near-zone or localized boundary effects. Therefore we will omit these modes and Section “Localized boundary effects” gives some additional discussion.

Combining these results provides that the driven steady-state solution for the source (7) can be well approximated by,

9 $$\begin{aligned} \vec {X}\left( t,\vec {r}\right) =\text {e}^{i(\omega _{0}t+k_{0y}y)}\sum _{j=1}^{2N}\vec {h}_{j}(\vec {k}_{0j})\varepsilon _{j}\text {e}^{i\left( k_{0jz}z+iz/l_{dj}\right) }, \end{aligned}$$

where the $\left\{ \varepsilon _{j}\right\}$ are arbitrary complex constants, $l_{dj}=\left. v_{gjrz}/\omega _{ji}\right| _{\vec {k}_{0j}}$ is the (signed) dissipation scale length for the *j*th mode, *N* is the number of propagating wave-modes, and as above, $\vec {k}_{0j}$ is the wavevector such that $\omega _{jr}(\vec {k}_{0j})=\omega _{0}$. The sum ranges over 2*N* because each wave-mode can propagate in two opposing directions, and so is associated with two modes $(k_{0jz}\leftrightarrow -k_{0jz})$. The rightward propagating modes occur on the right side of the source, and the leftward propagating modes occur on the left side. For a section of line embedded in a circuit both directions will be present, and so by writing the solution in the form (9) we obtain a generalized form of transmission line theory.

For the case when $N=1$ the result (9) is a superposition of two oppositely propagating modes, and so the above can be regarded as a derivation of the usual transmission line theory from electrical engineering. Transmission lines such as waveguides and coaxial lines and microstriplines are designed so that only a single wave-mode is supported, where in the latter two cases $k_{0y}=0$, and in the former case $k_{0y}$ is selected by the transverse dimensions of the waveguide (and care must be exercised not to excite higher order modes). In the case of the ionosphere, $k_{0y}$ is an independent variable that labels the Fourier component to be analyzed. And, in the case of the ionosphere, we must sort through the 8 potential wave-modes to see which are capable of propagating for typical ionospheric frequencies and transverse scale sizes.

### Sorting the modes

By virtue of the fact that they are comprised of 16 scalar equations, the electromagnetic 5-moment fluid equations can support up to 8 wave-modes. However, we find two evanescent modes $(\text {i.e.,}\,\omega _{jr}(0,k_{0y},k_{z})=0)$ that do not form a wave-mode, and so are not capable of transmitting energy. In addition there are the *X*-, *O*-, and *Z*-mode radio-frequency waves, which are way too high in frequency to contribute to ionospheric phenomena (i.e., are non-propagating, cannot satisfy $\omega _{jr}(0,k_{0y},k_{z})=\omega _{0}$ for any $k_{z}$). Thus we are left with 4 wave-modes that can potentially contribute to ionospheric science, which we choose to call the Whistler, Alfvén, Ion, and Thermal waves. The Ion wave, which is short for ion-acoustic wave, was not present in the Cosgrove-2016^3^ study because the continuity equations were not included. And the Thermal wave arises when the energy equations are added, and so is not one of the more commonly studied physical waves.

An alternative naming scheme that is better aligned with MHD might be the Fast-, Shear-, and Slow-mode Alfvén waves, together with the Thermal wave. However, Cosgrove-2016^3^ (Section 3) found that the highest frequency wave was a better physical fit to the Whistler wave than to the Fast-mode wave (both collisionless waves), in the lower E region where it primarily manifests. Thus, we will employ the former of the two naming schemes, which is anyway better aligned with the usual terminology from ionospheric physics. It is important to understand that in this work we employ a formal definition of waves as eigenmodes of the electromagnetic 5-moment fluid equations, which are identified over altitude. This definition is not fully equivalent to the more traditional approach that proceeds through simplifying the equations for specific physical cases, and which may result in more different kinds of waves, which may not exist at all altitudes. Hence it is actually more appropriate and helps to avoid confusion if the names we choose do not match exactly to any of the physical naming schemes.

In order to find out which of the four wave-modes we need to include we will calculate their eigenvalues numerically, and investigate their properties within a characteristic range of ionospheric frequencies and scale sizes. To do this we will consider a range of transverse scale-sizes from 100 km to 1000 km, and then calculate the frequency based on a representative 40 m/s transverse drift velocity (i.e., $40\,\text {m/s}=\omega _{0}/k_{0y}$, and these frequencies are all low enough that we may consider them as pseudo-DC). In doing this we will assume that the geomagnetic field is in the *z* direction, and so at this point the term “transverse” comes to mean both in the plane of the source and perpendicular to the geomagnetic field.

The result of this activity is the finding that the Ion and Thermal waves both have dissipation-scale-lengths less than 10 km, and generally much less, so that neither of these waves is capable of transmitting energy through the ionosphere. (See panels b, d, and f in Figure 2 in the Supplementary Information.) We have tried employing all four wave-modes in the model, but the Ion and Thermal waves do not seem to have much of an effect. And this is a good thing for electrostatic theory, since if they were to couple significantly into the motion their rapid dissipation would prevent the mapping of electric field that electrostatic theory predicts. The same goes for all of the non-propagating modes that we did not include; they can only serve to remove energy from the propagating modes that are doing the work of transporting the electric field through the ionosphere. Thus, we conclude that an appropriate model can be derived by taking $N=2$ in Eq. (9), where the two wave modes to be included are the Whistler and Alfvén waves.

### Extending the whistler wave

To make Fig. 2 the Whistler wave has been extended to altitudes above where it becomes cutoff (i.e., cannot satisfy $\omega _{jr}(0,k_{0y},k_{z})=\omega _{0}$ for any $k_{z}$) using the quadratic dispersion relation derived in the Supplementary Information (Eq. B.3). Although not necessary, this extension is usually employed in the model in order to avoid an anomaly that occasionally occurs across the section-boundary where the Whistler wave becomes cutoff, which otherwise requires a change in the number of enforced boundary conditions. As long as the extended part of the Whistler wave makes a negligible contribution at higher altitudes, we deem this to provide a better approximation to the physical behavior across this boundary, near which even the cutoff Whistler wave may have some effect. The extension is not related to the kinky feature, which occurs below the cutoff altitude.

### Localized boundary effects

Transmission line theory involves a tradeoff where localized boundary effects are ignored in exchange for a simpler physical description that can be obtained with less knowledge of the boundary conditions. The omission of non-propagating and highly-dissipative modes from the sum in the general solution (8) makes it possible to obtain a unique solution using the reduced set of boundary conditions that we actually know. In the case when the adjacent sections are the same we can deduce that the omitted modes are not excited by the boundary, because the driven steady-state solution (8) remains valid across the boundary. This means that the complete set of physical boundary conditions are, in fact, satisfied, even though we only enforced a smaller set.

But when the adjacent sections are not the same enforcing the smaller set will not lead to complete satisfaction of the full set. In order to correct this situation, some of the modes that were dropped from the general solution will actually be excited around the boundary, with the level of excitation decreasing to zero as the adjacent sections become more similar. Thus we can see that transmission line theory sacrifices accuracy right around the boundaries in hope of achieving accuracy in the regions away from the boundaries. And with respect to the latter regions, transmission line theory involves an approximation where the additional excited modes do not disrupt the boundary conditions with respect to the dominant, energy carrying modes.

To understand the approximation, consider that the omitted modes all have a very narrow response (Section “Deriving transmission line theory from the general solution, intuitive and formal”), and so must be confined to regions right around the boundaries where they are excited. So if these modes did disrupt the boundary conditions it would mean that there are, for example, discontinuities in the transverse electric field or parallel current that happen over very narrow regions. But we have chosen these boundary conditions exactly because it is physically very difficult for these quantities to change over very short distances. We do not expect such discontinuities, and, by the way, electrostatic theory does not predict them either. Therefore it would seem that omission of the localized boundary effects and associated modes should be a useful approximation that, if anything, produces results that resemble electrostatic theory more than they actually should.

### Supplementary Information

Supplementary Information.

## Data Availability

The data used as input for testing the calculation has been published in Cosgrove-2016^3^ (https://doi.org/10.1002/2015JA021672), and is reproduced here in Fig. 2a. An electronic copy is available upon request.

## Code availability

The Python/NumPy code for the model is available upon request.
