# Supplementary material for: On an electromagnetic calculation of ionospheric conductance that seems to override the field line integrated conductivity
Source: Sci Rep. 2024 Apr 2;14:7701. doi: 10.1038/s41598-024-58512-x (PMC11369143; doi:10.1038/s41598-024-58512-x)
Supplement: Supplementary file 1 — Supplementary Information. [file 41598_2024_58512_MOESM1_ESM.pdf]

Supplementary Information for "On an Electromagnetic Calculation of Ionospheric Conductance that seems to Override the Field Line Integrated Conductivity."

by Russell B. Cosgrove

Contents

1. 50 page preprint that serves as a detailed manual for the calculation.
2. Supplementary information for item 1.

Item 1 of Supplementary Information: 50 page preprint that serves as a detailed manual for the calculation.

An Electromagnetic Calculation of Ionospheric Conductance that seems to Override the Field Line Integrated Conductivity

Russell B. Cosgrove
November 19, 2022

Center for Geospace Studies, SRI International, Menlo Park, CA, USA
russell.cosgrove@me.com

Abstract We derive a steady-state, electromagnetic solution for collisional plasma and apply it to computing the total conductance for a vertically stratified ionosphere interrogated by a 3D signal with finite transverse wavelength, which we compare to the field-line-integrated conductivity, finding significant differences on all scales investigated. The approximate solution is derived from an exact linearization of the electromagnetic 5-moment fluid equations, and writing down the integral form of the driven steady-state solution, which is expressed as a sum over modal contributions associated with the eigenvectors/eigenvalues of the set of equations. We find the eigenvectors/eigenvalues numerically and use them to argue that only two of the modes are capable of transmitting energy through the ionosphere. Approximating their integrands with analytic functions yields a solution valid for homogeneous plasma, expressed in terms of the eigenvectors/eigenvalues for the two modes. The solution may be understood using wavepacket concepts. Transmission line theory is then used to extend the homogeneous-plasma solution to a solution for the vertically-stratified ionosphere. We review how transmission line theory contains electrostatic theory as a special case, and show that our solution reproduces electrostatic theory exactly when the eigenvectors/eigenvalues have the right properties, which requires that they be artificially modified. Otherwise we find short-wavelength, mode-mixing, and wave-admittance effects with broad relevance to ionospheric science, and to global modeling of the Sun-Earth system. Since resonant systems can be very sensitive, quantitative accuracy may require tuning, but the physical conclusions seem unavoidable and to affect all transverse scales.

Key Points

- An electromagnetic calculation of the ionospheric conductance finds something quite different from the field line integrated conductivity
- The electric field does not map through the ionosphere, and the penetration depth is highly dependent on scale and density
- There are effects from short parallel wavelengths and multiple modes, such that wave-like and resonant behavior are supported

© 2022, CC BY SA license

1 Introduction

Electrostatic theory is an extremely convenient, steady-state simplification of electromagnetic theory used extensively in electrical engineering (e.g., resistors, capacitors, and inductors), and also in the physics of collisional plasmas, such as the ionosphere. Transmission line (TL) theory is also a steady-state theory, which applies to physical systems that cross the boundaries of scale where electrostatic theory becomes inapplicable [e.g., *Collin*, 1966; *Malherbe*, 1979]. Thus, TL theory allows for testing electrostatic theory which, when applicable, arises from the long-wavelength limit of TL theory. However, TL theory is generally applied to engineered physical systems where only one mode of propagation is supported and the transverse dimension is suppressed. Specifically, applications of TL theory to the ionosphere have been very limited [*Mallinckrodt and Carlson*, 1978; *Bhattacharyya and Burke*, 2000], and as a result, no rigorous criteria

have been developed for use of electrostatic theory in the ionosphere [*Vasyliunas*, 2012; *Cosgrove*, 2016]. In this work we examine the validity of electrostatic theory in the ionosphere using a bi-modal form of TL theory for collisional plasma, and derive a steady-state electromagnetic solution for the vertically-inhomogeneous ionosphere interrogated by a plane-wave signal with finite transverse wavelength (i.e., the Fourier components of a 3D signal). We show how this solution would reproduce electrostatic theory if the collisional waves in the ionosphere had the right properties. However, we find that the ionospheric waves do not generally have the right properties. In fact, there is a wide range of parameter space where the TL theory predicts profound wave-like effects that should have important systemic consequences. For example, the ionospheric conductance is different from the field line integrated conductivity.

Hence, we find that an electromagnetic theory of the ionosphere is required, and we put forth our TL theory as the most natural generalization of the current electrostatic baseline. Although needing to be expanded in terms of the parameter space that it covers, this TL-theory calculation amounts to a rigorous, electromagnetic theory of the ionosphere that can replace electrostatic theory in many applications, and which can be used to validate the numerical simulations being pursued by others. By virtue of its connection to circuit theory, the TL theory also provides a physically intuitive description of the electrical steady-state of the ionosphere, which appears to be much more interesting than previously expected. (It should be noted that the author is a former microwave filter engineer who formed a deep appreciation for TL theory through practical application.)

Disambiguation: This article is addressed primarily to ionospheric physics, which traditionally employs a version of electrostatic theory that is very close to the textbook theory [e.g., *Jackson*, 1962, 1975], where all time derivatives are set to zero and what remains of the equations of motion are linearized and applied to a boundary value problem [*Farley*, 1959; *Farley*, 1960; *Spreiter and Briggs*, 1961; *Kelley*, 2009]. By "electrostatic theory," or "full electrostatic theory," for emphasis, we refer to the practice of assuming that this solution is an approximation for the linear, steady-state solution found using the full equations of motion. One of the major electrostatic concepts we will test is the "Farley factor" [e.g., *Kelley and Gelinas*, 2000; *Kagan et al.*, 2000], which provides a way to assess the transverse-scale-size dependence of the distance that the influence of a disturbance will extend along geomagnetic field lines, for a uniform plasma. Specifically, this "mapping distance" is estimated as the transverse scale-size multiplied by the Farley factor, which is often referred to as the Farley mapping distance, $d_F = \sqrt{\sigma_0/\sigma_{P0}}/k$ (where $k = \left|\vec{k}\right|$ is the transverse wavenumber, and σ_0 and σ_{P0} are the field-aligned and zero-frequency Pedersen conductivities, respectively). This concept is used to estimate the degree of coupling between different regions of the ionosphere, and detailed electric field distributions may be calculated by solving an associated boundary value problem for the actual inhomogeneous plasma [e.g., *Kelley*, 2009 and references therein]. The same concept also gives rise to the idea that the ionospheric conductance can be calculated by integrating the local conductivity along geomagnetic field lines, which is essentially an assumption that the electric field maps unchanged through the whole of the ionospheric thickness. By solving the associated boundary value problem, it has been determined that this should be true if the transverse-scale-size of the imposing signal is greater than about 10 km [e.g., *Farley*, 1960; *Spreiter and Briggs*, 1961; *Hysell et al.*, 2002]. Similar mapping assumptions and boundary-value calculations underly much of ionospheric physics, including analysis of auroral arcs [e.g., *Marklund et al.*, 1982; *Aikio et al.*, 2002; *Juusola et al.*, 2016], analysis of equatorial plasma bubbles [e.g., *Tsunoda et al.*, 1982; *Aviero and Hysell*, 2012; *Tsunoda*, 2015], analysis of the pre-reversal enhancement [e.g., *Farley et al.*, 1986; *Eccles et al.*, 2015], analysis of sporadic E layers [e.g., *Haldoupis et al.*, 1996; *Cosgrove and Tsunoda*, 2002; *Hysell et al.*, 2004], and analysis of data from incoherent scatter radars [e.g., *Heinselman and Nicolls*, 2008; *Nicolls et al.*, 2014], to name a few.

A weaker form of electrostatic assumption involves substitution of electrostatic waves for electromagnetic waves, and assuming they are essentially equivalent. In this case the time-derivative is dropped in Faraday's law (so that $\vec{\nabla} \times \vec{E} = 0$, and Maxwell's equations become the Poisson equation), but retained in at least some of the remaining equations, so that waves are still supported. In ionospheric physics this "electrostatic-wave theory" is used to analyze plasma instabilities, and to compute the spectrum of backscatter for incoherent scatter radar. The validity of electrostatic-wave theory is a distinct question from that of the full electrostatic theory, which does not include wavelike effects. We also analyze this weaker assumption, by substituting the Poisson equation for the Maxwell equations. We find that electrostatic waves may be good substitutes below about 100 m in transverse wavelength, but above 100 m they have different properties that would

produce very different results in the TL theory calculation. In the case of magnetohydrodynamic (MHD) waves, which are non-collisional, a referee has stated that this is a known result, which follows from a more general result that Alfvén waves become electrostatic when the transverse scale becomes less than the electron inertial length [however see *Saleem*, 2001]. But we are not aware of equivalent results for the fully collisional ionosphere, and it is often suggested that the high collision frequencies preclude electromagnetic effects.

Focus on Ionospheric Conductance: Although the TL method includes calculation of the local field quantities like electric and magnetic field, we focus discussion on the transverse-scale-size dependence of the "ionospheric conductance" [e.g., *Fuller-Rowell and Evans*, 1987; *Lysak*, 1990], which is a linearized and non-local quantity the non-locality of which is normally handled with electrostatic theory, through the electric field mapping assumption. As such, the ionospheric conductance provides an appropriate differentiator for testing electrostatic theory, such as the scale-size-dependent electric-field mapping it entails. In addition, MHD and other models of the magnetosphere generally employ an inner boundary condition that is established by considering the ionosphere as an electrical load, characterized by this ionospheric conductance, which is meant to represent the input admittance seen from above the ionosphere [e.g., *Raeder et al.*, 1998; *Gombosi et al.*, 2001; *Toffoletto et al.*, 2003; *Lyon et al.*, 2004; *Janhunen et al.*, 2012; *Lotko et al.*, 2014]. This input admittance is therefore an important quantity for large-scale modeling of the Sun-Earth interaction. Under electrostatic theory it is derived by integrating the (zero frequency) ionospheric conductivity along the geomagnetic field, based on the mapping assumption. Dropping the assumption that it is purely real, and dropping the assumption that it can be derived from electrostatic theory, we arrive at an important problem that is well suited to TL theory, that of deriving the ionospheric input admittance.

In doing this we want to emphasize that we are doing it as a function of the transverse wavelength of the imposing signal, in order to make comparisons with electrostatic theory as it is commonly used in the ionosphere. The most comparable previous results of which we are aware are those found by *Knudsen et al.* [1992], which do support the calculation of conductance as the field line integrated conductivity, for sufficiently low-frequencies. However, for reasons that are discussed in Section 2, and more fully in *Cosgrove* [2016], we believe that these results must be subordinated to the results presented herein. There are also a number of time domain simulations that might seem applicable [e.g., *Seyler*, 1990; *Birk and Otto*, 1996; *Zhu et al.*, 2001; *Otto and Zhu*, 2003; *Dao et al.*, 2013; *Lysak et al.*, 2013; *Tu and Song*, 2016; *Tu and Song*, 2019]. But we have not been able to find where any of these actually compute the ionospheric conductance as a function of the transverse wavelength. Some likely reasons for this are discussed in the background section (2), although with humility as we are not in a position to properly critique the full collection of complex and sophisticated modeling efforts. The summary is that these time-domain simulations are addressed primarily to complex transient phenomena on global scales, and are not optimized for calculating the wavelength dependent conductance, which is only accessible to them through implementation of potentially impractical boundary conditions, and then by running the model for a long time, until everything stops changing (steady state).

What appears to be true is that the calculation provided in the present article represents the most rigorous attempt yet to calculate the ionospheric conductance as a function of the transverse wavelength. We give an exact linearization of the electromagnetic 5-moment fluid equations without making any of the usual "resistive MHD" or other simplifications. Some such simplifications are made in every other work we have found, and we do not believe there is any way to know if they might be affecting the conductance found (or which would be found) for the highly collisional E region ionosphere, except by removing the simplifications as we have done in this work. The approximations that we do make are all ones that are clearly beneficial to the prospects for recovering electrostatic theory at long transverse wavelengths (or low frequencies), with the only possible exception being use of the usual understanding of wavepacket propagation, which we are mostly able to validate. We also note that our method involves a low level of numerical error, allows for continuous vertical resolution, is sanctioned by the principle of causality, and results in a much more analytical and physically intuitive description of electromagnetic ionospheric physics.

An additional motivation for this work comes from recent observational findings that the magnetosphere-ionosphere (MI) coupling interaction may actually occur over scales significantly shorter than previously expected. For example, using data from the Swarm satellites, *Pakhotin et al.* [2021] found that half the Poynting flux may be associated with events having transverse-scale-size less than 250 km. If this is true then having an accurate transverse-wavelength-dependent characterization of ionospheric conductance is in

fact very important for large scale modeling, and so it is important to either validate or replace the current electrostatic paradigm for conductance.

Contents and Barrier to Entry: Due to the many years that electrostatic theory has been used in ionospheric physics, and the many papers that have been written on waves in the magnetosphere, mostly using other techniques, we anticipate the need to prove the veracity of our calculation. At the same time, we anticipate the need for a relatively simple example that illustrates the breakdown of electrostatic theory and motivates the calculation.

To meet the first need, in Sections 4 and 5 we derive our generalized form of TL theory from an exact solution to the full equations of motion for the linearized problem, while describing every approximation in detail. We also review why linearization is appropriate. Then in Section 6 we use the results of Section 4 to give a (brief) quantitative presentation of the properties of the wave modes that are relevant to ionospheric physics, and to compare the electrostatic and electromagnetic waves with respect to the critical modeling parameters of parallel wavelength and admittance.

To meet the need for a simple example, in Section 3 we reframe selected results from *Cosgrove* [2016] in the form of a gedanken experiment that illustrates the breakdown of electrostatic theory and introduces TL theory. This discussion is continued in Section 6.2 where the quantitative results of Section 6 are added, in order to discuss the likely physical behavior of the ionosphere, and to justify retaining only two modes. Together these sections provide a framework for understanding the major results, and we refer back to them often in the final presentation of results in Section 7.

The following background section is for readers seeking a more thorough introduction. Other readers may wish to skip to Section 3.

2 Background

TL Theory: TL theory uses the Laplace transform to derive the response of a uniform transmission line to a harmonic source at its input that turns on, and then continues operating indefinitely [e.g., *Nilsson*, 1984; *Miano and Maffucci*, 2001]. The Fourier transform is applied in the spatial domain so that the steady-state part of the linearized response can be characterized by quantities such as wave-number and wave-admittance, where usually only one wave mode is retained. Then, finite length sections of transmission line having different characteristics (e.g., different admittances) are pieced together, such that each contains a superposition of oppositely-directed pieces of its unique steady-state response, and suitable boundary conditions are enforced across the junctions. The oppositely directed pieces can be thought of as incident and reflected waves, that is, for the case when one of the ends is not connected to a source. This formalism allows for deriving the steady-state response of an assemblage of these lines, which can include branches, loop backs, or whatever. An amazing variety of real, three-dimensional circuits have been approximated in this way [e.g., *Matthaei et al.*, 1980], using specialized software packages such as Touchstone [*EEsoft*].

Note that although the term transmission "line" is used, the formalism can be applied to a slab-like geometry to produce a 3D model for a signal incident on a vertically inhomogeneous ionosphere, where the transverse wavevector arises as an independent parameter. Note also that although the formalism sounds inherently approximate, we can derive it from an exact solution, and the approximations can be well understood. Thus, the idea of extending these methods to the ionosphere is attractive, and one could imagine that software similar to Touchstone could be made available for ionospheric modeling.

Difficulties for Temporal/Spatial-Domain Models: TL theory has strengths and weaknesses that are complementary to those of the nonlinear models that are more commonly used in the ionosphere and magnetosphere, which resolve the system in time and space. To begin with, note that circuit theory in electrical engineering is essentially a theory of linear systems in steady state, where these expedients allow for a source-independent characterization of the system. If we wish to deploy the concepts of admittance and conductance to ionospheric science then we must adopt a linear and steady-state description. There are two major aspects of temporal/spacial-domain models that make this a difficult proposition: first, the source itself must be unvarying; and second, the model must be run for a long time, until everything stops changing. We consider these two difficulties in turn.

What is meant by the the source being unvarying is that the source should produce an unchanging superposition of waves incident on the system. Ideally there should be only a single wave, which can be

varied so as to fill out the Fourier spectrum. A single wave incident on the system is characterized by its wave vector, polarization vector, frequency, phase, and sufficiently-small amplitude. This creates a difficulty for spatial domain models because in such models the source usually takes the form of a boundary condition imposed at the input to the system. The system reflects some of the incident waves, such that the needed boundary condition is the sum of the incident and reflected waves. But since the goal is to characterize the system, it follows that the system has not yet been characterized, and so there is no way to predict the reflected waves from the incident waves. There is no way to know, a priori, the correct boundary condition corresponding to a particular, chosen, incident wave. And an arbitrarily chosen boundary condition may not reflect any such case, it likely reflects a source producing a *changing* superposition of incident waves, which defeats the steady-state characterization.

One way around this problem is to extend the modeled domain so that the source is a long way from the region of interest, so that the system can be characterized before the reflected wave returns to corrupt the response [e.g., *Streltsov and Lotko*, 2003]. However, to find the steady-state response from a time-domain model it is necessary to run the model for a long time, until everything stops changing. Hence the system must come to steady-state before the reflected wave returns, which does not seem very practical.

In addition, if the model contains nonlinearities then the steady-state will depend on the amplitude of the excitation, and it may well be that it never reaches a steady state. In either of these cases the model is not applicable to calculating the admittance, unless it is modified by removing the non-linearities. And what is the point of a numerical simulation of linear wave propagation when an analytical solution is available? A numerical simulation involves errors that build up over time, and it is very difficult to analyze these errors, especially in the limit as time goes to infinity.

Advantages of TL Theory: By contrast, TL theory does not encounter any of these difficulties, because it utilizes the analytical, steady-state solution that is available once the system is expressed in the Laplace/Fourier domain, and linearized. And this has the additional advantage of providing a result that is easy to interpret physically: the solution is expressed as a sum over steady-state wave-modes in analytical form, plus a background state. Thus TL theory is both intuitive and natively addressed to calculating circuit quantities, using the assumptions of linearity and steady-state that are crucial elements in their definition.

TL theory also has the advantage that the scale-size-dependence of the ionospheric conductance can be calculated using a model that is effectively one-dimensional; that is, the background state can be one dimensional, while the incident signal is represented in the Fourier domain (so that it is three-dimensional). This is in contrast to the situation for a nonlinear model where one-dimensional means that all transverse derivatives vanish, including those for the incident signal, so that there is no field-aligned current [e.g., *Tu et al.*, 2014]. In order to address current-closure through the ionosphere, and the scale size dependence of electric field mapping, non-linear models must be at least two-dimensional, and this creates a much heavier numerical burden.

Another advantage of TL theory is the ability to explicitly remove the parasitic contributions from high frequency modes without any distortion of the retained modes, which arises from working in the Laplace/Fourier domain. In the time-domain, finite-difference approach it is necessary either to use a time-step short enough to resolve the radio-frequency waves (which is generally impractical), or to introduce low-frequency approximations to the governing equations that kill these modes. The low-frequency approximations have some effect on the retained modes, and may cause distortion in the E-region ionosphere, where the collision frequency is very high. Since the E-region contains most of the ionospheric conductivity, any compromise with respect to collision frequency introduces uncertainty into calculation of the ionospheric conductance.

Another advantage of TL theory over spatial-domain models is the ability to achieve continuous spatial-resolution. Spatial domain models cannot find Fourier components above the Nyquist frequency for the spatial sampling, although below this frequency there is continuous resolution of the Fourier components. This situation is reversed for Fourier-domain models: Fourier domain models cannot model locations farther away than a certain distance from the center of the spatial domain, but within this region there is continuous spatial resolution. This dichotomy means that while spatial-domain models may be more useful for large-scale modeling, they will have quite a bit of difficulty resolving the sharp features that we find below are critical to the ionospheric conductance. Hence it is important that both kinds of models are developed and used in tandem.

Boundary Value Methods: There is also another kind of solution for the equations of motion that

we should mention, which is often used to study ELF/VLF waves in the ionosphere. Instead of the Laplace transform, the Fourier transform is applied to the time axis and either the ionospheric Ohms law or cold-plasma dielectric tensor are incorporated into the Maxwell equations, in lieu of the plasma momentum equations [e.g., *Hughes*, 1974; *Knudsen et al.*, 1992; *Kuzichev et al.*, 2018]. Application of the Fourier transform to the time axis was discussed in Section 7 of *Cosgrove* [2016], where it was explained that it produces a boundary value problem. This approach does not apply the equations of motion to the Cauchy problem for which they are sanctioned by causality. The dispersion relation for the equations of motion is solved for complex $\vec{k}$ as a function of a real-valued ω, whereas for the Cauchy problem the (same) dispersion relation is solved for complex ω as a function of real $\vec{k}$. These are two different operating points on the dispersion surface and so almost-certainly the solutions will be different. If different, only one of them can be physical.

This relationship was studied by *Cosgrove* [2016], and a number of concrete differences were found. We mention two of them that are particularly simple to understand. First, the parallel wavelengths corresponding to the same frequency (real part of ω) do not agree with each other. And second, the conductivity equations are evaluated either with, or without an imaginary component in ω (and we note that the conductivity equations do not depend on $\vec{k}$). In Figure 16 of *Cosgrove* [2016] the Pedersen conductivities evaluated in these two ways were compared, and at least for the near-DC frequencies of interest in this work, it was found that the imaginary part of ω is very important. And, the imaginary part of ω also depends on the mode of propagation.

Thus, in using the Ohms-law/dielectric-tensor in Maxwell's equations without the imaginary part of frequency there is an effect of "squeezing together" the modes under a single, inaccurate conductivity, in order to accommodate the smaller equation set (i.e., momentum equations omitted). Coupling this with the parallel wavelengths being different, it appears that the boundary-value methods will produce a solution very different from the Cauchy-problem methods used below. In the discussion of equations (17) and (18) in *Cosgrove* [2016], it was shown that the boundary value method might lead to a solution very close to electrostatic equilibrium. But based on the fundamental principle of causality in physics, the solution of the Cauchy problem must be considered as the physical one. In what follows we use TL theory to solve the Cauchy problem for the steady-state solution.

Generalizing the TL Theory: The advantages explained in the previous paragraphs allow this work to achieve its goal of an electromagnetic calculation of the scale-size dependence of the ionospheric conductance, and of the associated electric field mapping and current closure through the ionosphere, etc. Temporal/Spatial-domain models are not really appropriate for this purpose, for all the reasons expressed above, and this may explain why such a calculation has never been done, to our knowledge.

However, TL models in electrical engineering normally involve a single wave mode, and circuits are generally designed to ensure that only one mode is supported. In the ionosphere we do not have this luxury, and so we will generalize the usual TL theory to one that includes multiple modes. Doing this means that the scalar admittance, which relates the electric and magnetic fields, must be replaced by a tensor such that there is an "admittance vector" or "polarization vector" for each mode. A problem arises in that the boundary conditions connecting different sections must be expanded, and we are not aware of any universal rules that can be applied. However, we find that as long as consideration is limited to scales above a few tens of meters, the ionosphere supports only two relevant modes, which can be roughly associated with the usual, physically-defined Alfvén and Whistler waves. In this case there are sufficient criteria available for setting the boundary conditions, and we are able to proceed with a bi-modal form of TL theory.

Another added complexity arises from the equations of motion, which must describe the evolution of a collisional plasma, and therefore must be extended beyond Maxwell's equations. In earlier work [*Cosgrove*, 2016] we discussed waves in the ionosphere (a collisional plasma), and showed how they can be derived from the combined Maxwell and plasma momentum equations without simplifications. The methods employed were actually quite general, and in this work we extend them to the 5-moment fluid equations [*Schunk and Nagy*, 2009], which will form the equations of motion for this work. The waves that inhabit our transmission lines are the waves of the electromagnetic 5-moment fluid equations, formally defined through eigenvectors and eigenvalues, and validated by numerical evaluation of the exact integral expressions. We have also tested the model using the smaller equation-set from *Cosgrove* [2016], and the results appear to be exactly the same. Using the larger equation-set allows for evaluating possible contributions from lower-frequency waves, and for possibly including some of these waves in later editions.

3 Gedankenexperiment and Breakdown of Electrostatic Theory

To motivate the construction of an electromagnetic model it is useful to analyze a toy example that illustrates the limitations of electrostatic theory as it pertains to the ionosphere. Consider the case of the horizontally extended, vertically thin E-region dynamo shown in panel a of Figure 1, which is driven by a horizontal neutral wind, and embedded at the bottom a semi-infinite homogeneous plasma with vertical geomagnetic field (and no wind). Such a vertically localized source excites the characteristic modes at the surface of the surrounding plasma, at the frequency and wavelength of the source, which we imagine to be a pure tone. Those that are able to propagate at that frequency and wavelength carry energy away from the source, while gradually dissipating [*Bernstein*, 1958]. Those that cannot propagate will produce a near field that we will ignore, or, what amounts to the same thing, we will consider the upper boundary of the source to be beyond the near field. As time goes to infinity a steady state condition arises where the plasma oscillates harmonically with the source. The time scale for reaching steady-state is generally thought very short compared to other relevant ionospheric time scales, and this is a key assumption of electrostatic theory [*Farley*, 1959], which also applies to TL theory. Nonlinear interactions of the waves may direct the evolution away from any kind of steady state, and also introduce a non-trivial dependence on the amplitude of the source. Thus this kind of a description assumes signal amplitudes are small enough that nonlinearities can be ignored, and applies to the far zone (aka, radiation zone) of the source after the system has reached steady state.

In order to apply the standard TL theory, consider the hypothetical case where there is only one propagating mode, and calculate the input admittance seen by the dynamo of panel a, Figure 1, as it looks up at the uniform plasma above it. This is the admittance that loads the dynamo, affecting the net electric field and current (and etc.) that are produced, in accordance with the dynamo's own internal admittance. We can calculate the input admittance using both electrostatic theory and TL theory, which in this case is a single mode version of electromagnetic theory. Most of this material was covered by [*Cosgrove*, 2016], although here we use standard transmission line formulas instead of deriving the results directly, and organize the material in a way that might serve to make it more accessible.

Electrostatic Calculation: For the electrostatic calculation, take the electric field at the surface of the dynamo to have the form $\vec{E} = \left(\hat{y}\tilde{E}_y + \hat{z}\tilde{E}_z\right) \mathrm{e}^{i(\omega t + k_z y + k_z z)}$, where $\hat{y}$ is transverse to the geomagnetic field (tangent to the boundary), and $\hat{z}$ is along the geomagnetic field. There is no $\hat{x}$ component because the curl of the electric field must be zero under electrostatic theory, and we assume the convention that the wavevector is perpendicular to $\hat{x}$. Under electrostatic theory, as illustrated in the panel b of Figure 1 (Case I), the transverse electric field at the surface of the dynamo $\left(\vec{E}_\perp = \hat{y}\tilde{E}_y \mathrm{e}^{i(\omega t + k_y y)}\right)$ maps a distance d_F along $\vec{B}_0$ into the homogeneous plasma, and therefore drives a field-line-integrated transverse current, $\vec{J}_\perp = \vec{E}_\perp \sigma_{P0} d_F - \vec{E}_\perp \times \hat{z}\sigma_{H0} d_F$, where σ_{P0} and σ_{H0} are the zero-frequency Pedersen and Hall conductivities, respectively. The distance d_F is the Farley mapping distance, which is the distance an electric field is expected to map along the geomagnetic field under electrostatic theory (see Section 1). Beyond the distance d_F there is no electric field, and thus no current. Therefore, by current continuity, the region of homogeneous plasma returns a current density obeying $j_\parallel = \vec{\nabla}\cdot\vec{J}_\perp = \sigma_{P0} d_F \vec{\nabla}\cdot\vec{E}_\perp$, where $j_\parallel$ is the current density into the adjoining plasma (parallel to the geomagnetic field). That is, under electrostatic theory, the adjacent homogeneous plasma presents a load to the dynamo-source that can be characterized by the input admittance,

$$Y_{in\mathrm{Matched}} = \frac{j_\parallel}{\vec{\nabla}\cdot\vec{E}_\perp} = \sigma_{P0} d_F. \tag{1}$$

If instead the region of homogeneous plasma ends a short distance from the dynamo layer, such that the conductivity beyond is zero, then Case I of panel b is replaced by Case II. Under electrostatic theory, the electric field maps across the thin region of uniform plasma, and beyond there is no current. Thus, the thickness L of the region of homogeneous plasma replaces d_F in the expressions of the previous paragraph, and the input admittance becomes,

$$Y_{in\mathrm{OC}} = \frac{j_\parallel}{\vec{\nabla}\cdot\vec{E}_\perp} = \sigma_{P0} L, \tag{2}$$

where "OC" stands for "open circuit."

Electromagnetic Calculation: Now consider calculating the input admittance using the (single-mode) TL theory. When the dynamo is bounded by a semi-infinite homogeneous plasma, as in panel a, there can

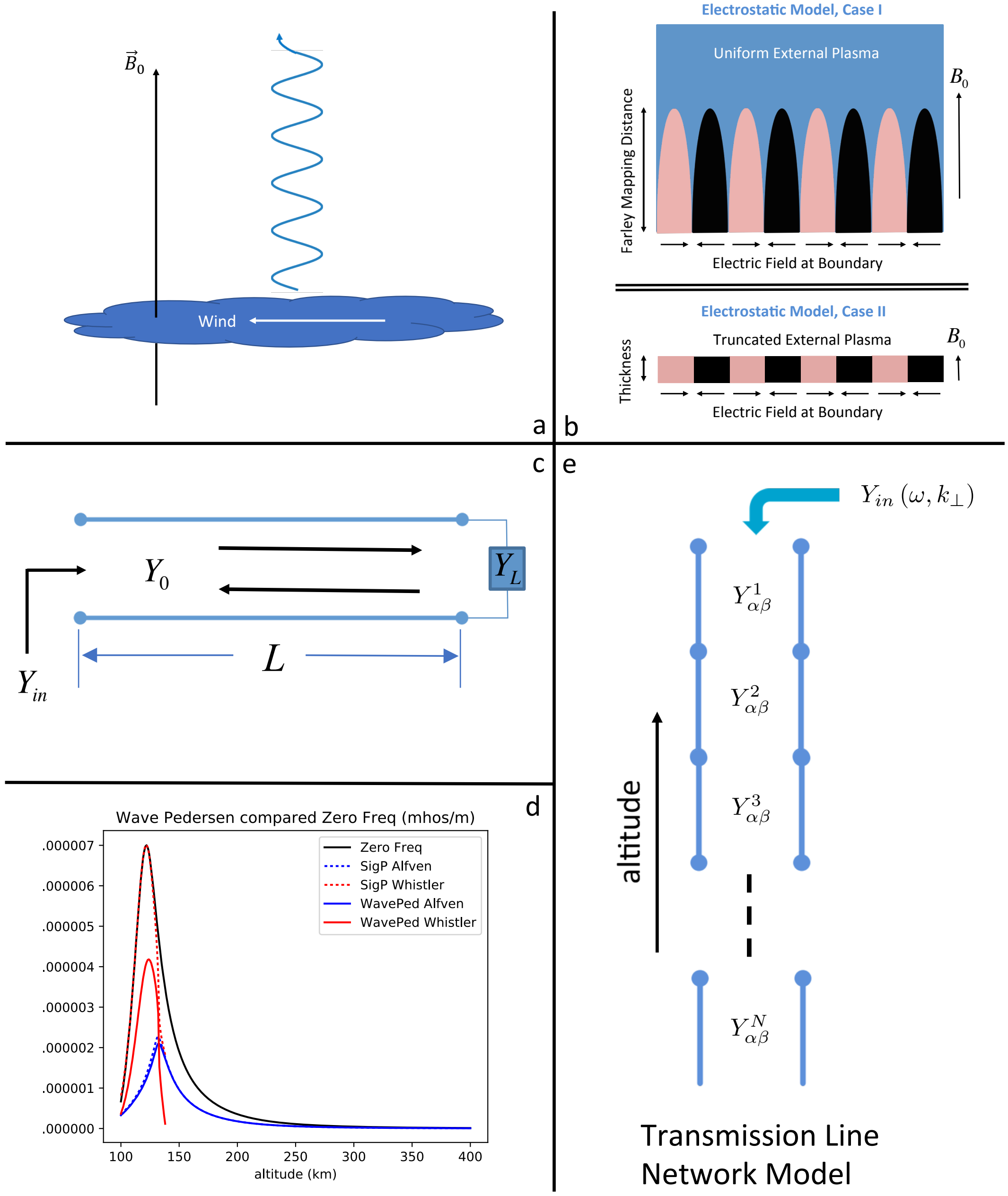

Figure 1: Electrostatic versus TL theory: panel a, schematic for the gedanken experiment; panel b, electrostatic approach; panel c, TL terminated by load admittance Y_L; panel d, wave-Pedersen conductivity compared with the standard zero-frequency Pedersen conductivity, and the full Pedersen conductivity, for the Alfvén and Whistler modes ($\lambda_\perp = 1000\,\text{km}, n_e = 4.7 \times 10^9 \text{m}^{-3}$); panel e, ionosphere modeled as a cascade of short transmission line sections. Each section represents a thin slab of homogeneous plasma.

be no back reflection, and so there is only a single wave propagating away from the dynamo. So to obtain $Y_{in\mathrm{Matched}}$ under TL theory we need to compute the quantity $j_{\|}/\vec{\nabla}\cdot\vec{E}_{\perp}$ for a single propagating wave, which we can do from the results in *Cosgrove* [2016] (or using our results below). However, we do not actually need specific values to make our major point.

The input admittance associated with a single wave propagating away is commonly referred to as the characteristic admittance of the wave, Y_0. It is this quantity that enters into the well-known TL expression for the input admittance to a transmission line terminated by a load admittance Y_L [e.g., *Collin*, 1966],

$$Y_{in} = Y_0 \frac{iY_0 \tan\phi + Y_L}{iY_L \tan\phi + Y_0}, \tag{3}$$

where the electrical length $\phi = k_z L$ is the phase rotation along the transmission line, k_z is the parallel wavevector, and L is the line length. More precisely, the formula (3) actually allows ϕ to be complex, and is thus slightly generalized from what can be found in textbooks like *Collin* [1966], where dissipation is not included. Panel c of Figure 1 illustrates the application for the formula. The arrows in the figure illustrate how the formula is derived, as the superposition of two waves traveling in opposite directions, related by the reflection coefficient at the load, $(Y_0 - Y_L)/(Y_0 + Y_L)$. Note the assumption that the waves have finished traversing the line and come to steady state. Note, also, the assumption that the waves have small enough amplitudes that non-linear interactions can be ignored, so that linear superposition is valid. These assumptions underlie the whole of circuit theory in electrical engineering, and the definition of admittance depends on them.

Using equation (3), the result for the semi-infinite case is found by matching the load to the characteristic admittance, so there is no back-reflected wave, that is, $Y_L = Y_0$. Substituting into equation (3) gives,

$$Y_{in\mathrm{Matched}} = Y_0, \tag{4}$$

which, of course, just shows that the formula is working as intended; we have defined Y_0 to be the input admittance when the load is matched to the line, that is, when there is only a single wave propagating away. Note that this result recalls the formula $j_{\|}/\vec{\nabla}\cdot\vec{E}_{\perp} = (\mu_0 V_A)^{-1}$ that is used in classical magnetosphere-ionosphere coupling papers, such as [*Mallinckrodt and Carlson*, 1978, in Gaussian units], where $(\mu_0 V_A)^{-1}$ is the characteristic admittance of the (collisionless) Alfvén wave, and V_A is the (collisionless) Alfvén velocity. Note also that if the displacement current is included in $j_{\|}$, our definition for characteristic admittance encompasses the definition in terms of electric and magnetic fields that is commonly used in free space (the displacement current is thought negligible in the ionosphere [*Song and Lysak*, 2006]).

Now consider the second case, when the region of homogenous plasma is bounded, instead of being semi-infinite. In this case there will be a back-reflected wave, and we actually need equation (3). When the homogeneous plasma has an upper boundary the current above the boundary must be zero, that is, $Y_L = 0$. Making this substitution into equation (3) gives the electromagnetic prediction for the input admittance,

$$Y_{in\mathrm{OC}} = iY_0 \tan\phi. \tag{5}$$

Comparison of Results: Assuming only that the waves are relatively low loss, so that ϕ is mostly real, we can see that the electrostatic results are not consistent with the electromagnetic (TL theory) results. The electrostatic expressions (1) and (2) are both purely real-valued, whereas one of the electromagnetic expressions has an i in front. This dichotomy is well known in TL theory, where a matched line generally looks like a resistor, while a short open-circuited line generally looks like a capacitor. The electrostatic results do not capture this dichotomy, which is solidly established in TL theory. This is what we mean by the "breakdown" of electrostatic theory.

However, in the usual case for TL-theory Y_0 is a real number, which makes $Y_{in\mathrm{OC}}$ imaginary (a capacitor). For the ionospheric case we have found, both in this work (panels g and h of Figure 2) and in *Cosgrove* [2016], that Y_0 is strongly imaginary, which makes $Y_{in\mathrm{OC}}$ a real number. This makes sense because it allows the electrostatic and electromagnetic results to agree (with respect to real/imaginary, anyway) when the system size is small. This is what we expect; if electrostatic theory is to apply, it should apply when the system size is small compared to a wavelength. When we go to calculate the input admittance to the ionosphere, we will essentially be calculating $Y_{in\mathrm{OC}}$, where L is the thickness of the ionosphere. $Y_{in\mathrm{OC}}$ being a real number

allows for the possibility of salvaging the usual view that the input admittance to the ionosphere should be the field line integrated conductivity, a real number.

So how well do the electrostatic and electromagnetic results agree for $Y_{in\mathrm{OC}}$? Equations (5) and (2) have a different functional dependence on L, and cannot agree in general. The tangent factor in equation (5) describes well-known wave effects on admittance: when moving along an open-circuited transmission line (or as the line gets longer) the input admittance goes through infinities that alternate in sign, which occur where the electric field of the reflected wave cancels that of the incident wave. Since $Y_{in\mathrm{OC}}$ is usually imaginary, these are infinite reactances that alternate between being capacitive and being inductive. But for the ionosphere $Y_{in\mathrm{OC}}$ is a real number. So these would be infinities in the ionospheric conductance, which could become negative if the electrical thickness (ϕ) of the ionosphere becomes greater than 90°. This result is a harbinger of results from the model developed below. Note that an infinity in the admittance simply corresponds to a zero in the electric field, and so is not a cause for alarm.

The electrostatic and electromagnetic expressions for $Y_{in\mathrm{OC}}$ can be compared in the limiting case where the thickness L of the plasma slab is much less than a wavelength. Substituting for $\phi = k_z L$ in equation (5) and letting the thickness L become small, we obtain $Y_{in} = iY_0 k_z L$. This expression has the same L-dependence as equation (2), and so we can compare the L-coeficients, $iY_0 k_z$ and σ_{P0}. Making this same comparison, *Cosgrove* [2016] suggested defining a wave-Pedersen conductivity (their equation (14)), which in our current analysis takes the form,

$$\sigma_{Pwj} = iY_{0j} k_{jz}, \tag{6}$$

and we have added subscripts j to denote different wave modes. In this gedanken experiment we are considering only one mode, but we find below that there are in fact two important modes. The wave-Pedersen conductivity is distinct from the local Pedersen conductivity, which, also as explained in *Cosgrove* [2016], can either be calculated under the assumption that both the real and imaginary parts of frequency (ω) are zero (σ_{P0}, the usual electrostatic Pedersen conductivity), or using the full complex ω (what we call the "full Pedersen conductivity"), where the imaginary part of ω was shown to be very important.

Cosgrove [2016] explained that σ_{Pwj} and σ_{P0} cannot agree in general, because σ_{Pwj} depends on wavelength and σ_{P0} does not. The full Pedersen conductivity does have a wavelength dependence, which arises through its dependence on ω. It agrees much better with σ_{Pwj}, and this shows the importance of the imaginary part of ω in the conductivity equations (see Figure 16[1] from *Cosgrove*, 2016). Panel d of Figure 1 shows the results from an example calculation of σ_{Pwj} for the Alfvén and Whistler waves, using a 1000 km transverse wavelength and plasma density of $4.7 \times 10^9 \mathrm{m}^{-3}$. For comparison, both the zero-frequency and full Pedersen conductivities are shown, where in the latter case there is one for each wave type. In the case of the Alfvén wave, the plot shows fairly good agreement between σ_{Pwj} and the full Pedersen conductivity. In the case of the Whistler wave, the plot shows agreement between σ_{P0} and the full Pedersen conductivity in the low altitude region. This is because the Whistler wave has a long dissipation-time-scale in the low altitude region (i.e., imaginary part of frequency is small). But σ_{Pwj} does not agree with σ_{P0} for either wave, and this is the result that is relevant to our discussion.

The real/imaginary inconsistency noted above can be avoided if the electrical length ϕ is strongly imaginary. In the usual case for TL theory the dissipation scale length is much longer than the wavelength, and is ignored. But if it were not ignorable, the electrical length would acquire an imaginary component, $\phi = k_z L = 2\pi L/\lambda_z - iL/l_{dz}$, where l_{dz} is the dissipation scale length and λ_z is the wavelength. Note that including this loss term "cures" the infinities noted earlier, because the reflected wave can never completely cancel the electric field of the incident wave. If it were to be the case that both l_{dz} is much less than λ_z, and that the characteristic admittance is strongly real, then both $Y_{in\mathrm{OC}}$ and $Y_{in\mathrm{Matched}}$ would be strongly real, as predicted by electrostatic theory. Physically, this would be the case where the "waves" are so lossy that they do not really behave like waves. This special situation essentially allows the system size requirements to be removed.

Criteria for Electrostatic Theory: In summary, we have shown that when electromagnetic theory can be approximated using a single wave mode, then electrostatic theory is also applicable when the following three conditions hold: (1) the wave-Pedersen conductivity (σ_{Pwj}) agrees with the zero-frequency Pedersen conductivity (σ_{P0}); (2) the wavelength is long compared to the system size; and (3) the dissipation scale length is long compared to the system size. There is also a special case where if the dissipation scale

[1] Errata: In the caption for Figure 16 of *Cosgrove* [2016], "equation (1)" should be "equation (A9)."

length is much less than the wavelength, and is also equal to the Farley mapping distance, then requirements (2) and (3) may be replaced with the requirement that the dissipation scale length is short compared to the system size (which essentially removes the system size requirements).

However, both from the results below and from the results in *Cosgrove* [2016], we find that these conditions do not hold; the wave-Pedersen conductivity does not equal the zero-frequency Pedersen conductivity, the dissipation scale length is much longer than the wavelength (for both modes), and the wavelength is not sufficiently long compared to the ionospheric thickness. And when multiple modes are present there are additional unlikely requirements that must be added, which we touch on briefly in Section 7.1. Fortunately, the characteristic admittance is strongly imaginary, and so at least the ionospheric input admittance is strongly real.

In Section S.3 of the Supplementary Information we provide some additional commentary on energy conservation in this unusual situation. In sorting all this out it is important to observe that wave dissipation is not a critical or necessary pathway for energy absorbed by the system. Rather, the critical pathway is energy carried away by waves, converted to/from some other form (like kinetic energy [*Vasyliunas and Song*, 2005; *Cosgrove*, 2016]), or dissipated by nonlinear effects. If the wave amplitudes are sufficiently small, the nonlinear effects may be ignored in their computation. As long as the wave amplitudes are accurate, the Poynting flux computed from the superposed waves is valid, and the heating may also be calculated. And when there *is* significant wave dissipation the signal is absorbed before reaching the bottom of the ionosphere, which means, among other things, that the electric field does not map through the ionosphere.

The discussion above is essentially a restatement of results from *Cosgrove* [2016], which although largely denying electrostatic theory in the ionosphere, did not provide a practical replacement. In this work we put the ideas into action and perform an electromagnetic calculation that forms the basis for a deployable electromagnetic model, which can replace electrostatic theory in many cases, and which provides for a significant amount of physical intuition.

4 Solution of the Equations of Motion, and Model Building Blocks

We adopt the electromagnetic 5-moment fluid equations [e.g., *Schunk and Nagy*, 2009] for description of collisional plasma, such as the ionosphere. These well known equations of motion are presented in Appendix A (A.1), along with the derivation of the matrix H_5 (Figure A.1) used below, which contains their linear parts. In addition to the Maxwell equations, the equations of motion (A.1) include the continuity, momentum, and energy equations for electrons and one species of ion. Sources such as wind, background electric field, photo-electrons, and gravity are set to zero or omitted, except in the case of the explicit external source we add to derive the driven steady-state solution. Hence, the equations of motion expand on those used in *Cosgrove* [2016] by adding the continuity and energy equations. They are appropriate for exploring the relationship between electrostatic and electromagnetic theories of the ionosphere for scale-sizes greater than a few tens of meters, but they do not include kinetic effects, the dynamics of the neutral gas, or interactions between different species of ion (although the latter two categories could, in principle, be added to the analysis with little change in method). Additional details can be found in Appendix A. Here we focus on deriving the linearized, driven steady-state solution using an abstract matrix notation. The methods employed are standard textbook methods, and our contribution is only to apply them to this problem.

Taking the Fourier transform in space, the equations of motion (A.1) can be written,

$$\frac{\partial \vec{X}}{\partial t} - iH_5\vec{X} = \vec{F}(t), \tag{7}$$

where $\vec{X}$ is a vector containing the deviations from thermal equilibrium of the 16 dependent variables, H_5 is a time-independent 16×16 matrix containing the linear parts of the equations, and $\vec{F}(t)$ is a time-dependent length-16 vector containing the nonlinear terms. The variables in $\vec{X}$ are defined in Appendix A, and consist of scaled versions of the densities, velocities, pressures, and electric and magnetic fields. The matrix H_5 is given in Figure A.1, and to be clear, is derived without making approximations (details are in Appendix A). The non-linear term $\vec{F}$ is not given, since we do not use it here.

The Intuitive Derivation of the Steady-State Solution: Before giving a (more) rigorous derivation of the model equations, we first motivate the results with a simple intuitive derivation based on the initial

value solution, which makes contact with the discussion in *Cosgrove* [2016]. We also remind the reader of the reasoning that makes it appropriate to ignore nonlinear terms.

To better understand the role of the nonlinear terms, consider diagonalizing the matrix H_5 as $\Omega = U^{-1}H_5U$, where U is the matrix having the eigenvectors of H_5 as columns, and Ω is diagonal. By applying U^{-1} from the left hand side, the equations of motion (7) breakup into individual scalar equations for each eigenmode, coupled only by nonlinear "source terms" on the right hand side:

$$\frac{\partial X'_j}{\partial t} - i\omega_j X'_j = F'_j(t), \tag{8}$$

where $\vec{X}' = U^{-1}\vec{X}$, $\vec{F}' = U^{-1}\vec{F}$, $\omega_j = \Omega_{jj}$, and j indexes the eigenmodes. Because the electromagnetic 5-moment fluid equations comprise 16 scalar equations, there are 16 eigenmodes, and equation (8) shows that the nonlinear terms have the effect of coupling energy from one mode to another (nonlinear leakage). As the size of the disturbance is reduced, there is (at least) a linear reduction of F'_j relative to $\frac{\partial X'_j}{\partial t}$ and $\omega_j X'_j$, and the rate of nonlinear leakage between modes is reduced. So for a sufficiently small disturbance the rate of nonlinear leakage is negligible, the eigenmodes evolve independently, and the general solution can be obtained. (As further described in Appendix A, we mean a small disturbance with respect to thermal equilibrium, and electrostatic theory is also formulated in this way.)

Therefore, consider the general homogeneous solution (in the language of differential equations) to equation (7), that is, the solution when the nonlinear "source" term, $\vec{F}$, is neglected, which is given by,[2]

$$\begin{aligned} \vec{X}\left(t,\vec{k}\right) &= \sum_{j=1}^{16} a_{0j}(\vec{k})\,\vec{h}_j(\vec{k})\,\mathrm{e}^{i\omega_j(\vec{k})t}, \text{ or, taking the inverse Fourier transform,} \\ \vec{X}\left(t,\vec{r}\right) &= \sum_{j=1}^{16} \int \mathrm{d}^3k\, a_{0j}(\vec{k})\,\vec{h}_j(\vec{k})\,\mathrm{e}^{i\left(\omega_j(\vec{k})t+\vec{k}\cdot\vec{r}\right)}, \end{aligned} \tag{9}$$

where $\left\{\vec{h}_j\right\}$ and $\{\omega_j\}$ are the eigenvectors and eigenvalues of H_5, respectively, $\vec{k}$ is the Fourier transform variable (the wavevector), and the $\{a_{0j}\}$ are 16 arbitrary coefficients for each $\vec{k}$. The solution is easily verified by direct substitution. Since H_5 is diagonalizable its eigenvectors are linearly independent, and so the solution (9) is the complete initial-value solution; an arbitrary initial state can be chosen by choosing the 16 $a_{0j}(\vec{k})$, and then evolution consists of 16 independently evolving eigenmodes.

Equation (9) shows that the eigenvalues of H_5 (i.e., the ω_j) play the role of complex frequency. Since equation (7) is the Fourier transform of real-valued equations of motion, the eigenvalues of iH_5 come in complex conjugate pairs. This means that when the real part of frequency ($\omega_{jr} = \mathrm{real}(\omega_j)$) is not zero there are paired two oppositely propagating eigenmodes (the complex conjugate negates ω_{jr}, reversing the direction of propagation), and hence there are 8 potential types of propagating eigenmode. For the smaller equation-set used in *Cosgrove* [2016] there were 6 potential types of propagating eigenmode. However, it is possible for ω_{jr} to be zero, for some j and range of $\vec{k}$, and in this range the eigenmode is evanescent; it is unpaired and does not effectively transmit energy, since the real part of its group velocity is zero.

To envision the source-free initial-value solution (9) we might imagine a narrow-band antenna with transmitter that turns on, transmits for a few cycles, and then turns off. To the extent the stimulus matches the natural energy-carrying modes of the plasma, energy will be transferred into the plasma, and carried away. To the extent it does not energy will not be able to transition into the plasma, only non-propagating modes will be excited, and the transmitter will feedback on itself in an effect analogous to a radio-frequency antenna that is not well matched to the free space impedance. By "non-propagating modes" we mean terms in equation (9) where the frequency and transverse wavelength of the antenna cannot be matched by the dispersion relation $\omega_j(\vec{k})$, and it is intended to imagine an unusually-large antenna that extends many wavelengths in the transverse direction, so that it will be transverse-wavelength selective.

[2]The general homogeneous solution (i.e., with $\vec{F} = 0$) to equation 7 is given by the matrix exponential, e^{iH_5t} [e.g., *Artin*, 1991]. However, as a practical matter, we find that H_5 is diagonalizable "almost everywhere," and this allows us to write the solution in a more explicit form, by taking advantage of the invertibility of the matrix of eigenvectors, U.

After the transmitter turns off, and at any significant distance, there will remain a localized disturbance consisting mainly of the modes that are able to propagate at the transmitter's frequency and transverse wavelength. Finding the $\{a_{0j}\}$ that fits the initial-value-solution to the disturbance, the integrals in (9) should produce a superposition of propagating wave-packets centered on wavevectors $\left\{\vec{k}_{0j}\right\}$ such that real $\left(\omega_j(\vec{k}_{0j})\right)$ matches the transmitter frequency, ω_0, where the transverse part of each $\vec{k}_{0j}$ matches the antenna wavelength.

Thus, using the standard interpretation of wave-packet propagation, we might imagine the subsequent evolution as a superposition of a few independently evolving wave packets centered on wavevectors $\vec{k}_{0j}$, propagating with group velocities $\vec{v}_{gjr} = -$ real $\left(\vec{\nabla}_k \omega_j\right)\Big|_{\vec{k}_{0j}}$, dissipating with time scales $\omega_{ji}^{-1} = 1/\text{imag}\left(\omega_j(\vec{k}_{0j})\right)$, oscillating with frequencies real $\left(\omega_j(\vec{k}_{0j})\right) = \omega_0$, and disturbing the plasma with polarization vectors $\vec{h}_j(\vec{k}_{0j})$. If the transmitter did this repeatedly, that is, if it did not turn off, there would result a steady-state disturbance around the source that decreases with distance according to the dissipation scale length $\vec{v}_{gjr}/\omega_{ji}$ (for each mode). This behavior amounts to, essentially, the steady-state solutions (14) and (B.3) that we now derive more rigorously, where the second of these applies to eigenmodes that become cutoff below some frequency (i.e., become non-propagating, i.e., not able to satisfy real $\left(\omega_j(\vec{k}_{0j})\right) = \omega_0$).

Validating the Steady-State Solution: The TL theory is based on the driven steady-state solution associated with a distant source transmitting into an infinite (and homogeneous) medium. Although the intuitive description just given seems almost adequate, there are in fact a number of assumptions that go into the wave packet interpretation, and so we would like to test it with a more rigorous derivation. We do this now, and for the most part find that the usual wave-packet interpretation appears to be a good one for our purposes. However, we do find some uncertainty with respect to the lower E-region ionosphere ($\sim$ 100 km altitude), where our method of validation is not fully applicable. This difficult problem is deferred to future work, and hence we regard our model as an important baseline associated with the usual wave packet interpretation, which is almost but not completely validated down to 100 km in altitude. In Section 7 we describe some sensitivity tests that show little effect from changes near the bottom of the modeling domain, and these reinforce the efforts at validation just below.

Therefore, consider the source,

$$\vec{f}_A\left(t, \vec{r}\right) = \vec{A}u(t)\mathrm{e}^{i\omega_0 t}\delta(z)\mathrm{e}^{ik_{0y}y}, \tag{10}$$

which turns on at $t = 0$ (u is the unit step function) and is localized in the z dimension, being non-zero only at $z = 0$ (δ is the Dirac delta function, and we assume that the geomagnetic field is along z). Taking the Laplace transform (with respect to time) of the left hand side of the general equation (7), and setting it equal to the Laplace and Fourier Transforms of the source (10), gives the following equation for the plasma response,

$$s\vec{X}\left(s, \vec{k}\right) - iH_5\vec{X}\left(s, \vec{k}\right) = \vec{X}(t = 0^-) + 4\pi^2\frac{\vec{A}}{s - i\omega_0}\delta\left(k_x\right)\delta\left(k_y - k_{0y}\right), \tag{11}$$

where s is the Laplace transform variable. This equation is solved for $\vec{X}\left(s, \vec{k}\right)$ by inverting the matrix $sI - iH_5 = U\left(sI - i\Omega\right)U^{-1}$, where $sI - i\Omega$ is diagonal. Then taking the inverse Laplace and Fourier transforms, and assuming zero for the initial condition $\left(\vec{X}(t = 0^-) = 0\right)$, we find the integral-form solution,

$$\begin{aligned}\vec{X}\left(t, \vec{r}\right) \;&=\; \mathrm{e}^{i(\omega_0 t + k_{0y}y)}\frac{i}{2\pi}\sum_{j=1}^{16}\int_{-\infty}^{\infty}\mathrm{d}k_z\frac{\vec{h}_j(0, k_{0y}, k_z)a_j(0, k_{0y}, k_z)}{\omega_j(0, k_{0y}, k_z) - \omega_0}\mathrm{e}^{ik_z z} \\ &-\; \mathrm{e}^{ik_{0y}y}\frac{i}{2\pi}\sum_{j=1}^{16}\int_{-\infty}^{\infty}\mathrm{d}k_z\frac{\vec{h}_j(0, k_{0y}, k_z)a_j(0, k_{0y}, k_z)}{\omega_j(0, k_{0y}, k_z) - \omega_0}\mathrm{e}^{i(\omega_j(0, k_{0y}, k_z)t + k_z z)},\end{aligned} \tag{12}$$

where $\vec{a} = U^{-1}\vec{A}$. Because imag$\left(\omega_j\right) > 0$, the second sum of integrals represents the transient response, which goes to zero as $t \to \infty$. Therefore, the driven steady-state solution is a sum over separate contributions from each eigenmode, according to the source vector $\vec{A}$, which are found by evaluating the integrals in the first sum of equation (12). We consider first an approximate analytical solution, and then test it by

numerical evaluation of the integrals for a few select cases. We will consider the case with $\omega_0 > 0$, which, comparing (12) with the exact initial value solution (9), means that there can be no contribution from modes with real$(\omega_j) \leqq 0$, and so we have at most 8 modes to consider.

The integrals in (12) involve eigenvalues, ω_j, and eigenvectors, $\vec{h}_j$, and it is well known that such quantities are not analytic over the entire complex plane. For example, eigenvalues are found as roots of a polynomial, and so expressions for them contain square and higher roots that are not analytic over the entire complex plane; analyticity fails where the argument of a square root vanishes, which is where two eigenvalues coincide [e.g., *Tsing et al.*, 1994]. So while it is tempting to consider analytic continuation off of the real-k_z axis, and use the residue theorem for the integrals, it will not do to simply take a residue where $\omega_j = \omega_0$. The dependence of ω_j on k_z is not analytic, and so the residue theorem does not apply (for example, see Figure 8 from *Cosgrove* [2016], which demonstrates non-analytic behavior). However, if we approximate the integrand along the real axis by a function that *is* analytic over the entire complex plane, then the residue theorem can be made to apply.

Because the integrand has denominator $\omega_j(0, k_{0y}, k_z) - \omega_0$, it should be peaked around the k_z having real$(\omega_j(0, k_{0y}, k_z)) = \omega_0$. Furthermore, the initial value solution (9) suggests that a plasma disturbance centered on wavenumber k_z should oscillate with frequency real$(\omega_j(0, k_{0y}, k_z))$, and the driven steady-state solution (12) oscillates purely at ω_0. Therefore, consider approximating the integrand by expanding around $k_z = k_{0jz}$ such that real$(\omega_j(0, k_{0y}, k_{0jz})) = \omega_0$. If we were to expand the denominator in a Taylor series and retain only the linear term, it would produce a simple pole at $k_z = k_{0jz} + i\omega_{ji}/v_{gj}$, where $\omega_{ji} = \text{imag}(\omega_j)|_{k_{0z}}$, and $v_{gj} = -\left.\partial\omega_j/\partial k_z\right|_{k_{0jz}}$. Therefore, consider approximating the integrand Γ by writing $\Gamma = \Psi e^{ik_z z}/(k_z - k_{0jz} - i\omega_{ji}/v_{gj})$ and seeking a linear approximation for Ψ in the vicinity of k_{0jz}. The factor $(k_z - k_{0jz} - i\omega_{ji}/v_{gj})/(\omega_j(0, k_{0y}, k_z) - \omega_0)$ is stationary at $k_z = k_{0jz}$ (with value $-1/v_{gj}$), and so the only first order variation of Ψ comes from the factor $\vec{h}_j(0, k_{0y}, k_z)a_j(0, k_{0y}, k_z)$. Approximating $\vec{h}_j(0, k_{0y}, k_z)a_j(0, k_{0y}, k_z)$ by an analytic function gives the following approximation for the jth-eigenmode integral,

$$\int_{-\infty}^{\infty} dk_z \frac{\vec{h}'_j(k_z - k_{0jz})a'_j(k_z - k_{0jz})}{i\omega_{ji} - (k_z - k_{0jz})v_{gj}} e^{ik_z z}, \tag{13}$$

where $\vec{h}'_j(k_z - k_{0jz}) = \Theta(k_z - k_{0jz})\,\vec{h}_j(0, k_{0y}, k_{0jz})$, $\vec{a}'(k_z - k_{0jz}) = U^{-1}(k_{0jz})\,\Theta^{-1}(k_z - k_{0jz})\,\vec{A}$, and $\Theta(k_z - k_{0jz})$ is a parameterization of the 16-dimensional special unitary transformations ($SU(16)$) about the identity, with the k_z dependence of the angle linearized, which is described more fully in Section S.4 of the Supplementary Information. The introduction of Θ is really just a formality since the validations below provide that we need never actually evaluate Θ. What matters here is just that we can approximate $\vec{h}_j(0, k_{0y}, k_z)a_j(0, k_{0y}, k_z)$ by some analytic function, and so we can apply contour integration, where the issue of the boundary term is discussed in Section S.4 of the Supplementary Information. Therefore, evaluating the residue at $k_z = k_{0jz} + i\omega_{ji}/v_{gj}$ and closing the contour in the lower-half-plane for $z < 0$, and in the upper-half-plane for $z > 0$, gives the following result for the contribution of the jth eigenmode to the driven steady-state solution,

$$\vec{X}_j(t, \vec{r}) = \pm\frac{1}{v_{gj}}\vec{h}'_j(i\omega_{ji}/v_{gj})\,a'_j(i\omega_{ji}/v_{gj})\,e^{i[\omega_0 t + k_{0y}y + k_{0jz}z + iz\omega_{ji}/v_{gj}]}, \tag{14}$$

where the plus sign applies when $z > 0$, the minus sign applies when $z < 0$, $\vec{k}_{0j} = [0, k_{0y}, k_{0jz}]$, and we note that $\vec{h}'_j(0) = \left.\vec{h}_j\right|_{\vec{k}=\vec{k}_{0j}}$ and $\vec{a}'(0) = \left.a_j\right|_{\vec{k}=\vec{k}_{0j}}$, since $\Theta(0) = I$. For $z > 0$, k_{0jz} is the negative value of k_z such that $\omega_{jr}(k_z) = \omega_0$, and for $z < 0$, k_{0jz} is the positive value of k_z such that $\omega_{jr}(k_z) = \omega_0$. Note that the real part of the group velocity (v_{gjr}) changes sign with k_z, and so there is exponential decay in both the positive and negative directions. Hence the solution (14) includes both negative-going and positive-going waves, which can be separately extracted for use in the TL model.

Using the term "mode" to refer to the contribution from a particular eigenmode, that is, to one of the 16 terms in (12), the linear approximation provides that each propagating mode is approximated by a single decaying wave (which may propagate in either direction), such as for the wave-packet interpretation. It would also be possible to use a higher-order polynomial approximation, in which case the denominator would have multiple zeros, and the contour integration would involve multiple residues. In this case each mode would be approximated by a sum of complementary waves. Although there do not appear to be any

critical obstacles to a higher order implementation, using the single-wave approximations makes the bi-modal model much easier to interpret, and less complex to implement. An increase in order requires calculation of higher order derivatives, which is a more difficult numerical problem. Therefore, in this work we will limit our consideration to the linear approximation, that is, to single-wave representations of the modes. The adequacy of the linear approximation is the major criteria that must be satisfied for the wave-packet interpretation to be valid.

In the case where the linear approximation is known to be accurate there are also some additional criteria. The displacement from k_{0jz} to the location for the residue contains real and imaginary parts,

$$\begin{aligned} k_{jz} &= k_{0jz} + i\omega_{ji}/v_{gj} \\ &= k_{0jz} \pm v_{gji}/(l_{djz}v_{gjr}) \pm i/l_{djz}, \quad \text{where,} \\ l_{djz} &= |v_{gj}|^2 / |\omega_{ji}v_{gjr}|, \end{aligned} \tag{15}$$

and the subscripts r and i denote the real and imaginary parts, respectively. The imaginary part of the displacement, $1/l_{djz}$, provides the lowest order correction to infinite dissipation scale length. But the real part of the displacement, $v_{gji}/(l_{djz}v_{gjr})$, is a correction to k_{0jz}, and if it is not negligible the z-directed wavelength (λ_{jz}) in the solution (14) could be different from $2\pi/k_{0jz}$. Although this still provides for a single-wave representation, it constitutes a correction to the usual wavepacket interpretation. In addition, l_{djz} in equation (15) does not match the expression from the usual wavepacket interpretation, $|v_{gjr}/\omega_{ji}|$, unless $v_{gjr}^2 \gg v_{gji}^2$. And finally, there are corrections to the polarization vectors such that they are not exactly the eigenvectors. However, since we cannot be certain of the linear approximation, we really need an independent evaluation both of it and of these potential corrections to the simple wave packet interpretation.

What we actually want to know is whether an expression of the form (14) provides a reasonably accurate representation of the steady state solution, for some wavelength (λ_{jz}), some dissipation scale length (l_{djz}), and some polarization vector ($\vec{h}'_j$). And, if so, we want values for these three parameters. The only real way to solve this problem is to evaluate the exact integral-form solution (12) numerically. Hence, we undertake to evaluate the steady state solution exactly for a few select cases, using numerical integration.

The first step in doing this is to discuss the issue of the near zone of the source (10), and how it relates to TL theory. The source (10), being an impulse-function in z, excites all wavelengths equally. Hence it has a near zone consisting of contributions to the integrals away from k_z such that real $(\omega_j(0, k_{0y}, k_z)) = \omega_0$, and this near-zone would be different if the source had a different z-dependence. This brings up an important distinction between the meaning of the term "source" in circuit theory (including TL theory), and the application of that term to the "source" (10). TL theory does not actually seek to characterize the response of the system to the particular source (10). Rather, TL theory seeks to characterize the generic behavior of the system when it is placed in an arbitrary circuit involving other generic circuit elements, for example a generic family of sources. While the near-zone will depend very specifically on the exact source excitation, the radiation-zone should depend only on the frequency (ω_0), transverse wavelength (k_{0y}), amplitude ($\vec{A}$), and propagation medium in which the waves from the source must propagate (and in which any reflected waves must propagate). Thus TL theory limits consideration to the radiation zone and thereby achieves a generic or "universal" characterization of the system.

The expedient of assuming the excitation is sufficiently far away may be placed in the same category as linearization, in that they both allow for condensing-out the universal aspects of the system behavior, which will apply under many different circumstances. This is the basic formula for circuit theory, which has been found to have great practical value; it is very often true that the signal amplitude is sufficiently small and the excitation sufficiently far away. Taking the circuit-theory concepts of admittance and conductance into ionospheric science means eschewing the near-zone in order to achieve a universal description of the ionospheric response. Thus, after carrying out each of the numerical integrations in (12), we must identify the radiation-zone of the source.

To evaluate the integrals in the exact solution we simply discretize the kernel of the inverse Fourier transform in (12) and apply the inverse discrete Fourier transform. We take this simple approach because it makes it easy to understand the parameters that govern accuracy. We require that the spectrum be sampled finely enough that the spatial-domain response remains valid beyond the near zone and beyond the expected dissipation scale length, to avoid aliasing. We also require that the spectrum is sampled widely enough

that there is no corruption from windowing, where the inverse Fourier transform of the window is convolved with the desired spatial-domain response. As part of the evaluation we test our sampling on the Fourier transform of the approximate solution (14), which is one of the standard forms having an analytical solution. We consider the ability to reproduce the form (14) as a minimum requirement for the sampling, and then we look for any other evidence that the criteria just mentioned may not be satisfied. When satisfied with the result we identify the radiation zone visually and fit the functional form (14) to a section a couple of wavelengths long. The fit region is usually about 10 to 20 wavelengths from the source, except in the lower E-region where it must be much closer, due to the dissipation scale length being (possibly) less than the wavelength in some cases.

An extended discussion of the validation results is provided in Section S.5 of the Supplementary Information, where three figures are presented. Here we wish just to assert that we feel the usual wave-packet interpretation has been validated, at least in the sense of providing for an important baseline model that may be obtained from the eigenmodes, and which should provide at least a qualitatively accurate description of the ionospheric behavior. The wavelength comparisons all show very good agreement, and with the exception of the Alfvén mode in the lower E region, the polarization vectors also agree very well with the eigenvectors. The dissipation scale lengths do not match so well, but they are never found to be shortened to such a degree as to have an impact (recall from Section 3 that once the dissipation scale is sufficiently long, making it longer does not produce any change). In fact, the dissipation scale found from the group velocity can itself become so short as to have an effect on the Alfvén mode at the bottom of the E region, and the numerical integrations suggest that perhaps they are in fact longer than this, so as to eliminate the effect. However, all in all the numerical integrations serve to reinforce confidence in the usual wave-packet interpretation, with meaningful differences arising only in cases where there are good reasons not to trust the results.

The differences that do arise arise almost exclusively in the lower E region, and involve one of two things: either the sampling required for the Whistler mode is impractical using the computing power available, or the dissipation scale for the Alfvén mode becomes very short, and may not extend past the near-zone of the source. While the first problem is solvable in the future, the second problem creates an ambiguous situation that may be unresolvable; we do not have a recipe for removing near-zone effects. If the source produces a near zone that extends past the radiation zone, then it is not useful for testing our hypothesis. But this does not mean that the eigenmodes do not well characterize the plasma response, under the wave-packet interpretation. Rather, it seems likely that this is just a difficulty arising from the impulse-function feature of the source (10), which excites all wavelengths equally, and therefore is not realistic. We generally expect that radiation-zone waves will preferentially excite other radiation-zone waves, due to their inherent similarity. Thus, it seems likely that our method of validation is simply not applicable to the lower E region, and that the eigenmodes are still the appropriate description of the plasma response, just as they are at higher altitudes.

Fortunately, testing shows that corrections made in the lower E-region do not produce a substantial difference in the results for a signal incident from above. Not very much energy makes it to the lower E region, as will be seen below. Thus we feel it most appropriate to implement the eigenmode-based model in it's pure form, using the usual wave-packet interpretation. This model is a baseline electromagnetic model that is the most natural extension of the current electrostatic baseline.

Handling the Cutoff Modes: The solution (14) does not apply for eigenmodes where there does not exist a k_z such that $\text{real}\,(\omega_j(0, k_{0y}, k_z)) = \omega_0$. In the case of ionospheric science, where ω_0 is generally associated either with a wind-driven dynamo or a magnetospheric source moving near the convection velocity, the so-called "light wave" or "radio frequency" modes are much too high in frequency to satisfy this matching criterion. Inability to satisfy the matching criterion means that these modes do not propagate at the source's frequency and transverse wavelength, and so should not be effective at transmitting energy away from the source. This is why these modes, known to be electromagnetic, are always excluded from analysis of ionospheric and magnetospheric dynamics through some form of low frequency approximation [e.g., *Maltsev et al.*, 1977; *Mallinckrodt and Carlson*, 1978; *Goertz and Boswell*, 1979; *Seyler*, 1990; *Streltsov and Lotko*, 2003; *Otto and Zhu*, 2003; *Dao et al.*, 2013; *Lysak et al.*, 2013; *Tu and Song*, 2019; and many others]. Removing the three radio-frequency modes leaves us with five modes to consider for ionospheric applications.

The eigen-decomposition of H_5 also includes two purely-evanescent eigenmodes having $\text{real}\,(\omega_j) = 0$ (to numerical precision). Since these eigenmodes cannot satisfy the matching criterion and in fact have group

velocity of zero, they also cannot be associated with energy transmission. Hence we are now down to four modes that we might possibly need to consider for ionospheric science.

Although the radio-frequency modes are cutoff and so should have only a parasitic effect, there is one important higher-frequency mode that can propagate in the lower altitude regions of the ionosphere, but which becomes cutoff above a certain altitude. This is the mode that we refer to as the Whistler mode. We need a mathematical treatment for the cutoff of the Whistler mode so that it can be included in the model. Therefore, in Appendix B we derive an additional, quadratic integral approximation applicable to the higher frequency modes, which models both the propagation and low-frequency cutoff of these modes. The resulting solution (B.3) supports the assessment that cutoff modes will not contribute significantly to the transmission of energy; the dissipation scale length decreases dramatically across the k_{0y} threshold where $\mathrm{real}\,(\omega_j(0, k_{0y}, k_z)) = \omega_0$ cannot be satisfied for any k_z. Away from cutoff, the solution (B.3) reduces to the solution (14), and so the validation just described applies to both of these approximate solutions.

One other of the four ionospheric-frequency modes can also become cutoff, except with the cutoff occurring at high frequencies, instead of low. However, even when this mode does propagate (i.e., satisfy $\mathrm{real}\,(\omega_j(0, k_{0y}, k_z)) = \omega_0$), it has a very short dissipation scale length (see Sections 6.1 and 6.2). So this mode is also not relevant to the transmission of energy through the ionosphere. Also, it disappears when the energy equations are omitted from the analysis (e.g., the analysis in *Cosgrove* [2016]), and the energy equations should not be required to derive electrostatic theory. So although a solution very similar to (B.3) should be applicable, we have not yet derived a solution for this mode, which we call the Thermal mode.

The remaining two ionospheric-frequency modes do not seem to be susceptible to cutoff, and so our original solution (14) is applicable. We call these modes the Alfvén and Ion modes. (See Section 6 for a discussion of the modes and their naming.)

What is important for this work is that there do exist relatively low-loss modes for which one of the solutions (14) or (B.3) should be a valid approximation, and these modes are found (below) to dominate energy transmission through the ionosphere. In Section 6.1 we show that electrostatic waves cannot be used as approximations for these electromagnetic modes, by means of a quantitative evaluation of the parallel wavelength, dissipation scale length, and characteristic admittance as a function of transverse wavelength and altitude. In Section 6.2 we make the argument for retaining only two modes, and perform a “back of the envelope” calculation that predicts wavelike effects. And in Section (7) we present first results from our model calculation, which development is completed in the following Section (5).

5 Electromagnetic Modeling for the Ionosphere

The steady-state solutions found in Section 4 apply for small deviations from thermal equilibrium, which means that they apply for small deviations from a homogeneous plasma configuration. We would like to extend these solutions to ones that apply to small deviations from an inhomogeneous plasma configuration like the ionosphere. This situation is similar to the one that exists for electrostatic theory: although technically electrostatic theory applies to small deviations about thermal equilibrium (see Appendix A), it is often applied to other background states that are just assumed to satisfy the equations of motion, even though they may not actually do so. The idea is that if we observe this state in nature, it must be some kind of quasi-equilibrium of the actual physical system, which may not be adequately described by the equations of motion. This gives a heuristic justification for just pretending that some observed state satisfies the equations of motion (A.1), and using it as a background. Hence we will adopt this same justification and use TL theory to extend our driven steady-state solutions (12) and (B.3) to ones that apply to small perturbations about a background state with an arbitrary inhomogeneity in one dimension (the vertical dimension).

A wave propagating along a waveguide with varying characteristic admittance can be modeled in steady-state by cascading together a number of short sections of transmission line having constant characteristic admittance, and imposing boundary conditions at each interface to determine the transmitted and reflected components. Similarly, a wave propagating in a three-dimensional medium that is inhomogeneous in one direction only can be modeled in steady-state by stacking a number of thin homogeneous slabs, and imposing boundary conditions at each interface to determine the transmitted and reflected components. Hence, adopting the language and imagery of TL theory and labeling the thin slabs as TL sections, we propose to estimate the ionospheric admittance and model the ionosphere using a cascade of short transmission line

sections stacked in the vertical direction, as represented in panel e of Figure 1. The model applies to the far zone for sources of the form (10), where we can regard $\lambda_y = 2\pi/k_{0y}$ as the relevant transverse scale, while eventually planning to form transverse wavepackets by regarding each solution as a Fourier component, and superposing the solutions from a spectrum of λ_y. Although this geometry assumes a vertically stratified ionosphere, the geomagnetic field can be tilted. At a higher level of approximation we can consider geometries adapted to curved geomagnetic field lines and wave packets guided along them, in order to treat ionospheric phenomena at low and equatorial latitudes, such as the pre-reversal enhancement, and equatorial spread-F (see Section 1).

Solving the cascade of line sections (panel e of Figure 1) involves solving for the modal coefficients in each line section, such that the boundary conditions are satisfied across each interface. By "modal coefficients" we mean the complex constants that are applied either to the solution (14) or the solution (B.3), as appropriate for the mode, in order to adjust the amplitude and phase. The positive-going or negative-going halves of each solution will be separately employed, as determined by causality. The complete solution for the potentially large number of boundaries can be obtained by bootstrapping from the last section of line: the input admittance to the last section of line is obtained, and then used as the load terminating the second to last section to obtain its input admittance, which is used as the load for the third to last section, etc., until the input to the cascade is reached.

However, the inclusion of multiple modes means that the input admittance can no longer be represented by a single complex number. In general, the admittance is a two index tensor, where one index runs over the modes to be retained and the two directions, and the other index runs over the components of $\vec{X}$ for which boundary conditions will be enforced (the tensor must be square). We will use the term "polarization vector" to refer to the part of the admittance tensor associated with a particular mode and direction. To establish an equation for the boundary condition joining the last two sections of transmission line in panel e of Figure 1, consider a multi-modal component $\vec{E}_N$ incident on the Nth boundary, and originating at the $(N-1)$th boundary. ($\vec{E}_N$ contains only the components of $\vec{X}$ for which boundary conditions will be enforced.) Using the solution (14) (for brevity, both integral approximations will be used in combination), and dropping the temporal and transverse dependence (which are the same for all modes), this incident component takes the form,

$$\begin{aligned} \vec{E}_N(z) &= \sum_{\substack{j(l) \\ l\in n_{N-1,+}}} \frac{1}{v_{gj}} \vec{h}''_j \varepsilon_j \mathrm{e}^{(z-z_{N-1})(ik_{0zj}-\omega_{ji}/v_{gjr})_{\vec{k}=\vec{k}_{0j}}} \\ &= E_{N-1}(z)\vec{\varepsilon}_N \end{aligned} \tag{16}$$

where z_{N-1} is the location of the $(N-1)$th boundary, $n_{N-1,+}$ is the set of retained modes within the $(N-1)$th line section that were generated at the $(N-1)$th boundary (+ indicates that these are positive-going waves), $E_{N-1}(z)$ is a matrix, $\vec{\varepsilon}_N$ is the vector with elements $\{\varepsilon_j\}$, and the double-prime on the eigenvector $\vec{h}_j$ indicates removing the elements for which boundary conditions will not be enforced. Equation (16) defines the notation $\left(\vec{E}_N(z), E_{N-1}(z), \vec{\varepsilon}_N\right)$ for describing the wave incident on the Nth boundary, where the matrix carries the subscripts $N-1$ to indicate that the wave is traveling in the $(N-1)$th line section, and the coefficient $\vec{\varepsilon}$ and wave $\vec{E}$ carry the subscripts N to indicate that the wave participates in the boundary condition at the Nth boundary. In the same way we define the notation $\left(\vec{B}_N(z), B_{N-1}(z), \vec{b}_N\right)$ and $\left(\vec{C}_N(z), C_N(z), \vec{c}_N\right)$ for the reflected and transmitted components associated with the Nth boundary condition, respectively. The reflected component employs the negative-going waves, in this case $n_{N-1,-}$, by the notation above. Note that this notation can be employed for any of the boundaries, and regardless of which solution, (14) or (B.3), has been used for the various modes.

At the boundary between the Nth and $(N-1)$th sections of line (the Nth boundary), the component $\vec{E}_N$ produces the transmitted component $\vec{C}_N$ in the Nth section, and the reflected component $\vec{B}_N$ in the $(N-1)$th section. The component $\vec{E}_N$ is, in turn, produced by the component $\vec{E}_{N-1}$ incident on the $(N-1)$th boundary, and so on. This association provides that there is a constraint, $\vec{c}_{\alpha-1} = \vec{\varepsilon}_\alpha$. We are now in a position to describe the bootstrapping procedure.

For illustration, assume that the boundary conditions are simply those of continuity (which is our actual case). The boundary condition at the Nth boundary can be written $\vec{E}_N\Big|_{z=z_N} + \vec{B}_N\Big|_{z=z_N} = \vec{C}_N\Big|_{z=z_N}$,

where $\vec{B}_N$ represents the modes in the $(N-1)$th section that were generated at the Nth boundary, and $\vec{C}_N$ represents the modes in the Nth section and generated at this same boundary. Since there are no boundaries beyond the Nth boundary (panel e of Figure 1), the Nth boundary condition is unique in that there is no need to consider reflections of $\vec{C}_N$. Using the notation just developed, the boundary condition at the Nth boundary allows for expressing $\vec{E}_N$ in terms of the modal coefficients for the transmitted and reflected components,

$$\begin{aligned} \vec{E}_N\Big|_{z=z_N} &= C_N(z_N)\vec{c}_N - B_{N-1}(z_N)\vec{b}_N \\ &= [C_N(z_N)\cup B_{N-1}(z_N)]\begin{bmatrix} \vec{c}_N \\ \vec{b}_N \end{bmatrix} \end{aligned} \tag{17}$$

where the symbol $\cup$ indicates appending the columns of one matrix to another, with a negative sign applied to the second. Note that the presence of evanescent modes would render the matrix $[C_N \cup B_{N-1}]$ non-invertible, because the evanescent modes would be present in both C_N and B_{N-1}. Hence, this formulation requires that only propagating modes are retained.

A bootstrapping procedure analogous to that for the single mode case can be used to solve the transmission line cascade of Figure 1, panel e. Beginning with the last (Nth) boundary, combine equations (16) and (17) and solve for the modal coefficients $\left(\vec{c}_N,\, \vec{b}_N\right)$ at the Nth boundary, in terms of the modal coefficients $\vec{c}_{N-1}$ at the $(N-1)$th boundary,

$$\begin{aligned} \begin{bmatrix} \vec{c}_N \\ \vec{b}_N \end{bmatrix} &= [C_N(z_N)\cup B_{N-1}(z_N)]^{-1}\, \vec{E}_N\Big|_{z=z_N} \\ &= [C_N(z_N)\cup B_{N-1}(z_N)]^{-1}\, E_{N-1}(z_N)\vec{\varepsilon}_N \qquad (18) \\ &= [C_N(z_N)\cup B_{N-1}(z_N)]^{-1}\, E_{N-1}(z_N)\vec{c}_{N-1}, \qquad (19) \end{aligned}$$

where we have used the constraint $\vec{\varepsilon}_N = \vec{c}_{N-1}$ noted above.

Now moving to the $(N-1)$th boundary, the component $\vec{E}_{N-1}$ produces the component $\vec{E}_N$, but associated with $\vec{E}_N$ is the reflected component $\vec{B}_N$, so that effectively $\vec{E}_{N-1}$ produces a "transmitted" component $\vec{C}_{N-1} = \vec{E}_N + \vec{B}_N$ in the $(N-1)$th line section. To write the equation for the $(N-1)$th boundary in a form similar to (17), we need an equation for $\vec{C}_{N-1}$ in terms of $\vec{c}_{N-1}$ alone, which we can obtain using the result (19) from the Nth boundary,

$$\begin{aligned} \vec{C}_{N-1}(z) &= \vec{E}_N(z) + \vec{B}_N(z) \\ &= \Big(E_{N-1}(z) + B_{N-1}(z)\overline{[C_N(z_N)\cup B_{N-1}(z_N)]^{-1}}E_{N-1}(z_N)\Big)\,\vec{c}_{N-1} \\ &= C_{N-1}\vec{c}_{N-1}, \end{aligned} \tag{20}$$

where the over-bar indicates that the top half of the rows have been removed, so that only $\vec{b}_N$ is present in the output.

The columns of C_{N-1} are a set of effective steady-state modes for transmission into the $(N-1)$th line section. Thus we can write the $(N-1)$th boundary-condition equation, $\vec{E}_{N-1}\Big|_{z=z_{N-1}} = \vec{C}_{N-1}\Big|_{z=z_{N-1}} - \vec{B}_{N-1}\Big|_{z=z_{N-1}}$, in the form of equation (17), and obtain the invertible matrix $[C_{N-1}(z_{N-1})\cup B_{N-2}(z_{N-1})]$ for the next iteration: $\Big[\vec{c}_{N-1};\, \vec{b}_{N-1}\Big] = [C_{N-1}(z_{N-1})\cup B_{N-2}(z_{N-1})]^{-1}\, E_{N-2}(z_{N-1})\vec{c}_{N-2}$.

Continuing to iterate to the input of the cascade we finally obtain,

$$\begin{bmatrix} \vec{c}_1 \\ \vec{b}_1 \end{bmatrix} = [C_1(z_1)\cup B_0(z_1)]^{-1}\, \vec{E}_1(z_1),$$

which allows for finding the reflected and transmitted components associated with a component $\vec{E}_1$ incident on the modeled region. The coefficient $\vec{b}_1$ determines the reflected component through the relation similar

to (16), and the coefficient $\vec{c}_1$ determines the transmitted component through the relation similar to (20). The input admittance to the modeled region follows directly from this result, as do all the field quantities at all altitudes.

If there were no evanescent modes then, in principle, the above would provide a general procedure for deriving the complete solution for the cascade of line sections. Otherwise, the evanescent modes could be removed and there should be only a very small effect, since we do not expect them to be excited by incoming waves and they cannot transmit energy. However, there is one critical piece missing: implementation requires knowledge of the physical boundary conditions for all the retained components of $\vec{X}$. In fact, we only know of three unambiguous boundary conditions: continuity of the two components of electric field parallel to the boundary, and continuity of the single component of magnetic field perpendicular to the boundary. To include even two modes we need one additional boundary condition. For this fourth boundary condition we will use continuity of the component of current density perpendicular to the boundary, which provides that the closure of the field aligned current through transverse currents will happen only through the plasma within the line sections, and will not happen through boundary terms. An electrostatic treatment would not involve any such boundary terms, and so, among other justifications, we will use this boundary condition in order to allow for the possibility of reproducing electrostatic theory.

In what follows we argue that there are, in fact, only two modes that are effective in transmitting energy through the ionosphere, and that only these two modes need be included to produce a suitable model. These two modes can be modeled by the solutions (14) and (B.3). In the next section we find that they are roughly analogous to the Alfvén and Whistler waves, respectively, although our formal definition does not map one-to-one to the usual physically-defined waves.

Examples of the ionospheric input admittance computed using one and two mode versions of the model are presented in Section 7, along with some selected field quantities resolved in the vertical direction. Note that in practice we follow the matrix inversions with optimization steps, to ensure that the boundary conditions are enforced to the full numerical precision available.

6 Wave Modes Relevant to Ionospheric Science

The solution (9) to the initial value problem allows us to understand the ionospheric plasma motion as a sum of characteristic modes, which will begin to couple as the excitation becomes larger and nonlinear terms become important, but which will evolve independently for small excitations of a homogeneous background. A source excites the characteristic modes and those that are able to propagate will carry energy away, through the ionosphere, while gradually dissipating. As time goes to infinity a steady state condition arises where the plasma oscillates harmonically with the source, and also matches the source in the transverse spatial dimensions (from equation (12)). At any appreciable distance the only remaining modes are those that can propagate at the source-frequency and transverse wavelength. In this context we can determine which modes are relevant to ionospheric physics, and which can be safely ignored, by considering typical ionospheric scale sizes and drift velocities.

6.1 Basic Properties of the Modes and Comparison to Electrostatic Waves

Applying numpy.linalg.eig from the Numpy Python library we find the eigenvalues and eigenvectors of the matrix H_5, which was introduced in Section 4. Searching the transverse wavelength range from 100 km to 1000 km, with a 40 m/s transverse phase velocity, we find that the electromagnetic five-moment fluid equations support four modes that can propagate at the determined frequencies $\left(\text{i.e., } \frac{40\,\text{m/s}}{\lambda_\perp}\right)$, in the plasma density range $4.7 \times 10^9\,\text{m}^{-3}$ to $1.0 \times 10^{11}\,\text{m}^{-3}$, and altitude range 100 km to 400 km. The three radio-frequency modes cannot propagate, and neither can the potential lowest frequency mode, which decouples into two evanescent (zero frequency) eigenmodes over this range (to numerical precision). Thus all 8 of the modes that could be supported by the set of 16 equations have been accounted for.

The 40 m/s phase velocity is easily small enough that electrostatic theory would generally be considered applicable, and we will restrict our considerations to this phase velocity. Smaller wavelengths and larger phase velocities were investigated by *Cosgrove* [2016], who found only two propagating modes, although using a smaller set of equations. In fact the two additional propagating modes that we find are enabled

by the larger equation set (see Section 4), and could not have been found in the *Cosgrove* [2016] study. In decreasing order of real-frequency, we refer to the four propagating modes as the Whistler, Alfvén, Ion, and Thermal modes. Note that these are only names for eigenvectors, and so should not be equated directly to physically defined waves, such as the MHD waves.

In the first-order integral approximation the modes take the form of single decaying waves (Section 4), but we will continue referring to them as modes since they are actually steady-state structures, and also to minimize any confusion that may arise in comparisons to physically defined waves. There may be more physical wave-types than there are eigenvectors, and so there does not exist a one-to-one correspondence between the two. Our names refer to eigenvectors that have been identified over a range of collision frequencies (range of altitudes), and so may actually include more than one physical type, in some cases. For example, in *Cosgrove* [2016] the usual dispersion relations for the whistler and fast-magnetosonic waves were found to produce frequencies within an order of magnitude of each other for the studied conditions, and so seemed to correspond to the same eigenvector. The whistler wave was a better fit, and so that name was adopted; but it may not be a better fit at all altitudes in the ionosphere. There is also an issue in that different subfields of space physics may consider different naming schemes to be appropriate. For example we derive the name Ion mode from what ionospheric physicists call the ion-acoustic wave, but magnetospheric physicists may wish to call this mode the slow-magnetosonic wave. Since the goal of this work is to derive the ionospheric conductance, we focus on the E-region manifestations and use the terminology that we think is most comfortable for ionospheric physics.

Figure 2 shows results using a background plasma density of 10^{11} m^{-3} (at all altitudes), with the remaining parameters determined by the same altitude profiles used in *Cosgrove* [2016] (see their Appendix B, and included for reference in Section S.2 of the Supplementary Information). Although the legend includes all four wave modes, sometimes a particular figure will not have a corresponding line, or will only have a partial line, indicating that the frequency matching condition could not be satisfied, and so the mode does not propagate. For example, the Whistler mode was not able to satisfy the matching condition in the F region, and demonstrated a short wavelength cutoff that increased with altitude. Solid lines indicate the full electromagnetic results (i.e., using H_5), and dashed lines indicate the most comparable electrostatic modes found using the matrix H_{5ES}, which is equivalent to H_5 except using the assumption that the curl of the electric field is zero (which amounts to substituting the Poisson equation for the Maxwell equations, as explained in detail in Appendix A). In the case of the electrostatic modes, absence of a line may also mean simply that it is outside of the axis limits. The Farley mapping distance is also shown in the panels for dissipation scale length, as it represents the comparable electrostatic quantity.

Panels a and c of Figure 2 show the wavelength in the direction along the geomagnetic field (λ_z) for altitudes of 100 km and 145 km, respectively, plotted versus transverse wavelength. With the exception of the Whistler mode, the λ_zs are generally quite short, especially at the lower altitude, and for the Thermal and Ion modes, with 10 km being a representative number. Where the electromagnetic and electrostatic modes agree the dashed lines should lie on top of the solid lines. Examining the figure, the Ion and Thermal modes appear to be well described by the corresponding electrostatic modes. However, the Alfvén mode appears to be electrostatic only up to about 100 m in transverse wavelength, and then radically diverges, with the associated electrostatic mode generally having a much longer λ_z. And the Whistler mode appears to have no electrostatic counterpart at all. Similar results are known in the context of collisionless MHD waves, but we are not aware of equivalent results for the collisional ionospheric modes.

Panels b and d of Figure 2 show the dissipation scale length (l_{dz}) for altitudes of 100 km and 145 km, respectively, plotted versus transverse wavelength. (Note that the group velocity is calculated as a finite difference, and so l_{dz} is subject to more numerical error than λ_z.) The Ion and Thermal modes are seen to have very short dissipation scale lengths, 1 km or less, and at the lower of the two altitudes the Alfvén mode is also quite short, peaking at 50 km. For comparison, the black line in the panel shows the Farley mapping distance, which is the commonly accepted estimate for the electric field mapping distance (derived from electrostatic theory); clearly the agreement is marginal at best. Comparing the electromagnetic and electrostatic modes, l_{dz} and λ_z give a similar threshold for the transition to electrostatic behavior for the Alfvén mode, i.e., below about 100 m.

Looking at panel b, at the lower altitude (100 km) the Whistler mode appears to takeover for the Alfvén mode as the transverse wavelength becomes longer, and the dissipation scale length for the Alfvén mode is

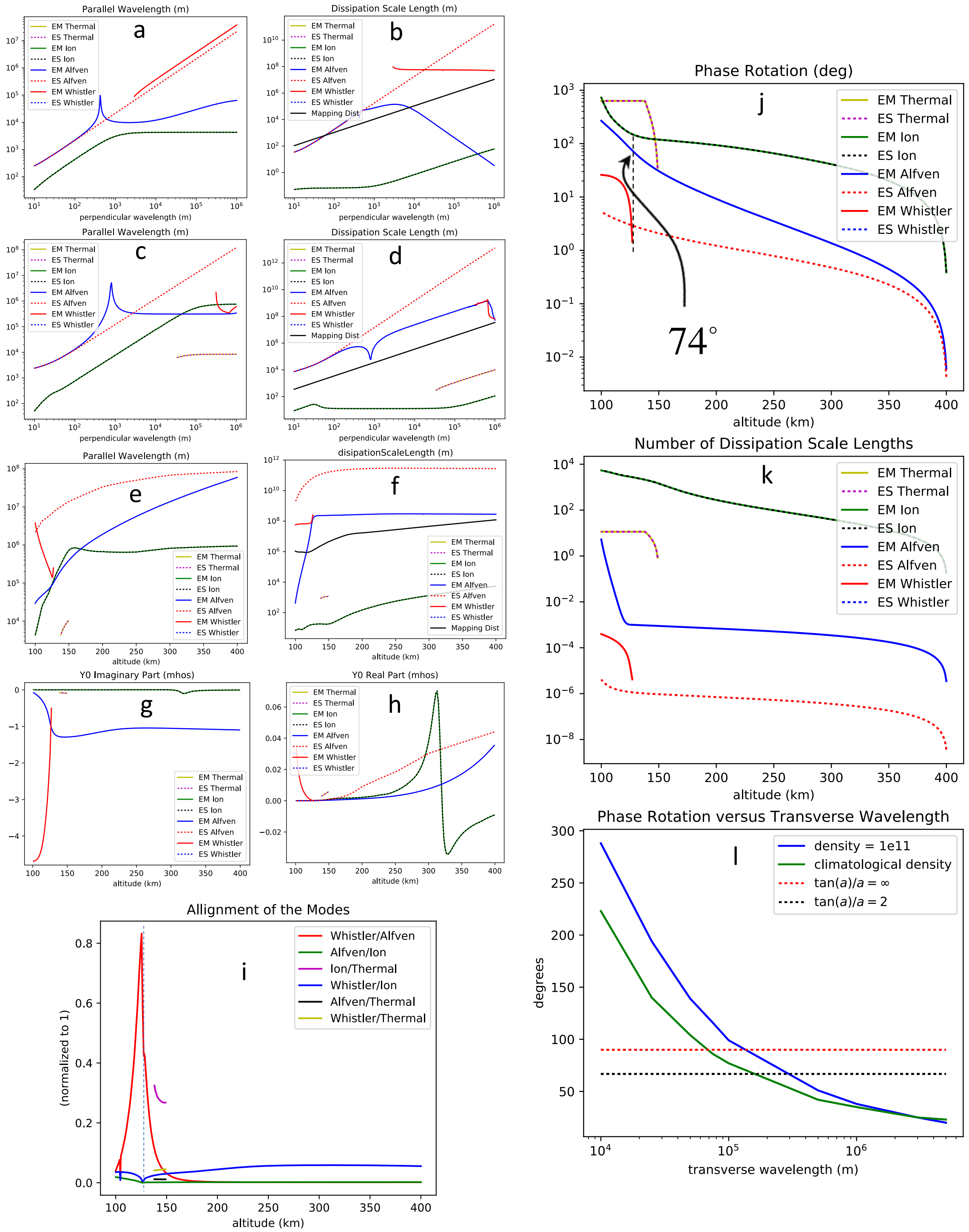

Figure 2: Wave properties for the relevant modes. For panels a-i, rows 1 and 2 are for altitudes of 100 km and 145 km, respectively, and rows 3, 4, and 5 are for a 100 km transverse wavelength. Panels j-l demonstrate a rough analysis of the phase rotation expected for a signal traversing the ionosphere, also for a 100 km transverse wavelength. The amount of phase rotation (panel j) and number of dissipation scale lengths (panel k) are integrated from 400 km in altitude. Panel l combines the phase rotations of the Alfvén and Whistler modes to get an estimate for the electrical thickness of the ionosphere, versus transverse wavelength: the dashed red line indicates 90°; and the dashed black line indicates 66.8°, where the electrical length is just sufficient to double the input conductance. The plasma density is 10^{11} m^{-3} in all the panels and at all altitudes, excepting the green curve in panel l, where the phenomenological density profile from *Cosgrove* [2016] is used.

seen to drop below 1 km. Also, comparing panels a and b with panels c and d, respectively, shows that the Whistler mode requires a longer transverse wavelength at higher altitudes, in order to be a propagating mode. And finally, at the higher altitude a kink has appeared in the curves for the Whistler mode. We later find that this kink is associated with a change in its physical character. It turns out that these features are associated with major factors that influence the wavelength dependence of the input admittance studied below.

Panels e, f, g, and h of Figure 2 show λ_z, l_{dz}, and the real and imaginary parts of the characteristic wave admittance, respectively, plotted versus altitude, for a 100 km transverse wavelength. Comparing the solid and dashed lines in all the panels shows that the Alfvén and Whistler modes cannot be approximated by electrostatic waves for this transverse wavelength (100 km), regardless of altitude. The wavelength for the electrostatic mode is an order of magnitude too long, and the characteristic admittance is very different (it is outside the axis limits). And there still does not appear to be any electrostatic counterpart for the Whistler mode, that is, there are more propagating electromagnetic modes than there are propagating electrostatic modes. The Thermal mode does seem to be well approximated by its electrostatic counterpart, but only arises over a narrow altitude range, around 150 km. The Ion mode also seems to be well approximated by its electrostatic counterpart, but has a very short dissipation scale length. Comparing panels c and d shows that for the Alfvén and Whistler modes, the imaginary parts of the characteristic admittance are much larger than the real parts, verifying the assertion made in Section 3. And they are also negative, which provides that the wave-Pedersen conductivity be positive and (mostly) real, as expected (equation (6)).

6.2 Scenario for Energy Transfer Through the Ionosphere, and the Bi-Modal Model

For energy (and thus electric field, etc.) to be transmitted from, say, 400 km down to lower altitudes, waves must propagate downward while encountering varying conditions that may cause them to reflect and/or to be coupled into other modes at the same frequency and transverse wavelength. Some of this coupling may be into modes that cannot propagate under these condition, but such modes cannot transport energy, and so are essentially loss terms. When conditions are uniform, coupling between different modes can occur only through the nonlinear term, $\vec{F}'$. However, when conditions change with altitude (e.g., collision frequency changing) the wave modes also change with altitude, and so have potential to couple to one-another through this effect (unlike that due to $\vec{F}'$, this form of coupling does not decrease with wave amplitude). In the TL theory calculation, this form of coupling occurs at the boundaries between different TL sections. The coupling potential is increased for eigenmodes that are not strongly orthogonal, since in this case small variations in the background conditions have a much greater affect on the modal allotment. Note that in this subsection we will eschew our practice of avoiding the term "wave," because of the intuitive benefits associated with that term for this particular discussion. There is some additional discussion of the language ambiguities that we face at the beginning of Section 7.

Focusing on the example of a 100 km transverse wavelength, Figure 2 (panels e-h) shows that there are only two propagating waves between 400 km and 150 km in altitude, the Alfvén wave and the Ion wave. Panel f shows that the dissipation scale length for the Ion wave is only about 10 km at 400 km in altitude, and decreases further with decreasing altitude. Although propagating, the Ion wave cannot transmit energy over the considered distances, and so any signal arriving from the magnetosphere must arrive in the form of an Alfvén wave. Any energy coupled from the Alfvén wave into the Ion wave will be dissipated without further transmission. This also applies to the Whistler wave; although non-propagating in this altitude range, some energy could be coupled into it, and that energy would be rapidly dissipated. So how much coupling will there be?

Panel i of Figure 2 shows a representation of the degree of alignment of the different modes, specifically, the complex-conjugate dot-product between the eigenvectors (which are normalized to unit-length). Modes that are already partially aligned can be expected to couple more strongly across the TL section boundaries, whereas modes that are orthogonal should have minimal coupling. The figure shows that the Alfvén wave is nearly orthogonal to the Ion wave, and in the region above 150 km is also orthogonal to the Whistler wave. The vertical dashed line in the figure marks the altitude where the Whistler begins propagating, whereas the other waves can propagate everywhere they are shown. (Recall from Section 4 that we have developed a solution for the Whistler wave that includes its non-propagating regions, which we are using here.) There

is some alignment between the Whistler and Ion waves, but since the Whistler wave does not begin to align with the Alfvén wave until below 150 km, and does not begin propagating until below 127.5 km, it should not hold much energy until below these altitudes, where its alignment with the Ion wave is very slight. So there is no clear pathway for delivering energy to the Ion wave.

The thermal wave comes briefly available between about 140 km and 150 km in altitude, as can be seen from any of panels e-i in Figure 2. This mode also will not transmit energy, since it also has a very short dissipation scale length (panel f of Figure 2). However, the Thermal wave has dissipation time-scale longer than any other mode (not shown). Therefore, it seems possible that the Thermal wave will fill up with energy. Since the Thermal wave has a very short parallel wavelength of around 10 km, this could create a significant anomaly. However, panel i of Figure 2 shows that the Thermal wave does not have much alignment with either the Alfvén or Whistler waves. Also, the Thermal wave does not propagate if the phenomenological density profile of Figure S.2 (in Supplementary Information), which has a lower density in this region, is substituted for the constant density profile used in Figure 2. Also, the Thermal wave disappears when the energy equations are omitted (not shown). Therefore, it seems that the Thermal wave should only produce a minor anomaly, except possibly under some particular conditions that favor it.

At 127.5 km in altitude the Whistler wave begins to propagate. It is right at this altitude that the dissipation scale length for the Alfvén wave begins to decrease dramatically, while the wavelength continues to shorten. So if the majority of the incident energy is to make it down to 100 km, there must be very efficient coupling from the Alfvén wave to the Whistler wave, such that most of the energy is transferred to the Whistler wave. From panel i of Figure 2, the Whistler and Alfvén waves begin to align strongly below 127.5 km in altitude, with peak alignment occurring at 125.5 km. There appears to be a degeneracy or near-degeneracy of the two modes at this altitude, and so there is potential for strong coupling into the Whistler wave. However, it seems likely that this condition would result in a splitting of the energy between the continuing Whistler and Alfvén waves, along with partial back-reflection of the Alfvén wave due to the impedance mismatch caused by the new mode suddenly becoming available.

So in order to evaluate this effect we need to implement the TL theory calculation of Section 5, and both the Whistler and Alfvén waves must be included. However, we have made the case that the Ion and Thermal waves have only parasitic effects, and that these effects will only further any deviations from electrostatic theory that the model may otherwise find. Therefore, in order to accommodate the limited number of boundary conditions that can be clearly determined, we will assume that no energy is lost to either the Ion or Thermal waves, and omit those waves.

Before studying the results of the full TL theory calculation it is useful to make a simple, best-case analysis of the electrical length (phase rotation) associated with energy transfer through the ionosphere. So consider the case where the Alfvén wave energy is completely transferred to a downward Whistler wave at 127.5 km, the Ion wave does not siphon much energy, the Thermal wave does not siphon much energy, and back-reflection is insubstantial until the wave meets with the bottom of the E region. We also ignore energy coupled into non-propagating modes, since this effect would only serve to hinder energy transfer. To analyze this case we integrate the inverse of the parallel wavelength (panel e of Figure 2) over altitude, from 400 km downward, and display the amount of phase rotation as a function of altitude in panel j of Figure 2 for each mode. When the Whistler wave first begins propagating at 127.5 km in altitude, the Alfvén wave has already rotated 74°. Here the energy is assumed transferred into the Whistler wave, where, again from panel j, there is an additional 25° of phase rotation on the way down to 100 km in altitude. So the transmission line from 400 km down to 100 km has an electrical length of $74° + 25° = 99°$, which we might call the electrical thickness of the ionosphere for a 100 km transverse wavelength and density of 10^{11} m^{-3}. If instead we use the phenomenological density profile from *Cosgrove* [2016], which has a low-density valley region separating the F region from an E region arc, we find a reduced electrical thickness of 77°.

Panel k of Figure 2 shows the results of doing the same integration for the dissipation scale length. Again using the assumption that the energy is completely transferred to the Whistler wave at 127.5 km, the amount of dissipation is seen to be negligible. The Alfvén wave does become significantly dissipative, but only at the very bottom of the ionosphere, where the Whistler wave may be dominant. Thus it appears that the ionosphere can be described by waves that are essentially lossless, which is the usual case for TL theory, and we verify this in Section 7, where the loss is included. However, it appears that the electrical thickness of the ionosphere is not negligible (for the 100 km transverse wavelength and density of 10^{11} m^{-3}).

A 90° phase rotation means that a maxima at 400 km in altitude gives rise to a zero at 100 km in

altitude. So a 90° electrical thickness can be considered as the threshold where the electric field completely fails to map through the ionosphere. As we have seen in Section 3, equation (5), the input admittance to a section of transmission line terminated in an open circuit is proportional to the tangent of the electrical length. Therefore, a 90° electrical thickness gives rise to a short circuit condition at 400 km in altitude, meaning that the wave reflected back from the bottom of the ionosphere completely cancels the electric field of the incident wave, at 400 km. In this case the field line integrated conductivity is completely inapplicable as a measure of the ionospheric admittance. (Note, although wave dissipation is slight, it will nevertheless produce a small imaginary component in the electrical thickness that we are ignoring in this discussion, which will cause the cancelation of electric field to be imperfect. This lossy effect is, however, included in the model.)

Repeating the analysis for other wavelengths and one other density profile, the electrical thickness increases with decreasing transverse wavelength, and increases with density. Two plots of electrical thickness versus transverse wavelength are shown in panel l of Figure 2, one for the case under discussion (density 10^{11} m^{-3} at all altitudes, blue line), and one for the phenomenological density profile from *Cosgrove* [2016] (green line). For example, for the case under discussion, decreasing the transverse wavelength to 50 km adds 40° of electrical length, and decreasing the transverse wavelength to 25 km adds another 55°, that is, it adds 95°. So beginning at around 100 km in transverse wavelength (actually somewhat larger), the electric field may not map through to the bottom of the E region, and there may even be positive-feedback effects similar to the ionospheric feedback instability [*Sato*, 1978; *Trakhtengertz and Feldstein*, 1984; *Lysak*, 1991; *Pokhotelov et al.*, 2001; *Streltsov and Lotko*, 2003; *Cosgrove and Doe*, 2010; *Akbari et al.*, 2022].

For longer transverse wavelengths the electrical thickness decreases, and becomes negligible. However, other potentially important effects need to be considered. It seems not unlikely that the dividing of energy between the Alfvén and Whistler waves, and/or back-reflection of the Alfvén wave, could have a significant effect. Therefore, to include these effects, we have implemented the TL theory calculation described in Section 5, using the Alfvén and Whistler waves.

7 Model Results

The model of Section 5 has been implemented using two modes, which in the first-order approximation take the form of decaying waves, as described in Section 4. By "decaying waves," we mean that these are steady-state structures, not dynamical waves, and so we are modeling the steady state of the system. There is some difficulty in language in that it is sometimes convenient to refer, instead, to the dynamical structures that evolve toward these steady states, and also to relate these to the physically-defined waves that arise in other contexts. Hence we sometimes refer to the two modes as the Alfvén and Whistler waves, although we emphasize that there does not exist a one-to-one mapping to the usual physically defined waves. Besides the distinction of being steady-state structures, there is also the distinction that the "waves" in our model are defined over the full altitude extent of the ionosphere, and thus change their physical character with altitude (Section 6.1). Although changes in plasma density generally occur with altitude, in this case we are referring to physical changes caused by the changing collision frequency, not by changes in plasma density. In order to simplify the various effects that must be unraveled we keep plasma density constant with altitude in all the examples below.

Hoping that the reader can maneuver these ambiguities of language, we now relax somewhat and describe the model as accounting for the excitation, transmission, reflection, and coupling of the Alfvén and Whistler waves, as they carry energy through the ionosphere. Mode-mixing occurs because the waves change their nature with altitude, because of the changing collision frequency. And also the waves may be dissipative, and drop-out as they dissipate. Besides the parasitic modes (Sections 4 and 6.2), what is not included in the model is the nonlinear coupling between waves that happens within each uniform section of line, which becomes negligible for small amplitude excitations, and which is not part of the definition for admittance (a quantity from linear circuit theory). We note that the same situation applies for electrostatic theory, which drops the same nonlinear terms.

For pedagogical purposes we have also implemented the model using one mode at a time. This requires dropping two of the physical boundary conditions. Using the convention that the x-component of the wavevector is zero ($k_x = 0$, which we use throughout), and placing the z-axis along the geomagnetic field

(assumed vertical for brevity), the omitted boundary conditions are continuity of the x-component of electric field (E_x), and continuity of the z-component of magnetic field (B_z). To the extent that the bi-modal model predicts mode mixing, the one-mode models are unphysical because they do not respect these necessary boundary conditions. However, comparison of the one and two mode models helps us to understand which of the effects are caused by mode mixing, and which are caused simply by the collision-frequency-dependent nature of the Alfvén wave.

For all the examples to follow the transverse wavelength is related to the frequency by assumption of a transverse velocity of 40 m/s, and (repeating) the plasma density will always be kept constant with altitude. The remaining ionospheric parameters are the same as used in *Cosgrove* [2016] (see their Appendix B, and included for reference in Section S.2 of the Supplementary Information). The examples all employ one-kilometer-long TL sections, with the exception of the summary of results shown in Figure 7, which are described in Section 7.4. An open-circuit boundary condition is employed at the bottom of the ionosphere, although we find below that the results are not very sensitive to this condition.

7.1 Artificial Examples that Test the Model, and Reproduction of Electrostatic Theory

We begin with an artificial example that shows how the model would reproduce electrostatic theory, if the waves had the right characteristics. In Section 3 we explained that a single-wave theory will reproduce electrostatic theory if three conditions hold: (1) the wave-Pedersen conductivity is equal to the zero-frequency Pedersen conductivity; (2) the wavelength is long compared to the system size; and (3) the dissipation scale length is long compared to the system size. For our two-wave theory we also have the additional requirements that the waves have the same wavelength, and same dissipation-scale length, which we show below. Hence, Figure 3, column a shows results where the z-directed wavelengths (λ_z) for both modes have been reset to 5×10^8 m, the z-directed dissipation scale lengths (l_{dz}) have been reset to ∞, and the y-components of electric field (E_y) have been rescaled to make the wave-Pedersen conductivity equal the zero-frequency Pedersen conductivity. The starting point for these modifications is the modes found for a 100 km transverse wavelength and plasma density of 4.7×10^9 m^{-3} (at all altitudes), and the remaining modal properties are not modified.

The green curves of Panel a1, Figure 3, show the real (solid) and imaginary (dashed) parts of the downward-looking input admittance ($\Sigma(z)$) found by the model using the modified modes, plotted versus altitude, and the electrostatic result is shown in the dashed blue curve (it is purely real). It is seen that the model exactly reproduces the electrostatic conductance when the waves have these modified properties, and panel a2 shows that E_y maps unchanged through the ionosphere. Note that these results apply regardless of the starting point for the artificial modifications, and this has been tested for all four of the examples described in Section 7.2.

Panel a3 shows the modal contributions to E_y, and it is seen that the electric field is mostly carried by the Alfvén wave, except in the low-altitude region where the waves (polarization vectors) come into near alignment. The degree of alignment is shown in panel a4, in terms of the complex-conjugate dot-product between the polarization vectors for the waves. The resultant E_y remains smooth and unchanged, even though there is substantial mode-mixing and a kinky feature in the modal allotment associated with the polarization vectors rapidly coming into alignment.

Column b of Figure 3 shows the results when a significant amount of dissipation is added to the case of column a, with the wave-Pedersen conductivity maintained at the electrostatic value. The conductance now matches the electrostatic results only when seen from low altitudes, and decreases as the look-altitude moves away from the conducting region. The conducting region is becoming disconnected from the altitudes above, as the electric field becomes shielded.

Column c of Figure 3 shows the results when the wavelengths for both modes are shortened to 7×10^6 m, with the wave-Pedersen conductivities maintained at the electrostatic value, and without dissipation. There is now a resonance with a tangent function signature analogous to that discussed in Section 3, equation (5), for a uniform transmission line. We will find a similar effect for the real case discussed in Section 7.2. However, the effect seen in column c is very extreme, because the dissipation-scale-length has been set to ∞. In the real case the slight dissipation rounds-off the peaks of the tangent function, so that the effect looks much less extreme.

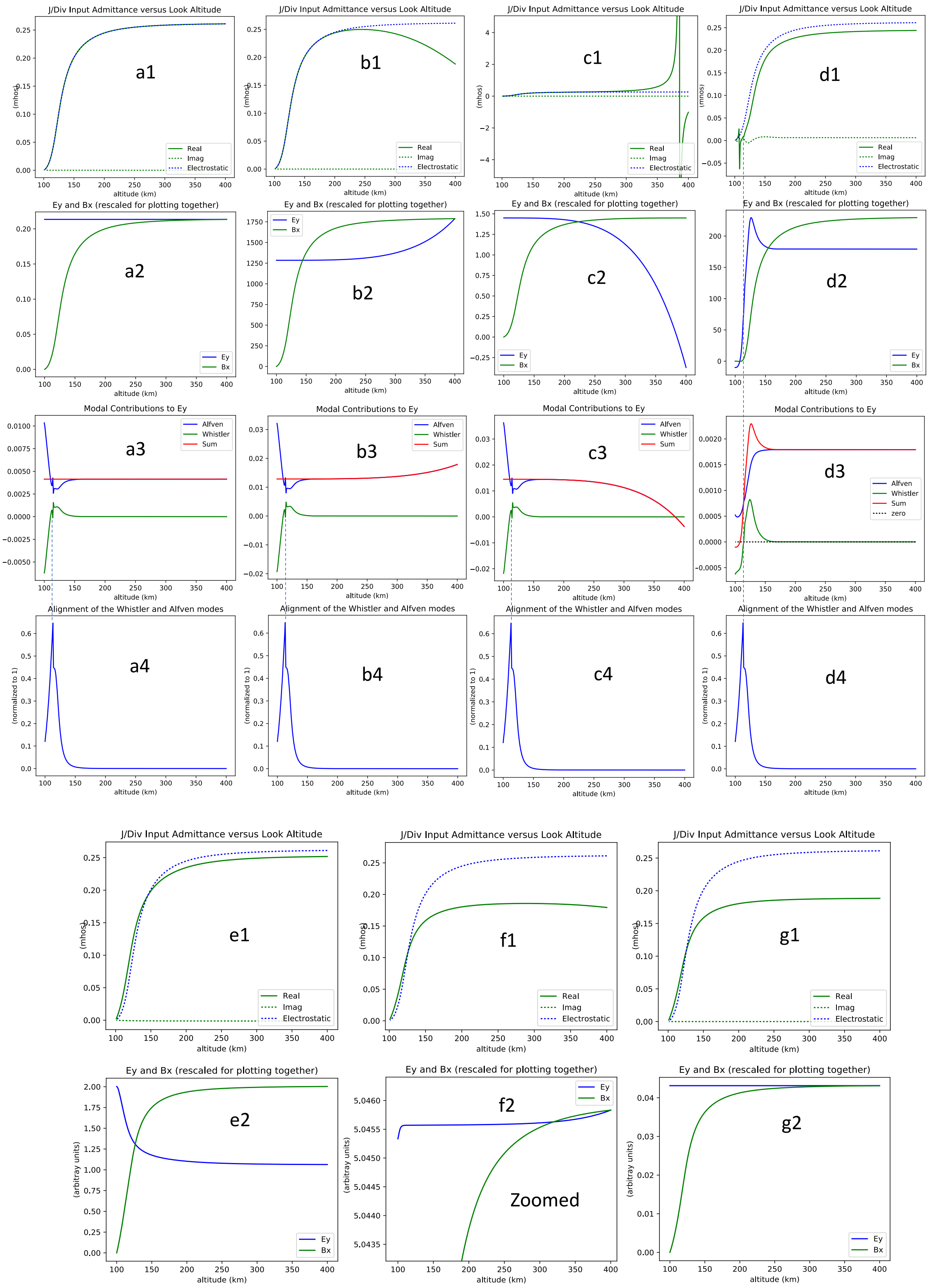

Figure 3: Model results using artificial waves that reproduce electrostatic theory, and show how deviations arise ($\lambda_\perp = 100\,\mathrm{km}$, $n_e = 4.7 \times 10^9 \mathrm{m}^{-3}$). Column a shows exact agreement with electrostatic theory. Column b shows what happens when dissipation is added to the case of column a. Column c shows what happens when the wavelengths of both modes are shortened from the case of column a. Column d shows what happens when the wavelength of the Alfvén mode only is increased from the case of column a, so that the wavelengths become unequal. Column e shows results using the real wave properties, but with only the Alfvén mode included in the model. Column f shows what happens when the wavelength is artificially lengthened from the case of column e, with E_y rescaled to maintain the same wave-Pedersen conductivity. Column g shows what happens when dissipation is (also) omitted from the case of column f.

Column d of Figure 3 shows the results when the wavelength of the Alfvén wave only is increased by a factor of 2.4 from the case of column a (to 12×10^8 m), with the wave-Pedersen conductivity maintained at the electrostatic value by rescaling E_y. Otherwise the parameters are the same as in column (a). Although the single-wave formulation of Section 3 finds that increasing the wavelength should reduce the wavelike effects, we find something very different in the bi-modal model. Making the wavelengths unequal has introduced what looks like a low-altitude resonance, even though the wavelengths are both too long to explain it, as demonstrated by the results in column a. There is also a little kink in the admittance curve, at a slightly higher altitude, and it looks like the electric field is cutoff right at the kink. Both the kink and the cutoff of the electric field seem to line-up with the sharp increase in alignment of the modes seen in panel d4. A vertical dashed line has been added to demonstrate the alignment. It looks like the matrix H_5 is becoming degenerate or nearly-degenerate right at his altitude. The conductance seen from above the altitude of the kink seems to reflect only the portion of the ionosphere that is above the kink. These results portend the real bi-modal model results presented below.

To understand what is causing these effects consider replacing the multi-section TL model with a coarse, two section model, where the boundary between the sections is right where the modal alignment rapidly increases (row 4 of Figure 3). Unlike in the upper section, in the lower section the polarization vectors of the two waves are very similar. The near lack of linear independence will create a situation where meeting the boundary conditions requires large and oppositely signed modal coefficients in the lower section, such that the two large waves are subtracted from each other to obtain a much smaller resultant. Hence, the signal propagating in the lower section will have the form $u_i = \left((N+\epsilon)\left\{E_{1x}, E_{1y}, B_{1x}, B_{1z}\right\} \mathrm{e}^{ik_1 z} - N\left\{E_{2x}, E_{2y}, B_{2x}, B_{2z}\right\} \mathrm{e}^{ik_2 z}\right)$, where for example $|N| \gg 1$ and $|\epsilon| \sim 1$ (although we do not use these relations). There will also be an oppositely directed signal with the same form other than having negative signs in the exponential and in front of B_{1x} and B_{2x}, but for simplicity we will ignore this component. We would like to compare the z-derivatives of the fields with those that would be obtained in the case of a single propagation mode with the same E_y, such as a signal of the form $u_i' = C\left\{E_{1x}, E_{1y}, B_{1x}, B_{1z}\right\} \mathrm{e}^{ik_1 z}$. We now solve for the ϵ that makes the bi-modal case have the same E_y as the single-mode case (i.e., $E_y = E_y'$ at location z) and evaluate the z-derivatives. After some algebra we obtain,

$$
\begin{aligned}
\frac{\mathrm{d}E_y/\mathrm{d}z}{\mathrm{d}E_y'/\mathrm{d}z} &= 1 + \frac{NE_{2y}}{E_y}\mathrm{e}^{ik_2 z}\left(1-\frac{k_2}{k_1}\right) \simeq 1 + \frac{NE_{2y}}{E_y}\left(1-\frac{k_2}{k_1}\right), \\
\frac{\mathrm{d}B_x/\mathrm{d}z}{\mathrm{d}B_x'/\mathrm{d}z} &= 1 + \frac{NB_{2x}}{B_x'}\mathrm{e}^{ik_2 z}\left(1-\frac{k_2}{k_1}\right) \simeq 1 + \frac{NB_{2x}}{B_x'}\left(1-\frac{k_2}{k_1}\right), \\
\frac{B_x}{B_x'} &= 1 + \frac{NE_{2y}}{E_y}\mathrm{e}^{ik_2 z}\left(1-\frac{Y_2}{Y_1}\right) \simeq 1 + \frac{NE_{2y}}{E_y}\left(1-\frac{Y_2}{Y_1}\right),
\end{aligned} \tag{21}
$$

where Y_1 and Y_2 are the wave admittances for the first and second modes, respectively, and we have also shown results for the case where $k_2 z$ is small. Because N may be a very large (complex) number, these results show that the z-derivatives may be much larger for the bi-modal case. Therefore, the bi-modal case may exhibit variations over a much shorter scale, and may exhibit wave-effects over this short scale that are not possible for the single mode case. This will happen when the modes are combined with large amplitudes, which we expect in the low-altitude region where they are strongly aligned.

On the other hand, again from equation (21), when the two modes have the same k (same wavelength and dissipation scale) the z-derivatives become identical to the single mode case, and if they also have the same wave-admittances then $B_x = B_x'$. So in this case the bi-modal model should give exactly the same results as the single-mode model with respect to E_y and B_x, and hence the same results for the input admittance (which ignoring the displacement current is $B_x/(\mu_0 E_y)$, since $k_x = 0$). The difference is that the bi-modal model will properly enforce the boundary conditions for E_x and B_z, and so these quantities may come out very different from those obtained using the single mode model. And there may be quite a lot of mode mixing required to do this, such as is seen in row 3 of Figure 3, where there are also kinks that all line up with the alignment-peaks seen in row 4. In fact, running the model for either of the (similarly-modified) single modes reproduces exactly the results seen in panels a1, a2, b1, b2, c1, and c2 of Figure 3, and this is strong evidence that the numerical accuracy in the bi-modal model-evaluation is sufficient to correctly determine the input admittance that is our main goal in this work.

Although there is a lot going on in the bi-modal model, this is a simple reflection of the fact that it contains

a lot of physics. Hopefully, the above synthetic examples will be enough to convince the reader that the model is functioning as intended, with sufficient numerical accuracy, and that it would reproduce electrostatic theory if the waves had the right characteristics. That the waves do not have the right characteristics is a simple result of finding the eigenvectors and eigenvalues of the electromagnetic five-moment fluid equations. In this regard the wave-Pedersen conductivity is the single most descriptive parameter, which governs the resultant input conductance when wave effects are negligible. And when wave effects are not negligible the electrostatic theory should not have any hope of working.

7.2 Real Results at the Four Corners of the Modeling Domain

We now begin presenting results using the real wave characteristics, starting with a single-mode example that provides our best match to electrostatic theory. Hence, consider the case where only the Alfvén mode is used in the model, and use the real wave characteristics for a 100 km transverse wavelength, with the rather low plasma density of 4.7×10^9 m^{-3} at all altitudes. The green curves in panel e1 of Figure 3 show $\Sigma(z)$, and the dashed blue curve shows the electrostatic result. The agreement is within 10%, which is confusing because in Section 3 we said that the Alfvén wave-Pedersen conductivity is too small to reproduce electrostatic theory. However, the agreement starts to breakdown when we look at panel e2, which shows E_y and B_x, and reveals that E_y does not map unchanged through the ionosphere. In fact, E_y increases with decreasing altitude. To understand this we try resetting λ_z to a larger value, while rescaling E_y in the polarization vector so that the wave-Pedersen conductivity is not changed. The results are shown in panels f1 and f2, which show that $\Sigma(z)$ has been greatly reduced, and that E_y now maps almost unchanged through the ionosphere (note the much finer scale). If we also set l_{dz} to ∞ the results in panels g1 and g2 are obtained, where E_y now maps perfectly through the ionosphere, and the slight droop is removed from $\Sigma(z)$ (E_y is again rescaled to maintain the same wave-Pedersen conductivity). The results now have the character expected from electrostatic theory, but $\Sigma(z)$ does not quantitatively agree with the electrostatic result. This shows that the near agreement seen in panel e1 is something of a coincidence, which would not occur if it were not for wavelike effects. Generally the wavelike effects create a much more interesting behavior, as we see now in exploring the rest of the results.

We now present the results from the one- and two-mode models covering a transverse scale size from 100 km to 1000 km, and covering a plasma density range from 4.7×10^9 m^{-3} to 1.0×10^{11} m^{-3}. Of course we expect the two-mode model results to be the physical results, but it is useful to compare the single-mode results in order to evaluate the effects of mode mixing. Figure 4 shows results for a 100 km transverse wavelength, where the upper six panels are for plasma density 4.7×10^9 m^{-3}, and the lower six panels are for plasma density 1.0×10^{11} m^{-3}. Panels a1 and d1 compare $\Sigma(z)$ (real part only) from the single mode models with the electrostatic results, for the respective densities. Because the Whistler mode is cutoff at higher altitudes, by itself it only provides for a non-zero conductance at look-altitudes that are quite low. Panels b1 and e1 show that the electric field would not map into the ionosphere if only the Whistler mode existed. Comparing panels a1 and d1 shows that the higher plasma density shortens the effective wavelength of the Alfvén mode so that it resonates, and that this resonance has nothing to do with mode-mixing (since these are single-mode models). This is the kind of tangent-function resonance that was discussed in Section 3, exemplified in column c of Figure 3, and predicted by the simple phase-rotation estimate of Figure 2, panel j. However, the lossiness that we noted in Section 6.2 for the Alfvén mode at the very bottom of the E region appears to almost completely eliminate the lossless-case infinities of the tangent function, for this single-mode model.

Panels a2 and d2 of Figure 4 compare $\Sigma(z)$ from the bi-modal model with the electrostatic results, for the respective densities 4.7×10^9 m^{-3} and 1.0×10^{11} m^{-3}. Besides the low-altitude spiky or kinky features that we will discuss later (mostly in Section 7.4), the bi-modal model gives results that are qualitatively similar to the model using only the Alfvén wave (compare panels a1 and d1, green curves). For the low-density case the quantitative results are also quite similar. However, for the higher density case the bi-modal model has a more distinct resonance effect, with larger overall swings that are more reminiscent of the expected tangent function behavior. This suggests that the presence of the Whistler wave has reduced the amount of energy in the Alfvén wave, in the very low altitude region where the latter becomes lossy. Finally, similar to the effects seen above in column d of Figure 3 (for the artificial waves with different wavelengths), panels c2 and f2 show that E_y experiences a sharp low-altitude peak, and then is rapidly cutoff before reaching the bottom of the ionosphere. Comparing panels c1 and f1 show that there is a similar effect when only the Alfvén wave

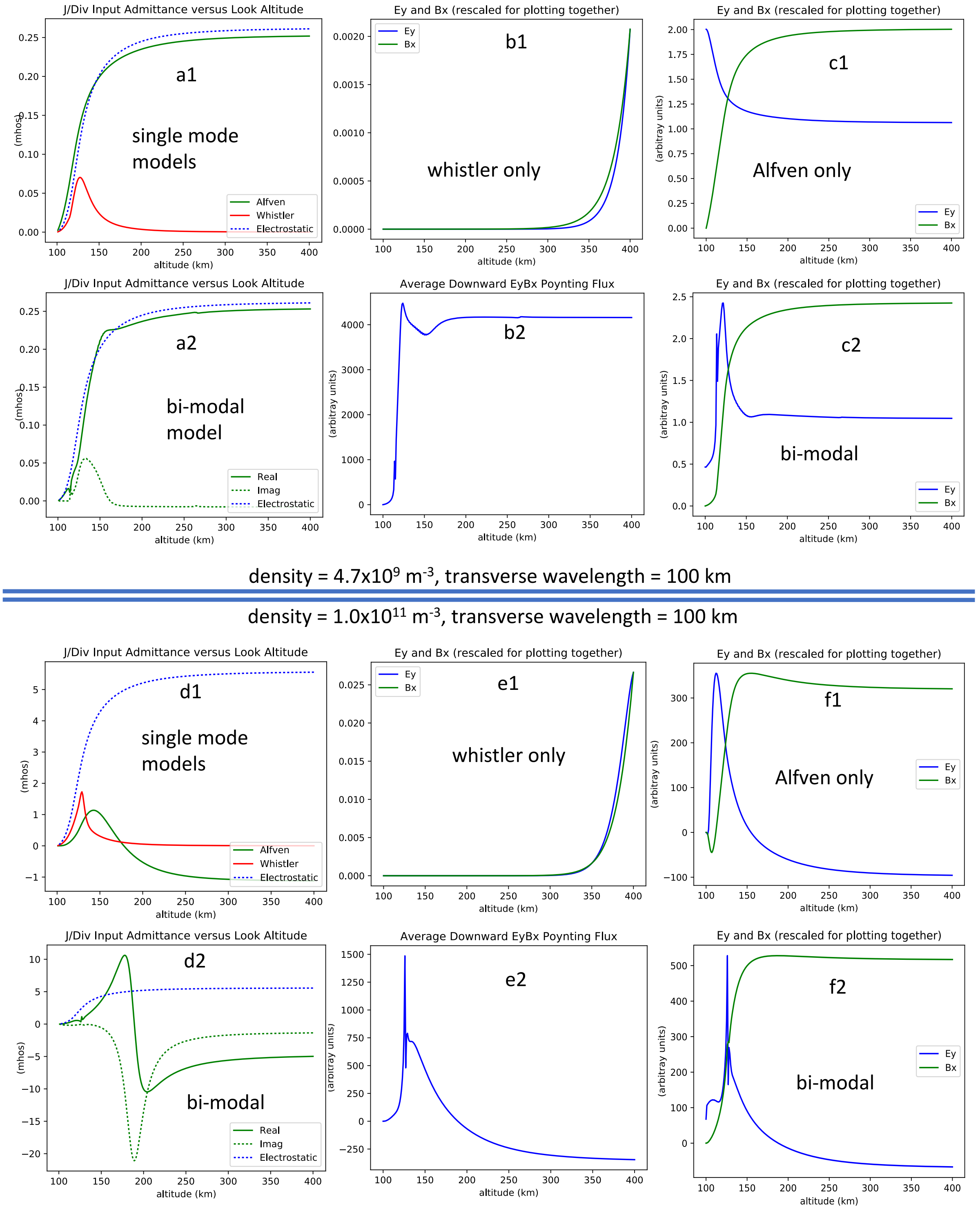

Figure 4: Single and bi-modal model results for a 100 km transverse wavelength, and for plasma densities of 4.7×10^9 m^{-3} (top) and 1.0×10^{11} m^{-3} (bottom).

is used in the model, but that the sharp cutoff is missing or not as sharp.

Turning now to the case of the 1000 km transverse wavelength shown in Figure 5, we find that the single-mode-resonance (tangent function of equation 5) is no longer present. However, E_y still does not map to the bottom of the ionosphere for any of the cases, and the bi-modal conductances at the top of the ionosphere are only 16% and 27% of the electrostatic predictions, for densities of 1×10^{11} and 4.7×10^9, respectively. For the Alfvén-mode-only cases (panels c1 and f1), although we had postulated in the pre-modeling analysis of Section 6.2 that dissipation might keep the electric field from mapping, artificially increasing the dissipation-scale-length does not make a significant difference. (This is an example of the lack of low-altitude sensitivity that has been referenced above, in support of the validation.) Hence the electric field cutoff in the Alfvén-mode-only cases must be due mostly to a mismatch caused by rapid changes in the Alfvén mode admittance quantities, in the lower E region (e.g., panel d of Figure 6, discussed below). It was also postulated in the pre-modeling analysis that the Whistler wave might prevent this effect and allow the electric field to map, but in fact the bi-modal results in panels c2 and f2 show that instead, E_y is cutoff at an even higher altitude. This is also likely due to a mismatch of some kind, and Section 7.3 is dedicated to analyzing this effect, with additional relevant discussion in Section 7.4.

Panels b2 and e2 in both Figures 4 and 5 show the $E_y \delta B_x$ component of the time-averaged downward Poynting flux for the bi-modal case, which is the component associated with the field aligned current and $\Sigma(z)$. (Note that continuity of δB_y is not enforced and so we will avoid trying to analyze the $E_x \delta B_y$ component, which anyway is not directly related to the field aligned current or $\Sigma(z)$, since $k_x = 0$ ($j_\parallel = \hat{z} \cdot \vec{k} \times \delta\vec{B}/\mu_0 = -k_y \delta B_x/\mu_0$).) The time-averaged Poynting flux is the real part of the Poynting vector, and so is not exactly the same as the product of the instantaneous E_y and δB_x that are shown in the other figures (which were taken at the instant when the downward Poynting flux maximizes at 400 km). In all four cases the Poynting flux experiences a sharp cutoff, such that the lower altitude portion of the ionosphere does not participate in closing the field aligned current, and does not contribute to the conductance seen at the top. The cutoff altitude increases with both density and wavelength, which produces a dependence that is very different from that expected under electrostatic theory. This same behavior exists for the Alfvén-mode-only models, with little change when the dissipation scale length is artificially increased. These findings all suggest that modeling errors at the bottom of the ionosphere will have little effect, and thus lend support to the validation given in Section 4. Hence, we find that the conductance seen at the top of the ionosphere is generally much less than expected under electrostatic theory. The reduction is very significant for both the bi-modal and Alfvén-only models, but is more pronounced in the bi-modal case.

7.3 Analysis of Sharp Cutoff of Electric Field

We have found that there is a blockage of the signal penetration that arises in both the Alfvén-only and bi-modal models, but which is more pronounced in the bi-modal case. For the Alfvén-only case we attribute this effect to wave-reflection caused by a sharp gradient in the Alfvén admittance, which gradient can be seen in panel d of Figure 6, for the case of a 1000 km transverse wavelength and density of $4.7 \times 10^9\,\mathrm{m}^{-3}$. In the pre-modeling analysis of Section 6.2 we had postulated that dissipation might prevent the signal from penetrating, but since artificially increasing the dissipation-scale-length does not significantly alleviate the blocking effect, it must instead be due to wave-reflection. While this is fairly easy to understand, what is harder to understand is the extremely sharp cutoff of the electric field that happens in the bi-modal cases, and beyond which the downward Poynting flux is essentially zero. These locations also seem to be associated with spiky or kinky features, which we will discuss separately in Section 7.4, and also in the last paragraph of this section.

To analyze the cutoff we will focus on the case shown in panels a2, b2, and c2 of Figure 5, where the effect is both clear and relatively simple in form. A closer examination of this case is shown in Figure 6, panels a-f, which show from top to bottom the input admittance, E_y and B_x, the parallel wavelengths, the magnitude of the wave admittances, the alignment of the modes, and a quantity we call the "mode-mixing coherence length" to be described below. All these quantities are plotted versus altitude, and a red-dashed vertical line is drawn to indicate the altitude of electric field cutoff. We will attempt to interpret the cutoff as a wave reflection phenomenon that is enhanced by the presence of more than one wave mode.

Panel d of Figure 6 shows that over most of the ionosphere the Alfvén admittance is relatively constant, and so the signal essentially propagates in a uniform transmission line all the way down to about 134 km

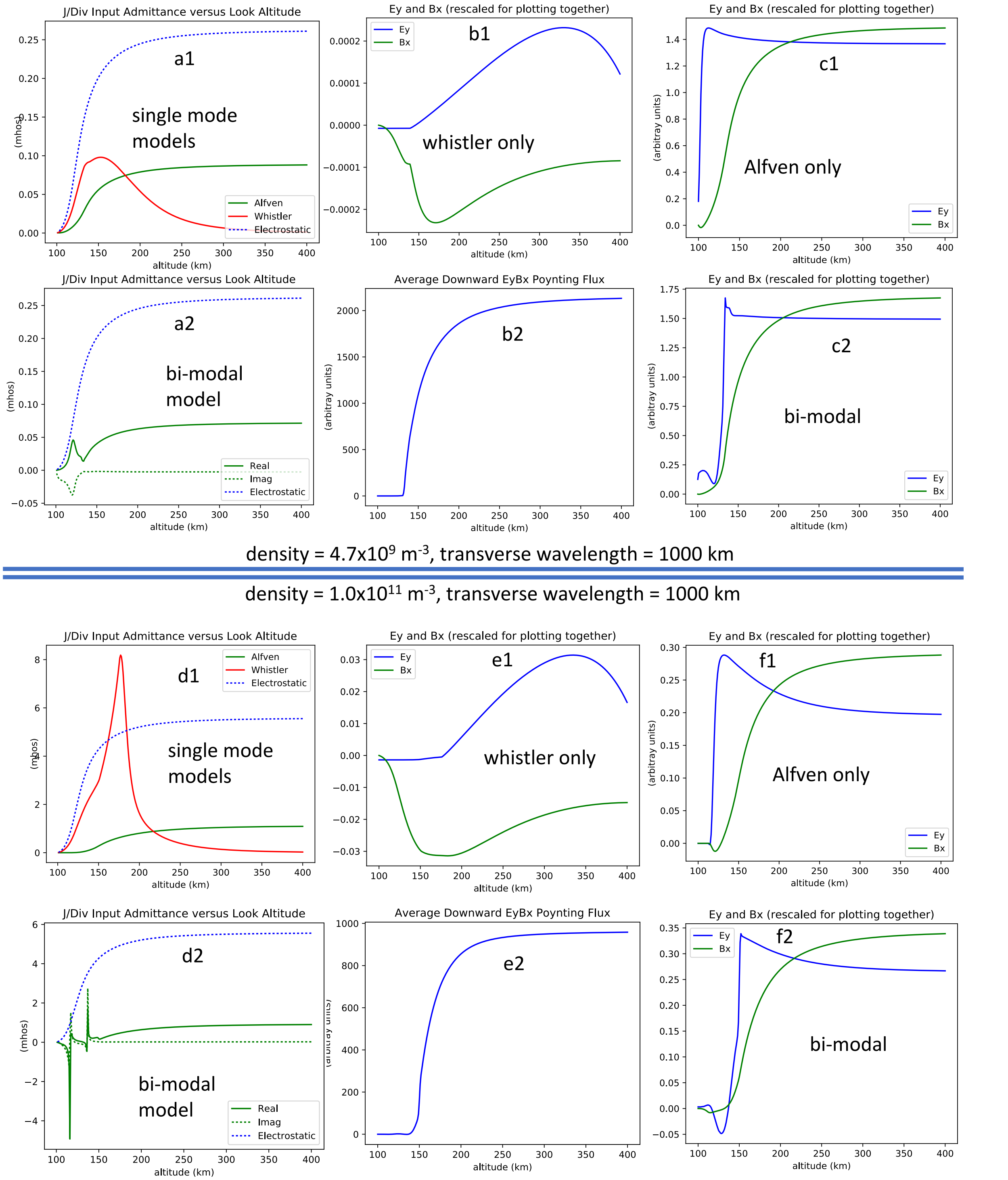

Figure 5: Single and bi-modal model results for a 1000 km transverse wavelength, and for plasma densities of $4.7 \times 10^9\ \mathrm{m}^{-3}$ (top) and $1.0 \times 10^{11}\ \mathrm{m}^{-3}$ (bottom).

in altitude. However, at 134 km the Whistler mode admittance becomes equal to the Alfvén admittance and the two modes very-suddenly become strongly aligned. This location also corresponds to a break in the parallel wavelength of the Whistler mode, right where that wavelength becomes nearly equal to that of the Alfvén mode. It appears that the Whistler mode changes its physical wave-type at that altitude, and that this change is associated with a degeneracy or near-degeneracy of the matrix H_5, as was already mentioned in Section 6.2. The rapidly changing alignment and crossing wave-admittances suggest that this could be a point of strong reflection for the Alfvén mode, which might prevent energy from propagating further downward.

However, the reflection appears to be very sharp, and this is something of a puzzle considering the wavelengths are not all that short for either mode. The wavelength plays a role something like a correlation length, which limits the scale over which the signal can change. When two scatterers are located much closer together than a wavelength there cannot be much change between them, and so they are effectively colocated. Yet in Figure 6 the electric field changes dramatically between the bottom of the ionosphere and the apparent scattering location at 134 km. These two scattering locations appear to be behaving independently, as though the wavelength were shorter than what is shown in panel c of Figure 6. Below 134 km the Alfvén wavelength shortens dramatically, and so this conundrum does not arise for the Alfvén-mode-only case where the scattering occurs at a lower altitude and is less sharp. But how can we have such sharp scattering at 134 km?

To resolve this doubt we refer back to equation (21) and the surrounding discussion. Since the z-derivative of $E'(z)$ is $ik_1E'(z)$ and we have set $E(z) = E'(z)$, we can define something like an effective k for the mode-mixed case by,

$$\frac{\mathrm{d}E_y/\mathrm{d}z}{\mathrm{d}E'_y/\mathrm{d}z} = \frac{k_{MM}}{k_1}.$$

Since we do not actually expect oscillation with wavenumber k_{MM}, we regard this more as a way to estimate a correlation length for the mixed-mode case. Hence in panel f we use equation (21) to plot $2\pi/k_{MM}$, which we consider as a correlation length for use in estimating the degree to which the scatterer at 134 km can be considered as independent from the scattering off the bottom of the ionosphere. (We note also that panel d prompted us to try an alternative, short-circuit boundary condition for the Whistler wave at the bottom of the ionosphere, and that this had almost no effect on the results.) Panel f shows that this correlation length becomes very short in the neighborhood of 134 km, less than 1 km at the location of the cutoff, and this can viewed as an explanation for why the scatter located there produces such sharp and apparently independent scattering. The two modes together are acting like a wave with much shorter wavelength, and this can be understood from equation (21).

In all the cases the alignment of the cutoff seems to be consistent with this interpretation. And it is also consistent that for the artificial bi-modal cases in Figure 3, there is no hint of a cutoff until the wavelengths are made unequal (column d). With respect to the kinky features, columns a-d of Figure 3 all show kinks in the modal allotment, but there is no hint of an electric-field cutoff until the wavelengths are made unequal (column d).

However, at altitudes below the cutoff there sometimes occurs another feature that looks more like a resonance, that is, a place where the electric field is nearly canceled. There are two such prominent features in the admittance function for the case of a 1000 km transverse wavelength with density $1.0 \times 10^{11}\,\mathrm{m}^{-3}$, seen in panel d2 of Figure 5. There is also an example in the artificial case shown in column d of Figure 3. Although dramatic in appearance, it seems that these features are incidental to the scattering that occurs at the positions of the cutoffs. In panels g-j of Figure 6 are shown the separate contributions to E_y from the two modes, where from top to bottom appear the cases with transverse-wavelengths and densities of $\left(100\,\mathrm{km}, 4.7 \times 10^{9}\,\mathrm{m}^{-3}\right)$, $\left(100\,\mathrm{km}, 1.0 \times 10^{11}\,\mathrm{m}^{-3}\right)$, $\left(1000\,\mathrm{km}, 4.7 \times 10^{9}\,\mathrm{m}^{-3}\right)$, and $\left(1000\,\mathrm{km}, 1.0 \times 10^{11}\,\mathrm{m}^{-3}\right)$. Only the bottom-most of these examples exhibits the resonant-like effects. However, the bottom three panels all show very similar behavior for the Alfvén- and Whistler-mode contributions; the wiggles and curves appear to be driven by the same physics. The only real difference to be associated with the bottom panel is that the vertical positioning of the wiggles and curves is such that the resultant E_y crosses the z-axis (i.e., passes through zero). There is no dramatic effect to be seen in E_y, yet because E_y is in the denominator of the admittance function the latter does display a dramatic effect. The upper panel (panel g) is the exception in that it does not display the same wiggles and curves, but it has the lowest altitude cutoff of the four, and so there may simply not be room for those phenomena to occur before

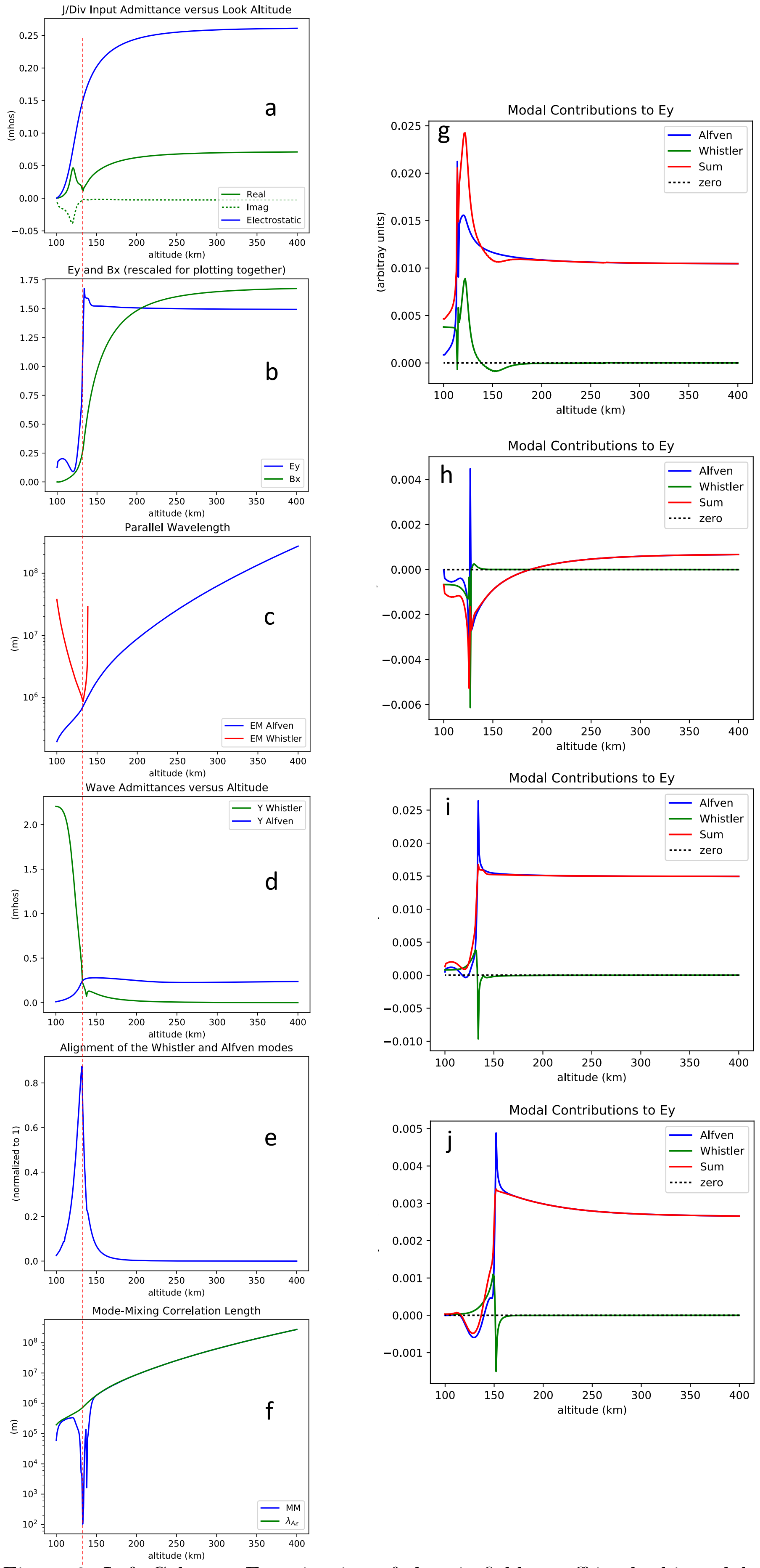

Figure 6: Left Column: Examination of electric field cutoff in the bi-modal model. Right Column: Modal contributions to E_y [panel g, ($\lambda_\perp = 100\,\mathrm{km}, n_e = 4.7 \times 10^9 \mathrm{m}^{-3}$); panel h, ($\lambda_\perp = 100\,\mathrm{km}, n_e = 1.0 \times 10^{11} \mathrm{m}^{-3}$); panel i, ($\lambda_\perp = 1000\,\mathrm{km}$, $n_e = 4.7 \times 10^9 \mathrm{m}^{-3}$); panel j, ($\lambda_\perp = 1000\,\mathrm{km}$, $n_e = 1.0 \times 10^{11} \mathrm{m}^{-3}$)].

meeting with the bottom of the ionosphere. It is the well known feature of admittance functions that they may include zeros in the denominator, such as in the case of the tangent function in equation (5). Hence, there is no anomaly in these findings.

7.4 Modeling Limitations and Quantitative Results for Wavelength and Density Dependence

In this section we provide a summary of the results over the modeled region, which is bounded by the four corners discussed in Section 7.2. However, before doing this there is one caveat to report concerning the modal resolution in the vicinity of the degeneracy, which affects the quantitative results. Panel a of Figure 7 shows the real part of the input admittance (the conductance) plotted versus look altitude for a 100 km transverse wavelength and 11 different plasma densities between $4.7 \times 10^9\,\mathrm{m}^{-3}$ and $1.0 \times 10^{11}\,\mathrm{m}^{-3}$. The curves are normalized to the electrostatic results for the respective density, which are thus shown by the single dashed black line. The gradual ascent to the tangent function signature is apparent in panel a. However, also seen in panel a is a very sharp feature colocated with the cutoff of the electric field and the degeneracy of the matrix H_5.

Resolution Limitations: Although the cutoff of the electric field certainly seems to be a real prediction of the model (Section 7.3), there are sometimes other kinds of sharp features that also occur right at the location of the degeneracy, and these are more difficult to assess. (The sharp features occurring *below* the degeneracy were discussed at the end of Section 7.3.) The sharp feature in the admittance function seen in panel a of Figure 7 is associated with a downward spike in the electric field (not shown), and similar electric-field spikes are seen in all the bi-modal cases with a 100 km transverse wavelength (e.g., panels c2 and f2 of Figure 4, where these plots may not reveal the full depth of the spike, since the points are spaced farther apart [1 km] than in Figure 7). It appears that these electric-field spikes can sometimes pass very close to zero in the complex plain, giving rise to even sharper spikes in the admittance function, since the electric field is the denominator of admittance. Exactly how close to zero would seem to be a highly sensitive thing to predict, and so the spikes in the admittance function are probably not accurately calculated. But as for the prediction of the downward spikes in the electric field, it seems likely that they are real predictions. However, our ability to probe very close to the degeneracy is limited by resolution, and not only does this affect assessment of these electric-field spikes but it also sometimes affects the quantitative results for the admittance seen at the top of the ionosphere. Hence, we need to discuss the resolution limitations for the calculation.

The calculation resolves in the Fourier domain, and so resolution in this case means resolution in $\vec{k}$-space. As discussed in Section 2, for a Fourier domain model there is continuous spatial resolution, in this case by the analytic functions (14) and (B.3). So since we are solving for one Fourier component at a time, the resolution would seem to be a question that only affects future applications, when we try to assemble many Fourier components to create a 3D spatial-domain representation. Except, we do have the issue that we are stacking TL sections vertically, and so there does seem to be some kind of spatial resolution issue. How should we understand this?

In an idealized case where the wave properties are constant with altitude, we could represent the whole ionosphere by a single TL section. This provides continuous vertical resolution, even though the “sampling” is very coarse (i.e., a single TL section). And similarly, when there *is* variation in the wave properties (like admittance variations or wavelength variations) it should be sufficient to refine the sampling (i.e., use shorter TL sections) only to the extent that the variation in wave properties is well resolved; beyond this, using shorter TL sections should have no effect. That is, we do not need to resolve the electric field variation with the sampling, since that variation occurs within the TL sections, not across the boundaries as would be the case for a spatial domain model.

However, for the cases where the resonance is in evidence (e.g., Figure 4, bottom panels) making the sampling finer does make some difference in the admittance seen at the top of the ionosphere, and it does not seem possible to achieve a completely stable limit. The resonance certainly does not go away, but the zero crossing moves a little, and the conductance swing changes in magnitude. This was at first confusing because provided that the sampling is sufficient to resolve the variations in the wave properties, making the sampling finer should not have an effect.

The explanation seems to be that when the modes are nearly degenerate (i.e., nearly the same), the

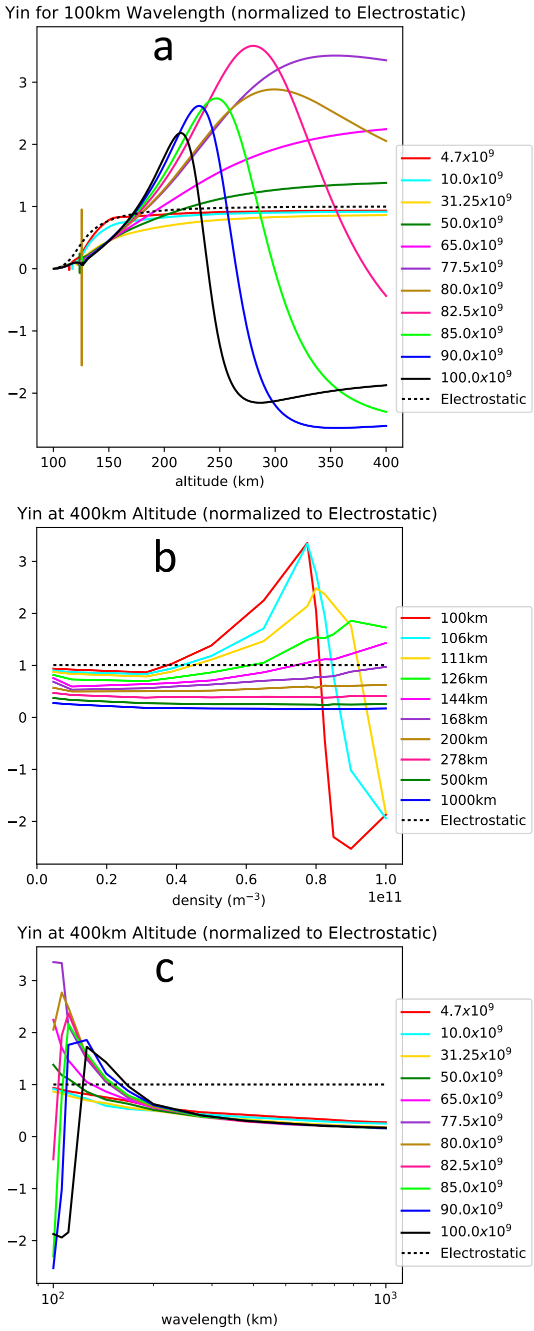

Figure 7: Input admittance versus altitude (panel a), density (panel b), and wavelength (panel c), normalized to the field-line-integrated conductivity (dashed black lines). The transverse wavelength for panel a is 100 km. The legends for panels a and c show the density in m^{-3} (constant with altitude). The legend for panel b shows the transverse wavelength. The curves in panels b and c are made up of end-points of curves like the ones in panel a, and as such have a higher level of model uncertainty.

modal resolution is not sufficient to properly differentiate them. We have tested the accuracy of the eigenvectors/eigenvalues by multiplying the eigenvector by H_5, and comparing the result to the eigenvector multiplied by the eigenvalue. Doing this generally shows agreement to 14 or 15 decimal places, which should be plenty sufficient. But there is another issue, which is that we find the k_z for each TL section by adjusting it until the real part of ω matches the source frequency (ω_0) within tolerances, which are currently set at 1%. So there is actually error associated with this tolerance. The error is not associated with finding the eigenvector/eigenvalue, rather it is associated with finding the *right* eigenvector/eigenvalue, that is, the ones associated with the frequency of the source. While the 1% tolerance seems to be sufficient for resolving the vertical variation in the wave properties, it may not be sufficient to properly differentiate the Whistler and Alfvén modes when they become nearly degenerate.

To understand the effect of this, go back to our discussion of equation (21), which shows that the vertical derivative (derivative with respect to altitude) increases with the amplitude N of the modes. As explained, the amplitude will generally increase when the modes come closer into alignment, and will actually tend to infinity if the modes approach perfect alignment (become degenerate). So when the modes are nearly aligned, the error associated with the 1% tolerance could produce a large error in their amplitudes, and thus in the vertical derivative. In refining the altitude sampling we explore closer to the degeneracy where the modes are nearly aligned, and at some point the modal resolution will become comparable to the separation between the modes, which will introduce numerical error into the vertical derivative. If the sampling is greatly over-refined the error could dominate and produce artificial spiky structures over very small scales.

However, in exploring different altitude samplings, we find a remarkable degree of robustness in both the cutoff of the electric field and the electric-field spikes seen in panels c2 and f2 of Figure 4. In the particular example of a 100 km transverse wavelength with density $4.7 \times 10^9\,\mathrm{m}^{-3}$, when the sampling is increased from a few tenths of a kilometer to as much as 4 km, both the cutoff and the downward electric-field spike are found to persist with only modest change. Hence it would seem that both the cutoff and the spike are real predictions of the model. In particular, although over-refinement of the altitude sampling could produce artificial spikiness on small scales, the downward electric-field spikes seen in panels c2 and f2 of Figure 4 are not caused by this and appear to be real predictions of the model.

On the other hand, the spikes seen in the admittance function in panel a of Figure 7 are much less likely to be real. These admittance function spikes are produced when the before-mentioned electric-field spikes pass very close to zero in the complex plane, and so could be very sensitive to small changes in the electric-field spikes. If over-refinement of the sampling has created artificial, very-small-scale spiky-structures riding on top of the (apparently) real electric field spike, these could produce very sharp looking spikes in the admittance function that are also artificial. And it is certainly reasonable to worry that there could be other, more important effects. However, with the exception of the cases when the ionosphere is in resonance (like in the bottom panels of Figure 4), we find below that the conductance curves, and in particular the conductance seen at the top of the ionosphere, are not very sensitive to changes in the altitude sampling, as long as it is sufficiently dense.

When the sampling is increased to 8 km in the example just discussed, the cutoff of the electric field is lost and the trace reverts nearly to what is seen in panel c1 of Figure 4, which shows the case where only the Alfvén wave is used in the model. This tells us something about the other extreme, when the sampling is insufficient. Apparently, insufficient altitude sampling causes the effect of coupling into the Whistler wave to be lost. So while we are still learning about what level of sampling is "sufficiently dense," it is clearly important not to skip over the degeneracy entirely.

With regard to the spatial domain, finite difference models, there are a couple of related issues to mention here. First, it seems that these models will require very-fine altitude-sampling to resolve the effect of coupling into the Whistler wave. This is because the coarsest usable sampling of 4 km (in the example just discussed) actually represents something much finer than 4 km altitude *resolution*, since there is continuous resolution within each 4 km TL section. The second issue is that these temporal/spatial domain models make low-frequency approximations (see Section 2) that could affect the degeneracy itself. Even if the coupling effect were deemed sacrificable, the degeneracy seems to be responsible for the rapid variation in the Alfvén admittance that reflects the Alfvén mode, and generally leads to cutoff of the electric field even when the Whistler mode is not included in the calculation (Figures 4 and 5). So in order to replicate the electric field cutoff in any form using these temporal/spatial domain models, it will likely be important to make sure that the degeneracy is properly represented in the dispersion relation.

Mitigation Measures: For the cases where the tangent function behavior is in evidence, we have found that the end points of the conductance curves at 400 km suffer variations according to the particular sampling of the region around the degeneracy. There is certainly no doubt about the emergence of the tangent function behavior, since we found that behavior also in the Alfvén-mode-only model where there is no issue of modal resolution, and also no kinky or spiky features (see panel d1 of Figure 4), and also since in panel a of Figure 7 the behavior is seen to arise gradually and consistently with increasing density. But the current modal resolution does not seem to be sufficient to achieve quantitative accuracy for the tangent function signature.

Hence, in order to provide the quantitative results shown in panels a, b, and c of Figure 7, we have run the model for an ensemble of different altitude samplings and chosen the median result. In doing this we ignore the sharp features and choose the curve that is the median over the greatest fraction of the altitude region. In some cases the median curve has very sharp features in the admittance function, at the location of the degeneracy, and so it seems that these features do not represent any serious corruption of the calculation.

The ensemble curves are normally very tightly bunched, except when the electrical thickness becomes large and the tangent function signature begins to emerge. Since there are now plenty of reasons to feel certain about the emergence of the tangent function, and since the curves in panels f and g change continuously and consistently, we view this as a strictly quantitative limitation, which does not affect the qualitative conclusions. Thus, it may be that ionospheric behavior is sometimes very difficult to predict with quantitative accuracy. However, this does not mean that we cannot understand it. And, a better understanding of the degeneracy may be possible with additional research.

Summary of Quantitative Results: The conclusions are summarized in panels b, and c of Figure 7, which show the input conductance at 400 km plotted versus density and wavelength, respectively. The curves are again normalized to the electrostatic result for each density, which is therefore represented by the horizontal dashed black line at one. The panels represent two different ways of presenting the same results, and from either panel we can see that the (normalized) ionospheric conductance decreases with increasing wavelength, and has only a weak dependence on density when the transverse wavelength exceeds about 200 km (meaning that the un-normalized conductance has a nearly linear dependence on density). This long-wavelength conductance is much less than the electrostatic prediction (the field line integrated conductivity). At the other extreme, as the transverse wavelength decreases the electrical thickness increases, and it is necessary to refer to the end-points of the curves in panel a to understand the behavior. The electrostatic predictions appear to be pretty good when the density is low and the wavelength short, but this represents a quite limited region of parameter space, and we have also found above (see discussion of columns f and g of Figure 3) that it is somewhat fortuitous. Outside of this region the electromagnetic and electrostatic predictions are very different.

Implementation Challenges: The investigation has been limited to transverse scales between 100 km and 1000 km partly because there were difficulties evaluating the model outside of this range. Consistently sorting the modes is one of the most problematic aspects of model implementation, as it is difficult to devise an algorithm that works in all cases. The calculation optimizes the parallel wavelength (k_z) to the target operating frequency (ω_0), and a single failure of the mode sorting can cause the optimization to fail. These failures are easy to detect, but not so easy to fix. Our current sorting algorithm has been made to work over the range investigated, but occasionally fails outside of this range.

And there is also the difficulty with modal resolution that was already discussed, where ω_0 cannot always be matched with sufficient accuracy. In the wavelength regime greater than 1000 km it was sometimes not even possible to achieve the relatively-loose 1% tolerance employed above. The difficulty is that the real part of ω (ω_r) turns out to be very sensitive to k_z. The achievable accuracy appears to be limited by the precision of the representation of k_z, and by the precision of the arithmetic for finding ω_r from k_z, which is currently double precision. Thus in order to improve the modal resolution we need to use higher precision arithmetic, such as quadruple precision arithmetic. And also we need to understand whether the modes are actually becoming fully degenerate, in which case increasing the precision cannot fully solve the problem. In this case a theoretical solution would be needed to fully understand the signal propagation through the degeneracy. It will also help to use a higher frequency for ω_0 (i.e., higher transverse velocity), in situations where it is physically appropriate.

The good news is, it is really only necessary to do a one-time evaluation of the eigenmodes for a sufficiently fine sampling of transverse wavelength, density, transverse velocity, and altitude (i.e., collision frequency),

and thereafter the model can be run almost instantly for an arbitrary altitude profile and wavelength. Matching the boundary conditions does not appear to require any additional numerical precision. Hence, the model could eventually be made widely available for use on any computer.

8 Conclusions

In Section 3 we reframe selected results from *Cosgrove* [2016] using transmission line theory. When electromagnetic theory can be approximated using a single wave mode, then electrostatic theory (see Section 1 for disambiguation) is also applicable if the following three conditions hold: (1) the wave-Pedersen conductivity agrees with the zero-frequency Pedersen conductivity; (2) the wavelength is long compared to the system size; and (3) the dissipation scale length is long compared to the system size. With the exception of condition (3), it was found in *Cosgrove* [2016] that these conditions do not generally hold for ionospheric science. Moreover in Section 7.1 we find that when the system supports two wave modes that couple, then additional conditions should be added that are also not satisfied.

Therefore, in this work we develop an electromagnetic calculation of the ionospheric input-admittance based on transmission line theory. The real part of the input-admittance would normally be called the ionospheric conductance, which has been previously estimated as the field line integrated conductivity, based on electrostatic theory (such as the assumption that the electric field maps unchanged through the ionosphere). The electromagnetic calculation, which also provides the field quantities resolved over altitude, finds that the ionospheric response can be approximated as the superposition of two wave modes, which are quantified by the eigenvectors and eigenvalues of equations of motion derived from fluid theory. The major assumption used in the calculation is that highly lossy modes do not couple significantly into the dynamics, and so can be omitted. Especially, the mode often called the ion-acoustic wave, or alternatively the slow-mode MHD wave, is assumed not to couple into 100 km-1000 km transverse wavelength signals arriving from the magnetosphere (the range of scales analyzed herein). The ion-acoustic mode is found to have a dissipation scale less, and generally much less than 10 km at these scales. If this mode were to couple strongly into the dynamics it would prevent the signal (for example the electric field) from penetrating deeply into the ionosphere, and thus omitting this mode is required if there is to be any chance of reproducing electrostatic theory. A more legitimate reason for omitting this mode is found in the fact that the corresponding eigenvector is nearly orthogonal to those of the two modes chosen for inclusion, which we have called the Alfvén and Whistler modes, in analogy to the physically defined waves bearing those names.

Regarding the naming of the modes, it should be understood that we are equating eigenmodes (i.e., eigenvectors and eigenvalues) over the full altitude extent of the ionosphere (actually from 100 km to 400 km). Because the collision frequencies for ions and electrons change dramatically over this altitude range, the physical nature of our modes is not fixed (Section 6). The more common approach to defining wave modes is based on physical categorization, where simplifying assumptions are made in order to obtain a relatively simple dispersion relation (for example, the high-frequency assumption that ions are immobile). Hence there is not a one-to-one mapping between the modes that we define and the waves that are commonly considered in space physics. For example, in *Cosgrove* [2016] we described the reason for choosing the name "Whistler mode" and explained that it actually encompasses both the traditional Whistler wave and the fast-mode MHD wave, which are normally formed from high- and low-frequency approximations, respectively [e.g., *Stix*, 1992]. Our methods are not naturally conducive to a physical categorization of waves, and such a thing would be outside the scope of this work. Our focus here is on creating an electromagnetic calculation of the ionospheric conductance that can validate or replace the current electrostatic baseline (the field line integrated conductivity).

As an initial, validation step we show that the calculation would reproduce the electrostatic results exactly if the waves found from the fluid equations satisfied the conditions identified in the first paragraph of this section. In addition to reproduction of the field line integrated conductivity, the electric field is also seen to map unchanged through the ionosphere. These confirmations are made by artificially modifying the real waves, such that the conditions are satisfied. After this we move on to employing the actual wave properties determined from the electromagnetic five-moment fluid equations, and the results have also been cross-checked using the smaller equation-set from *Cosgrove* [2016].

The results found using the actual wave properties do not, for the most part, support the calculation of

ionospheric conductance as the field line integrated conductivity. For a low density ionosphere ($4.7\times10^{9}\,\mathrm{m}^{-3}$) with 100 km transverse wavelength we find a modest 7% reduction from the field line integrated conductivity. But increasing the density to $1.0\times10^{11}\,\mathrm{m}^{-3}$ produces a resonance associated with short parallel wavelength, such that the conductance becomes negative (energetically, this implies that non-electromagnetic forms of energy are important [*Vasyliunas and Song*, 2005; *Cosgrove*, 2016]). The resonance goes away when the transverse wavelength is increased to 1000 km, but in this case a severe mismatch arises in the E region that prevents the signal from penetrating deeply. There is a sharp cutoff of the electric field that would be very hard to resolve using a spatial-domain method. The mismatch is associated with a degeneracy or near-degeneracy of the equations of motion, and thus might also be sensitive to the approximations made in resistive MHD simulations. Together with the fact that the wave-Pedersen conductivity is less than the usual (zero-frequency) Pedersen conductivity, there is a result that the conductance is reduced from the field-line-integrated conductivity by 73% and 84%, for densities of $4.7\times10^{9}\,\mathrm{m}^{-3}$ and $1.0\times10^{11}\,\mathrm{m}^{-3}$, respectively. We have also verified that similar results are obtained when only the Alfvén wave is used in the calculation (which requires abandoning some of the physical boundary conditions), including the resonance and its associated negative conductance. A quantitative summary of the results is given in Figure 7, although it may be difficult to understand this figure without reading (at least) the whole of Section 7.

We also evaluate the practice of using electrostatic waves as approximations for electromagnetic waves in the collisional ionospheric plasma. By electrostatic waves we mean the practice of using $\vec{\nabla}\times\vec{E}=0$ in the Maxwell equations, but otherwise retaining the time dependence in the equations of motion, so that wave behavior is supported (see Section 1 for disambiguation). This simplification leads to replacing the Maxwell equations with the Poisson equation (Appendix A). We find that electrostatic waves may be good approximations for electromagnetic waves when the transverse wavelength is less than about 100 m. But above this scale we find significant differences, such as parallel wavelengths that are too long by orders of magnitude, and wave-admittances that are very different. And we could find no electrostatic counterpart for the Whistler wave, which we find to be an important contributor in the E-region ionosphere, where the wavelength and dissipation scale length for the Alfvén wave become very short. Hence, our overall finding is not just that a wave description is required, but that an electromagnetic wave description is required [*Vasyliunas*, 2012; *Cosgrove*, 2016].

We regard our form of calculation as the natural electromagnetic generalization of electrostatic theory, just as transmission line theory is used in electrical engineering to generalize electrostatic theory for circuits with dimensions comparable to the wavelength. Our method provides a direct, intuitive, and nearly analytical calculation of the ionospheric input admittance, which being a circuit quantity is defined only for a linear system in steady state. Steady state does not mean setting the time derivatives to zero in the equations of motion, rather it means allowing evolution to proceed until everything stops changing. As explained in Section 2, temporal/spatial-domain simulations are not well suited to this purpose. Hence, our method of calculation is complimentary to the temporal/spatial-domain approach, and is especially well suited to our goals, which are as much theoretical as they are modeling. Our results are of a rigorous, theoretical nature, while at the same time providing physical understanding. Thus they allow for evaluating whether complex numerical simulations based on "resistive MHD" are respecting the physical fundamentals.

If possible, it should be evaluated whether temporal/spatial-domain simulations are able to reproduce our results, and if not to understand where the difference is arising. However, there appear to be some serious obstacles, which have been discussed briefly in Sections 1 and 2. One of these is the implementation of the source as a boundary condition, which does not account for the back-reflected wave. Another is the importance of the degeneracy in the equations of motion, which requires very high vertical resolution, and may be sensitive to the simplifications that are usually made to eliminate the radio-frequency modes. To verify that the dispersion relation is adequate the equations of motion can be linearized and used in the TL model, to see if there is any difference from the results using the full dispersion relation. Although, we note that the TL method also has limitations with respect to the degeneracy, which arise from the limited modal resolution (Section 7.4). We hope that our results will stimulate others to study the problem, as it is really by comparing different methods that the most definitive results can be obtained.

The results are important in at least two different ways. The results at high densities and short wavelengths [i.e. ($\sim 10^{11}\,\mathrm{m}^{-3}$, $\sim 100\,\mathrm{km}$)] suggest unexpected behavior on the scale of auroral arcs, which scale also includes many other ionospheric phenomena, such as medium-scale traveling ionospheric disturbances [e.g., *Garcia et al.*, 2000; *Cosgrove and Tsunoda*, 2004], E region dynamos associated with sporadic E [e.g.,

Haldoupis et al., 1996; *Cosgrove and Tsunoda*, 2002; *Hysell et al.*, 2004], and equatorial spread F [e.g., *Tsunoda et al.*, 1982; *Aviero and Hysell*, 2012; *Tsunoda*, 2015]. The results at longer wavelengths affect global modeling of the magnetosphere, which generally uses the ionospheric conductance to establish an inner boundary condition [e.g., *Raeder et al.*, 1998; *Gombosi et al.*, 2001; *Toffoletto et al.*, 2003; *Lyon et al.*, 2004; *Janhunen et al.*, 2012; *Lotko et al.*, 2014]. Our results suggest that it may be important to, at a minimum, establish an effective scale for the magnetosphere-ionosphere interaction, and to use the conductance computed for this scale. Research on Poynting flux events could be used for this purpose [e.g., *Knipp et al.*, 2011; *Cosgrove et al.*, 2014; *Pakhotin et al.*, 2020; *Pakhotin et al.*, 2021]. The recent paper by *Pakhotin et al.* [2021] finds that half the Poynting flux is associated with transverse scales less than 250 km, and if this relatively short scale characterizes the magnetosphere-ionosphere interaction the results presented here are very relevant. And even if the effective scale is longer than our current upper-scale of 1000 km, the physical understanding derived herein does not give any reason to expect that electrostatic theory would become any more relevant.

This work has focused on developing modeling methodology and only a few initial findings could be communicated. There is a lot more to do, both in developing the calculation into a model and in investigating its behavior. Three-dimensional disturbances can be modeled in the vertically stratified ionosphere by forming wavepackets. The range of transverse scales covered by the model should be extended both downward and upward, so that such horizontally localized wave packets can be constructed. This would allow the "mapping" of electric-field (and the other field-quantities in the equation-set) over altitude to be modeled for more realistic disturbances, for possible experimental validation. The heating of the ionosphere should be calculated and compared to Poynting flux, to see if other forms of energy are playing a role. The nature of the altitude transition in wave properties should be studied, as there appears to be a degeneracy or near degeneracy having a profound effect. Additional boundary conditions should be developed so that the effects of the Thermal and Ion waves can be studied. And finally, the end goal is to make a model that is sufficiently robust that it can be deployed for public use, such as through the Community Coordinated Modeling Center (CCMC), the Integrated Geoscience Observatory (InGeo) [*Bhatt et al.*, 2020], or other systems for sharing methodology.

It is difficult to know where the model stands with respect to observational studies, since it is really necessary to evaluate each observational campaign in light of the model expectations for the particular event or statistical sampling of events. The problem is not easy. It is difficult to arrange for simultaneous and colocated (along $\vec{B}_0$) measurements of electric field in the E and F regions. And even though this is accomplished, it is difficult to sufficiently define the state of the background ionosphere, and the shape of the incoming signal. Resonant systems are notoriously sensitive and hard to model quantitatively. Like musical instruments, interferometers and microwave filters always require an accommodation for tuning. Thus, since the TL theory is predicting wavelike effects for the ionosphere, observational validation will require close attention to the sensitivity in characterization, along with the full cadre of observational capabilities.

TL theory is very useful for designing electrical circuits because it allows for understanding how they work. In bringing this theory to the ionosphere, it is important to distinguish between the detailed results that are presented herein as examples, and the physical conclusions that are far more robust. The finding that the electrical thickness of the ionosphere is significant (panel l of Figure 2) involves very little theoretical or computational uncertainty; it does not rely on the full complexity of the transmission line model; it is just a finding about the parallel wavelengths of the eigenmodes and how they depend on collision frequency. The finding for electrical thickness is an indicator for wavelike effects tied closely to the electromagnetic 5-moment fluid equations themselves, with an accordant level of physical relevance. The electrical thickness is the amount of phase rotation for a signal traversing the ionosphere, and we find that it often exceeds 90°, meaning a complete failure of electric field mapping, even for transverse wavelengths as long as 100 km. And the findings that the wave-admittances are not compatible with electrostatic theory (Section 3), and that there are two interacting modes to consider (Section 6), and that these modes become (nearly?) degenerate (Section 7), are similarly robust.

9 Appendix A: Matrix Form of the Electromagnetic Fluid Equations

We assume that electrodynamics in the ionosphere can be described by the 5-moment fluid equations [e.g., *Schunk and Nagy*, 2009 (eqns. 5.22 in the Second Edition)] for electrons and one species of ion, plus the dynamical Maxwell's equations, shown together in (A.1), where the subscripts denote either ions (i), electrons (e), or neutrals (n), m_α are the masses, $\vec{v}_\alpha$ are velocities, p_α are the pressures, $\nu_{\alpha\beta}$ is the collision frequency between species α and species β, T_n is the temperature of the neutral atmosphere (assumed independent), e is the absolute value of charge for an electron, ϵ_0 is the permittivity of free space, μ_0 is the permeability of free space, ξ is the recombination coefficient, Q is the background ionization rate, $\vec{E}$ is the electric field, and $\delta\vec{B}$ is the perturbation magnetic field (i.e., $\vec{B} = \vec{B}_0 + \delta\vec{B}$, where $\vec{B}_0$ is a background magnetic field, and it is assumed that $\nabla \times \vec{B}_0 = 0$):

$$\begin{aligned}
\frac{\partial n_e}{\partial t} &= -\vec{\nabla}\cdot(n_e\vec{v}_e) + Q - \xi\left(\frac{n_e+n_i}{2}\right)^2 \\
\frac{\partial n_i}{\partial t} &= -\vec{\nabla}\cdot(n_i\vec{v}_i) + Q - \xi\left(\frac{n_e+n_i}{2}\right)^2 \\
\frac{\partial \vec{v}_e}{\partial t} &= -\frac{e}{m_e}\left(\vec{E}+\vec{v}_e\times\vec{B}\right) - \nu_{en}\left(\vec{v}_e-\vec{v}_n\right) - \nu_{ei}\left(\vec{v}_e-\vec{v}_i\right) - \left(\vec{v}_e\cdot\vec{\nabla}\right)\vec{v}_e - \frac{\vec{\nabla}p_e}{m_e n_e} \\
\frac{\partial \vec{v}_i}{\partial t} &= \frac{e}{m_i}\left(\vec{E}+\vec{v}_i\times\vec{B}\right) - \nu_{in}\left(\vec{v}_i-\vec{v}_n\right) - \nu_{ie}\left(\vec{v}_i-\vec{v}_e\right) - \left(\vec{v}_i\cdot\vec{\nabla}\right)\vec{v}_i - \frac{\vec{\nabla}p_i}{m_i n_i} \\
\frac{\partial p_e}{\partial t} &= -\left(\vec{v}_e\cdot\vec{\nabla}\right)p_e - \frac{5}{3}p_e\left(\vec{\nabla}\cdot\vec{v}_e\right) - 2\frac{m_e\nu_{en}}{m_e+m_n}\left(p_e - n_e k_B T_n\right) - 2\frac{m_e\nu_{ei}}{m_e+m_i}\left(p_e - \frac{n_e}{n_i}p_i\right) \\
&+ \frac{2}{3}\frac{n_e m_e m_n \nu_{en}}{m_e+m_n}\left|\vec{v}_e-\vec{v}_n\right|^2 + \frac{2}{3}\frac{n_e m_e m_i \nu_{ei}}{m_e+m_i}\left|\vec{v}_e-\vec{v}_i\right|^2 \\
\frac{\partial p_i}{\partial t} &= -\left(\vec{v}_i\cdot\vec{\nabla}\right)p_i - \frac{5}{3}p_i\left(\vec{\nabla}\cdot\vec{v}_i\right) - 2\frac{m_i\nu_{in}}{m_i+m_n}\left(p_i - n_i k_B T_n\right) - 2\frac{m_i\nu_{ie}}{m_i+m_e}\left(p_i - \frac{n_i}{n_e}p_e\right) \\
&+ \frac{2}{3}\frac{n_i m_i m_n \nu_{in}}{m_i+m_n}\left|\vec{v}_i-\vec{v}_n\right|^2 + \frac{2}{3}\frac{n_i m_i m_e \nu_{ie}}{m_i+m_e}\left|\vec{v}_i-\vec{v}_e\right|^2 \\
\epsilon_0\frac{\partial \vec{E}}{\partial t} &= -e\left(n_i\vec{v}_i - n_e\vec{v}_e\right) + \mu_0^{-1}\vec{\nabla}\times\delta\vec{B} \\
\frac{\partial \delta\vec{B}}{\partial t} &= -\vec{\nabla}\times\vec{E}.
\end{aligned} \tag{A.1}$$

Sources such as gravity and photoelectrons are omitted from equation (A.1), and the neutral wind velocity ($\vec{v}_n$) will be set to zero.

We follow the approach in *Cosgrove* [2016] to analyze the equation-set (A.1). The equations are Fourier transformed in space and linearized. Linearization is justified by the idea that we are exploring the dynamical evolution of small perturbations (i.e., second order terms negligible) about some background state. The background state should be an exact solution to the full, non-linear equation-set (A.1), so that the equations will be satisfied in zeroth order, with only the higher order equations remaining to be solved. Therefore, we will use the only known exact solution to equation set (A.1), which is thermal equilibrium, that is, the zero-velocity homogeneous plasma with $n_e = n_i = \bar{n}_0$, temperatures equal to the neutral temperature (T_n), pressures equal to $k_B T_n \bar{n}_0$, $\vec{E} = 0$, $\delta\vec{B} = 0$, and Q set to balance the recombination term, so that all the time derivatives are zero.

Thermal equilibrium is technically a special case of electrostatic equilibrium, in the sense that all the time derivatives are zero. However, the non-trivial solutions of electrostatic theory are derived by linearizing the equation-set (A.1) [*Farley*, 1959] (more commonly a reduced set of equations is used), and so a background state that satisfies the equations is also needed to derive electrostatic equilibrium (otherwise, the nonlinear terms cannot be made small). Here, again, thermal equilibrium is the only rigorous choice, being the only known exact solution. So both electrostatic equilibrium and the electromagnetic solution we develop here

are, canonically, solutions that describe small perturbations about thermal equilibrium. (The generalization to small perturbations about an inhomogeneous background state is discussed in Section 5, and is heuristic in nature.) Hence, it is appropriate to compare our electromagnetic solution with electrostatic equilibrium, and the former can serve as a test for the latter.

In thermal equilibrium the electron and ion velocities and the electric field are all zero. Hence, we write the dynamical variables as the sum of zeroth and first order parts as follows,

$$\begin{aligned} n_e &= \bar{n}_0 + \delta n_e, \\ n_i &= \bar{n}_0 + \delta n_i, \\ \vec{v}_e &= \delta \vec{v}_e, \\ \vec{v}_i &= \delta \vec{v}_i, \\ p_e &= k_B T_n \bar{n}_0 + \delta p_e, \\ p_i &= k_B T_n \bar{n}_0 + \delta p_i, \\ \vec{E} &= \delta \vec{E}, \\ \vec{B} &= \vec{B}_0 + \delta \vec{B}, \end{aligned} \tag{A.2}$$

where T_n is the neutral temperature, k_B is Boltzmann's constant, $\vec{B}_0$ is the geomagnetic field, and $\bar{n}_0$ is the background plasma density.

Substituting the forms (A.2) into the equations of motion (A.1) and taking the Fourier transform in space gives, after arranging into matrix notation, the general equation (7) from Section (4), where

$$\begin{aligned} \vec{X} &= (V_{ex}, V_{ey}, V_{ez}, V_{ix}, V_{iy}, V_{iz}, E_x, E_y, E_z, c\delta B_x, c\delta B_y, c\delta B_z, \delta N_e, \delta N_i, \delta P_e, \delta P_i), \\ V_{\alpha\beta} &= \frac{m_\alpha}{e} \omega_\alpha v_{\alpha\beta}, \\ \delta N_\alpha &= \frac{m_e}{m_i} \frac{e}{\epsilon_0 \left|\vec{k}\right|} \delta n_\alpha, \\ \delta P_\alpha &= \frac{m_e}{m_i} \frac{e}{\epsilon_0 \left|\vec{k}\right| k_B T_n} \delta p_\alpha, \end{aligned}$$

$\omega_\alpha = \sqrt{\frac{\bar{n}_0 e^2}{m_\alpha \epsilon_0}}$ are the electron and ion plasma frequencies, $\vec{k}$ is the wavevector for the Fourier transform, and the matrix H_5 is shown in detail in Figure A.1. In this figure, $\Omega_{i0} = eB_0/m_i$ is the ion gyro-frequency, $\Omega_{e0} = eB_0/m_e$ is the electron gyro-frequency, $\hat{B} = \vec{B}_0/B_0$, $\vec{V}_{e0}$ and $\vec{V}_{i0}$ are the (not rescaled) background electron and ion velocities (which we set to zero, for thermal equilibrium), and P_{e0} and P_{i0} are the background electron and ion pressures (which we set to $k_B T_n \bar{n}_0$, for thermal equilibrium). The rescalings provide that the dynamical variables all have units of electric field, and that the matrix elements of H_5 all have units of frequency. To be clear, H_5 is formed without dropping any terms or making any approximations.

As can be seen from the homogeneous solution (9) from Section(4), the eigenvalues of H_5 are the complex frequencies ω_j of the supported waves, where the real part is the frequency of oscillation, and the imaginary part is the dissipation rate. The eigenvectors of H_5 are the polarizations of the supported waves, that is, they determine the relative amplitudes and phases for the dynamical variables in $\vec{X}$.

This formulation is equivalent to deriving the complete linear dispersion relation for the equation set (A.1), and solving it for complex ω_j as a function of real $\vec{k}$. However, although finding the exact analytical dispersion relation for (A.1) is possible with due patience, finding the frequencies that satisfy the dispersion relation still requires finding the 16 complex roots of a polynomial, and does not determine the polarization vectors. In our matrix based approach all this work is done by standard matrix analysis tools. We solve for the eigenvectors/eigenvalues numerically using numpy.linalg.eig from the NumPy Python library.

In order to evaluate electrostatic waves, we will compare results using the full equations (A.1) with results from the same equations, except with the curl of the electric field assumed to be zero. To find the reduced equations substitute $\vec{E} = -\vec{\nabla}\phi$ into the equations (A.1). With this substitution Faraday's law provides that the magnetic perturbation $\left(\delta\vec{B}\right)$ is zero, and so the three equations of Faraday's law decouple from

$$
H_5 = i \left[\begin{array}{cccccc}
\nu_{en} + \nu_{ei} - i\vec{k}\cdot\vec{V}_{e0} + \gamma_x V_{e0x} & \Omega_{e0}\hat{B}_z + \gamma_y V_{e0x} & -\Omega_{e0}\hat{B}_y + \gamma_z V_{e0x} & -\nu_{ei}\sqrt{\frac{m_e}{m_i}} & 0 & 0 \\
-\Omega_{e0}\hat{B}_z + \gamma_x V_{e0y} & \nu_{en} + \nu_{ei} - i\vec{k}\cdot\vec{V}_{e0} + \gamma_y V_{e0y} & \Omega_{e0}\hat{B}_x + \gamma_z V_{e0y} & 0 & -\nu_{ei}\sqrt{\frac{m_e}{m_i}} & 0 \\
\Omega_{e0}\hat{B}_y + \gamma_x V_{e0z} & -\Omega_{e0}\hat{B}_x + \gamma_y V_{e0z} & \nu_{en} + \nu_{ei} - i\vec{k}\cdot\vec{V}_{e0} + \gamma_z V_{e0z} & 0 & 0 & -\nu_{ei}\sqrt{\frac{m_e}{m_i}} \\
-\nu_{ie}\sqrt{\frac{m_i}{m_e}} & 0 & 0 & \nu_{in} + \nu_{ie} - i\vec{k}\cdot\vec{V}_{i0} + \gamma_x V_{i0x} & -\Omega_{i0}\hat{B}_z + \gamma_y V_{i0x} & \Omega_{i0}\hat{B}_y + \gamma_z V_{i0x} \\
0 & -\nu_{ie}\sqrt{\frac{m_i}{m_e}} & 0 & \Omega_{i0}\hat{B}_z + \gamma_x V_{i0y} & \nu_{in} + \nu_{ie} - i\vec{k}\cdot\vec{V}_{i0} + \gamma_y V_{i0y} & -\Omega_{i0}\hat{B}_x + \gamma_z V_{i0y} \\
0 & 0 & -\nu_{ie}\sqrt{\frac{m_i}{m_e}} & -\Omega_{i0}\hat{B}_y + \gamma_x V_{i0z} & \Omega_{i0}\hat{B}_x + \gamma_y V_{i0z} & \nu_{in} + \nu_{ie} - i\vec{k}\cdot\vec{V}_{i0} + \gamma_z V_{i0z} \\
-\omega_e & 0 & 0 & \omega_i & 0 & 0 \\
0 & -\omega_e & 0 & 0 & \omega_i & 0 \\
0 & 0 & -\omega_e & 0 & 0 & \omega_i \\
0 & 0 & 0 & 0 & 0 & 0 \\
0 & 0 & 0 & 0 & 0 & 0 \\
0 & 0 & 0 & 0 & 0 & 0 \\
\frac{m_e}{m_i}\frac{\omega_e}{|\vec{k}|}(\gamma_x - ik_x) & \frac{m_e}{m_i}\frac{\omega_e}{|\vec{k}|}(\gamma_y - ik_y) & \frac{m_e}{m_i}\frac{\omega_e}{|\vec{k}|}(\gamma_z - ik_z) & 0 & 0 & 0 \\
0 & 0 & 0 & \frac{m_e}{m_i}\frac{\omega_i}{|\vec{k}|}(\gamma_x - ik_x) & \frac{m_e}{m_i}\frac{\omega_i}{|\vec{k}|}(\gamma_y - ik_y) & \frac{m_e}{m_i}\frac{\omega_i}{|\vec{k}|}(\gamma_z - ik_z) \\
\frac{m_e}{m_i}A_{eix} & \frac{m_e}{m_i}A_{eiy} & \frac{m_e}{m_i}A_{eiz} & \frac{m_e}{m_i}C_{eix} & \frac{m_e}{m_i}C_{eiy} & \frac{m_e}{m_i}C_{eiz} \\
\frac{m_e}{m_i}C_{iex} & \frac{m_e}{m_i}C_{iey} & \frac{m_e}{m_i}C_{iez} & \frac{m_e}{m_i}A_{iex} & \frac{m_e}{m_i}A_{iey} & \frac{m_e}{m_i}A_{iez}
\end{array} \right.
$$

$$
\left. \begin{array}{cccccccccc}
\omega_e & 0 & 0 & 0 & \frac{-V_{e0z}}{c}\omega_e & \frac{V_{e0y}}{c}\omega_e & 0 & 0 & \frac{m_i}{m_e}\frac{k_BT_n|\vec{k}|}{m_e\omega_e}(-ik_x - \gamma_x) & 0 \\
0 & \omega_e & 0 & \frac{V_{e0z}}{c}\omega_e & 0 & \frac{-V_{e0x}}{c}\omega_e & 0 & 0 & \frac{m_i}{m_e}\frac{k_BT_n|\vec{k}|}{m_e\omega_e}(-ik_y - \gamma_y) & 0 \\
0 & 0 & \omega_e & \frac{-V_{e0y}}{c}\omega_e & \frac{V_{e0x}}{c}\omega_e & 0 & 0 & 0 & \frac{m_i}{m_e}\frac{k_BT_n|\vec{k}|}{m_e\omega_e}(-ik_z - \gamma_z) & 0 \\
-\omega_i & 0 & 0 & 0 & \frac{V_{i0z}}{c}\omega_i & \frac{-V_{i0y}}{c}\omega_i & 0 & 0 & 0 & \frac{k_BT_n|\vec{k}|}{m_e\omega_i}(-ik_x - \gamma_x) \\
0 & -\omega_i & 0 & \frac{-V_{i0z}}{c}\omega_i & 0 & \frac{V_{i0x}}{c}\omega_i & 0 & 0 & 0 & \frac{k_BT_n|\vec{k}|}{m_e\omega_i}(-ik_y - \gamma_y) \\
0 & 0 & -\omega_i & \frac{V_{i0y}}{c}\omega_i & \frac{-V_{i0x}}{c}\omega_i & 0 & 0 & 0 & 0 & \frac{k_BT_n|\vec{k}|}{m_e\omega_i}(-ik_z - \gamma_z) \\
0 & 0 & 0 & 0 & -cik_z & cik_y & -\frac{m_i}{m_e}|\vec{k}|V_{e0x} & \frac{m_i}{m_e}|\vec{k}|V_{i0x} & 0 & 0 \\
0 & 0 & 0 & cik_z & 0 & -cik_x & -\frac{m_i}{m_e}|\vec{k}|V_{e0y} & \frac{m_i}{m_e}|\vec{k}|V_{i0y} & 0 & 0 \\
0 & 0 & 0 & -cik_y & cik_x & 0 & -\frac{m_i}{m_e}|\vec{k}|V_{e0z} & \frac{m_i}{m_e}|\vec{k}|V_{i0z} & 0 & 0 \\
0 & cik_z & -cik_y & 0 & 0 & 0 & 0 & 0 & 0 & 0 \\
-cik_z & 0 & cik_x & 0 & 0 & 0 & 0 & 0 & 0 & 0 \\
cik_y & -cik_x & 0 & 0 & 0 & 0 & 0 & 0 & 0 & 0 \\
0 & 0 & 0 & 0 & 0 & 0 & \vec{\gamma}\cdot\vec{V}_{e0} - i\vec{k}\cdot\vec{V}_{e0} + \xi\bar{n}_0 & \xi\bar{n}_0 & 0 & 0 \\
0 & 0 & 0 & 0 & 0 & 0 & \xi\bar{n}_0 & \vec{\gamma}\cdot\vec{V}_{i0} - i\vec{k}\cdot\vec{V}_{i0} + \xi\bar{n}_0 & 0 & 0 \\
0 & 0 & 0 & 0 & 0 & 0 & D_{ei} & \frac{2m_e\nu_{ei}P_{io}}{(m_e+m_i)k_BT_n\bar{n}_0} & L_{ei} & -2\frac{m_e\nu_{ei}}{m_e+m_i} \\
0 & 0 & 0 & 0 & 0 & 0 & \frac{2m_i\nu_{ie}P_{eo}}{(m_e+m_i)k_BT_n\bar{n}_0} & D_{ie} & -2\frac{m_i\nu_{ie}}{m_e+m_i} & L_{ie}
\end{array} \right], \text{ where}
$$

$$
A_{\alpha\beta} = \frac{\vec{\gamma}}{|\vec{k}|}\frac{P_{\alpha0}\omega_\alpha}{k_BT_n\bar{n}_0} - \frac{5}{3}\frac{i\vec{k}}{|\vec{k}|}\frac{P_{\alpha0}\omega_\alpha}{k_BT_n\bar{n}_0} - \frac{4}{3}\frac{(\vec{V}_{\alpha0} - \vec{V}_n)\frac{\omega_\alpha}{|\vec{k}|}}{k_BT_n/m_\alpha}\frac{m_n\nu_{\alpha n}}{m_\alpha + m_n} - \frac{4}{3}\frac{(\vec{V}_{\alpha0} - \vec{V}_{\beta0})\frac{\omega_\alpha}{|\vec{k}|}}{k_BT_n/m_\alpha}\frac{m_\beta\nu_{\alpha\beta}}{m_\alpha + m_\beta},
$$

$$
C_{\alpha\beta} = \frac{4}{3}\frac{(\vec{V}_{\alpha0} - \vec{V}_{\beta0})\frac{\omega_\beta}{|\vec{k}|}}{k_BT_n/m_\alpha}\frac{m_\beta\nu_{\alpha\beta}}{m_\alpha + m_\beta},
$$

$$
D_{\alpha\beta} = -2\frac{m_\alpha\nu_{\alpha n}}{m_\alpha + m_n} - 2\frac{m_\alpha\nu_{\alpha\beta}P_{\beta0}}{(m_\alpha + m_\beta)k_BT_n\bar{n}_0} - \frac{2}{3}\frac{m_n\nu_{\alpha n}}{m_\alpha + m_n}\frac{\left|\vec{V}_{\alpha0} - \vec{V}_n\right|^2}{k_BT_n/m_\alpha} - \frac{2}{3}\frac{m_\beta\nu_{\alpha\beta}}{m_\alpha + m_\beta}\frac{\left|\vec{V}_{\alpha0} - \vec{V}_{\beta0}\right|^2}{k_BT_n/m_\alpha},
$$

$$
L_{\alpha\beta} = -i\vec{k}\cdot\vec{V}_{\alpha0} + \frac{5}{3}\left(\vec{\gamma}\cdot\vec{V}_{\alpha0}\right) + 2\frac{m_\alpha\nu_{\alpha n}}{m_\alpha + m_n} + 2\frac{m_\alpha\nu_{\alpha\beta}}{m_\alpha + m_\beta}.
$$

Figure A.1: Matrix H_5 containing the linearized and Fourier transformed electromagnetic five-moment fluid equations. In this work $\vec{V}_{e0}$ and $\vec{V}_{i0}$ are set to zero, and P_{e0} and P_{i0} are set to $k_BT_n\bar{n}_0$.

the other equations. However, Ampere's law becomes an over-determined set of three equations for the one variable (ϕ). To render these equations consistent, recall that a vector field can be decomposed into a curl-free part and a divergence-free part, where the curl-free part is uniquely determined by the divergence of the vector field. Therefore, with the assumption $\vec{\nabla} \times \vec{E} = 0$, it is sufficient to replace Ampere's law with an equation for $\vec{\nabla} \cdot \vec{E}$, which we can get by taking the divergence of Ampere's law. In fact, taking the divergence of Ampere's law with $\vec{E} = -\vec{\nabla}\phi$ gives the time derivative of the Poisson equation, which is equivalent to the Poisson equation, since the latter has no inhomogeneous term. So in summary, the electrostatic version of the 5-moment equations is obtained by dropping Faraday's law and replacing Ampere's law with its divergence, which is equivalent to using the Poisson equation.

It should also be noted that taking the curl of Ampere's law under the assumption $\vec{E} = -\vec{\nabla}\phi$ results in the equation $\vec{\nabla} \times \vec{J} = \vec{\nabla} \times \vec{\nabla} \times \delta\vec{B}/\mu_0$, where $\vec{J}$ is the current density. Generally, because the curl of the current is not zero, this equation cannot be satisfied when $\delta\vec{B} = 0$, and so the electrostatic waves are not solutions for the electromagnetic equations. It is generally not consistent to assume that $\vec{\nabla} \times \vec{E}$ is exactly zero. Rather, in using electrostatic waves, there is an assumption that $\vec{\nabla} \times \vec{E}$ is sufficiently small such that the electrostatic waves are good approximations for the actual electromagnetic waves.

The matrix H_{5ES} for the electrostatic version of equation (7) is given in Figure S.1 of the Supplementary Information, where

$$\vec{X}_{ES} = \left(V_{ex}, V_{ey}, V_{ez}, V_{ix}, V_{iy}, V_{iz}, \left|\vec{k}\right| \phi, \delta N_e, \delta N_i, \delta P_e, \delta P_i\right).$$

The electrostatic waves are obtained as the eigenvalues/eigenvectors of H_{5ES}.

10 Appendix B: Integral Approximations

This appendix describes an additional approximation for the integrals in the first sum of equation (12), for the case of modes that have a minimum operating frequency, such as the Whistler wave, which becomes cutoff when the operating frequency drops too low. This result goes along with the result (14) that applies for modes such as the Alfvén wave, which can propagate all the way down to DC. We also make some comments on the integrals for modes below the operating frequency.

The integral approximation (13) for the propagating modes was obtained by linearizing about $k_z = k_{0z}$ such that $\mathrm{real}\left(\omega_\mathrm{j}(0, \mathrm{k_{0y}}, \mathrm{k_{0z}})\right) = \omega_0$. This is only possible for propagating modes, that is, for modes that can achieve the frequency ω_0 for some k_z. An important class of modes that we need to include in our model are those which may not always be able to go low enough in frequency. For example, when the Whistler wave is cutoff, above the E region, it is because the frequency cannot go low enough. In this case the greatest contribution to the integral comes at closest approach to ω_0, which occurs at $k_z = 0$, and the saturation of frequency as $k_z \to 0$ can be modeled by a Taylor series expansion about $k_z = 0$,

$$\omega_j\left(k_z\right) \cong \frac{1}{2} \left.\frac{\partial^2 \omega_j}{\partial k_z^2}\right|_{k_z=0} k_z^2 + \omega_{jrmin} + i\omega_{ji}|_{k_z=0}\,, \tag{B.1}$$

where the linear term is omitted because there must be symmetry about $k_z = 0$, and we drop terms higher than second order. Numerical examination of the dependence of ω_j on k_z for the Whistler wave confirms that this model is reasonable in the region around $k_z = 0$. In fact, this being the case, we note that for large k_z the curvature becomes negligible compared to the slope, and we would expect this quadratic model to recover the solution (14) from the linear model in the event that there exists some large $k_z = k_{0z}$ where $\mathrm{real}\left(\omega_j(k_{0z})\right) = \omega_0$, if the slope at $k_z = k_{0z}$ is matched. Therefore, for the higher frequency modes that saturate their lower bound at $k_z = 0$, we can use the quadratic model for both propagating and non-propagating cases, and thereby recover a continuous result across the propagating/non-propagating divide. To do this we devise the rule of fitting the quadratic form $ak_z^2 + \zeta$ at the point of closest approach to ω_0, and if this occurs away from $k_z = 0$, meaning that equality was achieved and the mode propagates, we fit the slope at k_{0z}:

$$\omega_j\left(k_z\right) = \frac{1}{2k_{0z}} \left.\frac{\partial \omega_j}{\partial k_z}\right|_{k_z=k_{0z}} k_z^2 + \omega_0 + i\omega_{ji}|_{k_z=k_{0z}} - \frac{k_{0z}}{2} \left.\frac{\partial \omega_j}{\partial k_z}\right|_{k_z=k_{0z}}. \tag{B.2}$$

This form approaches 0/0 as $k_{0z} \to 0$, and so when closest approach happens at $k_z = 0$ we replace it with the form (B.1). By the symmetry property of ω_j about $k_z = 0$, the quadratic approximation becomes exact as $k_z \to 0$ (higher order terms vanish), and so the forms (B.1) and (B.2) are consistent, and there will be continuity around $k_z = 0$.

Substituting either the form (B.1) or the form (B.2) into the integral form (12) and using $\vec{h}'_j$ and a'_j from (13) gives,

$$\int_{-\infty}^{\infty} \mathrm{d}k_z \frac{\vec{h}'_j(k_z - k_{0z})a'_j(k_z - k_{0z})}{ak_z^2 + c} \mathrm{e}^{ik_z z}$$
$$= \int_{-\infty}^{\infty} \mathrm{d}k_z \frac{\vec{h}'_j(k_z - k_{0z})a'_j(k_z - k_{0z})}{\left(k_z - \sqrt{-ac}/a\right)\left(k_z + \sqrt{-ac}/a\right)a} \mathrm{e}^{ik_z z},$$

where either $a = \frac{-i}{2} \left.\frac{\partial^2 \omega_j}{\partial k_z^2}\right|_{k_z=0}$ and $c = i\left(\omega_0 - \omega_{jrmin}\right) + \left.\omega_{ji}\right|_{k_z=0}$, or $a = \frac{-i}{2k_{0z}} \left.\frac{\partial \omega_j}{\partial k_z}\right|_{k_z=k_{0z}}$ and $c = \left.\omega_{ji}\right|_{k_z=k_{0z}} + \frac{ik_{0z}}{2} \left.\frac{\partial \omega_j}{\partial k_z}\right|_{k_z=k_{0z}}$. There are two simple poles, and applying partial fraction expansion allows application of the residue theorem. Omitting these standard integration steps we obtain for the integral,

$$\vec{h}'_j\left(\pm\sqrt{-ac}/a - k_{0z}\right) a'_j\left(\pm\sqrt{-ac}/a - k_{0z}\right) \frac{i}{2\sqrt{-ac}} \mathrm{e}^{\pm i(\sqrt{-ac}/a)z}, \tag{B.3}$$

where the $\pm$ option is chosen to give exponential decay away from the source, depending on the sign of z, and the negative option for k_{0z} is used with the - sign. Testing finds that this relation does indeed provide for continuity of the wavelength, and rapid reduction of the Whistler mode dissipation scale length to about ten kilometers, across the propagating/non-propagating altitude boundary. Also, it can be shown that the solution (B.3) reduces to the solution (14) when k_{0z} is large.

The quadratic solution (B.3) is appropriate for the Whistler wave, and probably also for the higher frequency waves. For the Alfvén wave we should continue to use the driven steady-state solution (14), because the quadratic solution (B.3) is not appropriate. The Alfvén wave has a different form of dispersion relation, that crosses through zero-frequency with a non-zero k_z derivative. For example, the ideal Alfvén wave dispersion relation is simply $\omega = \pm v_A k_z$, which is most naturally viewed as having two antisymmetric branches, but which for purposes of comparing with the quadratic dispersion relations (B.1) and (B.2), which are symmetric, can be viewed as having upward and downward V-shaped branches, which are symmetric and linear except for having a singularity at $k_z = 0$. So for the Alfvén wave the linearized approximation of equation (14) is highly appropriate.

The validations given in Section 4 also apply to the quadratic form (B.3), as can be seen by writing out the expression for the residue location in the case (B.2). The residue location comes out to be $k_{0z}\sqrt{1 + 2i\omega_{ji}/(k_{0z}v_{gj})}$. Expanding the square-root shows that the first two terms are exactly the same as those found for the linear approximation of Section 4, and so the same validations apply.

It remains to develop approximations for the dispersion relations of the Ion wave, Thermal wave, and of the lowest-frequency cutoff mode. However, there is every indication that polynomial approximations can be found as in the above. As long as the denominator is a polynomial in k_z, we can solve the integral using the residue theorem, even if roots must be found numerically. Whether these must be higher order is not yet known. The biggest obstacle to a higher order approximation is estimation of the higher orders of derivative of ω_j.

Open Research

This is a theoretical paper that does not rely on data. No special software has been used in this research.

Acknowledgements

This material is based upon work supported by the National Science Foundation (NSF) under NSF grant AGS-1344300.

References

Aikio, A. T., T. Lakkala, A. Kozlovsky, and P. J. S. Williams (2002), Electric fields and currents of stable drifting auroral arcs in the evening sector, *J. Geophys. Res.*, *107, A12*, 1424.

Akbari, H., R. Pfaff, J. Clemmons, H. Freudenreich, D. Rowland, and A. Streltsov (2022), Resonant alfvén waves in the lower auroral ionosphere: Evidence for the nonlinear evolution of the ionospheric feedback instability, *J. Geophys. Res., Space*, *127*(2).

Artin, M. (1991), *Algebra*, Prentice Hall, Englewood Cliffs, New Jersey 07632.

Aviero, H., and D. Hysell (2012), Implications of the equipotential field line approximation for equatorial spread f anaylsis, *Geophys. Res. Lett.*, *39*(L11106).

Bernstein, I. B. (1958), Waves in a plasma in a magnetic field, *Phys. Rev.*, *109*(1), 10–21.

Bhatt, A., T. Valentic, A. Reimer, L. Lamarche, P. Reyes, and R. Cosgrove (2020), Reproducible software environment: a tool enabling computational reproducibility in geospace sciences and facilitating collaboration, *J. Space Weather Space Clim.*, *10*(12).

Bhattacharyya, A., and W. J. Burke (2000), A transmission line analogy for the development of equatorial ionospheric bubbles, *J. Geophys. Res.*, *105*(A11), 24,941–24,950.

Birk, G. T., and A. Otto (1996), A three-dimensional plasma-neutral gas-fluid code, j. comput. phys., *J. Comput. Phys.*, *125*, 513–525.

Collin, R. E. (1966), *Foundations for Microwave Engineering*, McGraw-Hill.

Cosgrove, R. B. (2016), Does a localized plasma disturbance in the ionosphere evolve to electrostatic equilibrium? evidence to the contrary, *J. Geophys. Res.*, *121*, doi:https://doi.org/10.1002/2015JA021672.

Cosgrove, R. B., and R. Doe (2010), Effect of ionospheric depth on the ionospheric feedback instability: cutoff and subsequent $E_{\|}$ modes, *Ann. Geophys.*, *28*, pp. 1777–1794, doi:https://doi.org/10.5194/angeo-28-1777-2010.

Cosgrove, R. B., and R. T. Tsunoda (2002), A direction-dependent instability of sporadic E layers in the nighttime midlatitude ionosphere, *Geophys. Res. Lett.*, *29(18)*, 1864.

Cosgrove, R. B., and R. T. Tsunoda (2004), Instability of the E-F coupled nighttime midlatitude ionosphere, *J. Geophys. Res.*, *109*, a04305.

Cosgrove, R. B., H. Bahcivan, S. Chen, R. J. Strangeway, J. Ortega, M. Alhassan, Y. Xu, M. V. Welie, J. Rehberger, S. Musielak, and N. Cahill (2014), Empirical model of poynting flux derived from fast data and a cusp signature, *J. Geophys. Res.*, *119*, 411–430, doi:https://doi.org/10.1002/2013JA019105.

Dao, E., C. E. Seyler, and M. C. Kelley (2013), Three dimensional modeling of the electromagnetic characteristics of equatorial plasma depletions, *J. Geophys. Res.*, *118*(6), 3505–3514, doi:https://doi.org/10.1002/jgra.50216.

Eccles, J. V., J. P. S. Maurice, and R. W. Schunk (2015), Mechanisms underlying the prereversal enhancement of the vertical plasma drift in the low-latitude ionosphere, *J. Geophys. Res., Space*, *120*(6), 4950–4970.

EEsoft, C. (), *Touchstone is the classical microwave/RF analysis program developed by EEsoft company in late 80-ies. The company was bought by HP (now Agilent). They established many things that are inherited by the microwave community.*

Farley, D. T. (1959), A theory of electrostatic fields in a horizontally stratified ionosphere subject to a vertical magnetic field, *J. Geophys. Res.*, *64*, 1225.

Farley, D. T. (1960), A theory of electrostatic fields in the ionosphere at nonpolar geomagnetic latitudes, *J. Geophys. Res.*, *65*, 869.

Farley, D. T., E. Bonelli, B. G. Fejer, and M. F. Larsen (1986), The prereversal enhancement of the zonal electric field in the equatorial ionosphere, *J. Geophys. Res., Space*, *91*(A12), 13,723–13,728.

Fuller-Rowell, T. J., and D. S. Evans (1987), Height-integrated Pedersen and Hall conductivity patterns inferred from the TIROS-NOAA satellite data, *J. Geophys. Res.*

Garcia, F. J., M. C. Kelley, and J. J. Makela (2000), Airglow observations of mesoscale low-velocity traveling ionospheric disturbances at midlatitudes, *J. Geophys. Res.*, *105*, 18,407–18,415.

Goertz, C. K., and R. W. Boswell (1979), Magnetosphere-ionosphere coupling, *J. Geophys. Res.*, *84(A12)*, pp. 7239–7246.

Gombosi, T. I., D. L. D. Zeeuw, C. P. Groth, K. G. Powell, C. R. Clauer, and P. Song (2001), From sun to earth: Multiscale mhd simulations ofspace weather, *Space Weather, Geophys. Monogr. Ser., edited by P. Song, H. J. Singer, and G. L. Siscoe*, *125*, 169–176.

Haldoupis, C., K. Schlegel, and D. T. Farley (1996), An explanation for type 1 radar echoes from the midlatitude *E*-region ionosphere, *Geophys. Res. Lett.*, *23*, 97.

Heinselman, C. J., and M. J. Nicolls (2008), A bayesian approach to electric field ande-region neutralwind estimation with the poker flat advanced modularincoherent scatter radar, *Radio Science*, *43*(RS5013).

Hughes, W. J. (1974), The effect of the atmosphere and ionosphere on llong period magnetospheric micropulsations, *Planet. Space Sci.*, *22*, pp. 1157–1172.

Hysell, D. L., M. Yamamoto, and S. Fukao (2002), Simulations of plasma clouds in the midlatitude e region ionosphere with implications for type i and type ii quasiperiodic echoes, *J. Geophys. Res.*, *107*(A10), 1313.

Hysell, D. L., M. F. Larsen, and Q. H. Zhou (2004), Common volume coherent and incoherent scatter radar observations of mid-latitude sporadic *E*-layers and QP echoes, *Ann. Geophys.*, pp. 3277–3290, sRef-ID: 1432-0576/ag/2004-22-3277.

Jackson, J. D. (1962, 1975), *Classical Electrodynamics*, second ed., John Wiley and Sons, Inc.

Janhunen, P., M. Palmroth, T. Laitinen, I. Honkonen, L. Juusola, G. Facskó, and T. Pulkkinen (2012), The gumics-4 global mhd magnetosphere-ionosphere coupling simulation, *Journal of Atmospheric and Solar-Terrestrial Physics*, *80*, 48–59.

Juusola, L., W. E. Archer, K. Kauristie, J. K. Burchill, H. Vanhamäki, and A. Aikio (2016), Ionospheric conductances and currents of a morning sector auroral arc from swarm-a electric and magnetic field measurements, *Geophys. Res. Lett.*, *43*(22), 11,519–11,527.

Kagan, L., T. Ogawa, S. Fukao, and M. Yamamoto (2000), A role of neutral motions in formation of midlatitude E-region field-aligned irregularities, *Geophys. Res. Lett.*, *27*(7), 939–942.

Kelley, M., and L. Gelinas (2000), Gradient drift instability in midlatitude sporadic e layers: localization of physical and wavenumber space, *Geophys. Res. Lett.*, *27*(4), 457–460.

Kelley, M. C. (2009), *Earth's Ionosphere, Plasma Physics and Electrodynamics (Second Edition)*, Academic Press, New York.

Knipp, D., S. Eriksson, L. Kilcommons, G. Crowley, J. Lei, M. Hairston, and K. Drake (2011), Extreme poynting flux in the dayside thermosphere: Examples and statistics, *Geophys. Res. Lett.*, *38*(L16102).

Knudsen, D., M. Kelley, and J. Vickrey (1992), Alfven waves in the auroral ionosphere: a numerical model compared with measurements, *J. Geophys. Res.*, *97(A1)*, pp. 77–90.

Kuzichev, I. V., I. Vasko, A. Malykhin, and A. R. Soto-Chavez (2018), On the ionospheric propagation of vlf waves generated by currents in the t lower ionosphere, *J. Atmos. Solar-Terr. Phys.*, *179*, 138–148.

Lotko, W., R. H. Smith, B. Zhang, J. E. Ouellette, O. J. Brambles, and J. G. Lyon (2014), Ionospheric control of magnetotail reconnection, *Science*, *345*(6193), 184–187.

Lyon, J., J. Fedder, and C. Mobarry (2004), The lyon-fedder-mobarry (lfm) global mhd magnetospheric simulation code, *J. Atmos. Solar-Terr. Phys.*, *66*, 1333.

Lysak, R. L. (1990), Electrodynamic coupling of the magnetosphere and ionosphere, *Space Sci. Rev.*, *52*(1-2), 33–38.

Lysak, R. L. (1991), Feedback instability of the ionospheric resonant cavity, *J. Geophys. Res.*, *96(A2)*, pp. 1553–1568.

Lysak, R. L., C. L. Waters, and M. D. Sciffer (2013), Modeling of the ionospheric Alfven resonator in dipolar geometry, *J. Geophys. Res.*, *118*, 1514–1528.

Malherbe, J. (1979), *Microwave Transmission Line Filters*, Artech House, Inc.

Mallinckrodt, A. J., and C. W. Carlson (1978), Relations between transverse electric fields and field-aligned currents, *J. Geophys. Res.*, *83(A4)*, pp. 1426–1432.

Maltsev, Y. P., W. B. Lyatsky, and A. M. Lyatskaya (1977), Currents over the auroral arc, *Planet. Space Sci.*, *25*, pp. 53–57.

Marklund, G. I., I. Sandahl, and H. Opgenoorth (1982), A study of the dynamics of a discrete auroral arc, *Planet. Space Sci.*, *30*(2), 179–197, doi:10.1016/0032-0633(82)90088-5.

Matthaei, G., L. Young, and E. M. T. Jones (1980), *Microwave Filters, Impedance Matching Networks, and Coupling Structures*, Arthech House, Inc.

Miano, G., and A. Maffucci (2001), *Transmission Lines and Lumped Circuits*, Academic Press.

Nicolls, M. J., R. Cosgrove, and H. Bahcivan (2014), Estimating the vector electric field using monostatic, multibeam incoherent scatter radar measurements, *Radio Science*, *49*, 1124–1139.

Nilsson, J. W. (1984), *Electric Circuits*, Addison Wesley.

Otto, A., and H. Zhu (2003), Fluid plasma simulation of coupled systems: Ionosphere and Magnetosphere, *LNP: Space Plasma Simulation*, *615*, 193.

Pakhotin, I. P., D. J. Knudsen, R. L. Lysak, and J. K. Burchill (2020), Diagnosing the role of Alfvén waves in global field-aligned current system dynamics during southward imf: swarm observations, *J. Geophys. Res., Space*, *125*.

Pakhotin, I. P., I. R. Mann, K. Xie, and D. J. Knudsen (2021), Northern preference for terrestrial electromagnetic energy input from space weather, *Nat Commun*, *12*(199).

Pokhotelov, D., V. Khruschev, M. Parrot, S. Senchenkov, and V. P. Pavlenko (2001), Ionospheric Alfven resonator revisited: Feedback instability, *J. Geophys. Res.*, *106(A11)*, pp. 25,813–25,824.

Raeder, J., J. Berchem, and M. Ashour-Abdalla (1998), The geospace environment grand challenge: Results from a global geospace circulation model, *J. Geophys. Res.*, *103*, 14,787.

Saleem, H. (2001), Can the inertial alfvén wave become an electrostatic mode in the limit $1 << \lambda_e^2 k^2$?, *J. Fusion Energy*, *20*, 57–59.

Sato, T. (1978), A theory of quiet auroral arcs, *J. Geophys. Res.*, *83(A3)*, pp. 1042–1048.

Schunk, R. W., and A. F. Nagy (2009), *Ionospheres: Physics, Plasma Physics, and Chemistry*, Atmospheric and Space Science Series, second edition ed., Cambridge University Press, University Printing House, Cambridge CB2 8BS, United Kingdom.

Seyler, C. (1990), A mathematical model of the structure and evolution of small-scale discrete auroral arcs, *J. Geophys. Res.*, *95(A10)*, pp. 17,199–17,215.

Song, Y., and R. L. Lysak (2006), Displacement current and generation of parallel electric fields, *Phys. Rev. E*, *96*, 145,002.

Spreiter, J. R., and B. R. Briggs (1961), Theory of electrostatic fields in the ionosphere at equatorial latitudes, *J. Geophys. Res.*, *66*, 2345.

Stix, T. H. (1992), *Waves in Plasmas*, American Institute of Physics, New York.

Streltsov, A., and W. Lotko (2003), Small-scale electric fields in downward auroral current channels, *J. Geophys. Res.*, *108*(A7), 1289.

Toffoletto, F., S. Sazykin, R. Spiro, and R. Wolf (2003), Inner magnetospheric modeling with the rice convection model, *Space Sci. Rev.*, *107*, 175–196.

Trakhtengertz, V. Y., and A. Y. Feldstein (1984), Quiet auroral arcs: ionosphere effect of magnetospheric convection stratification, *Planet. Space Sci.*, *32(2)*, pp. 127–134.

Tsing, N.-K., M. K. Fan, and E. I. Verriest (1994), On analyticity of functions involving eigenvalues, *Linear Algebra and its Applications*, *207*, 159–180.

Tsunoda, R. (2015), Upwelling: a unit of disturbance in equatorial spread f, *Progress in Earth and Planetary Science*, *2*(9), https://doi.org/10.1186/s40,645–015–0038–5.

Tsunoda, R. T., R. C. Livingston, J. P. McClure, and W. B. Hanson (1982), Equatorial plasma bubbles: Vertically elongated wedges from the bottomside *F* layer, *J. Geophys. Res.*, *87*, 9171.

Tu, J., and P. Song (2016), A two-dimensional global simulation study of inductive-dynamic magnetosphere-ionosphere coupling, *J. Geophys. Res., Space*, *121*, 11,861–11,881.

Tu, J., and P. Song (2019), On the momentum transfer from polar to equatorial ionosphere, *J. Geophys. Res., Space*, *124*, 6064–6073.

Tu, J., P. Song, and V. M. Vasyliunas (2014), Inductive-dynamic magnetosphere-ionosphere coupling via MHD waves, *J. Geophys. Res., Space*, *119*, 530–547.

Vasyliunas, V. M. (2012), The physical basis of ionospheric electrodynamics, *Ann. Geophys.*, *30*, 357–369.

Vasyliunas, V. M., and P. Song (2005), Meaning of joule heating, *J. Geophys. Res.*, *110*, A02,301.

Zhu, H., A. Otto, D. Lummerzheim, M. Rees, and B. Lanchester (2001), Ionosphere-magnetosphere simulation of small-scale structure and dynamics, *J. Geophys. Res.*, *106(A2)*, 1795–1806.

Item 2 of Supplementary Information: Supplementary information for Item 1.

Supplementary Information

This file contains the Supplementary Information that is referenced in the preprint, An Electromagnetic Calculation of Ionospheric Conductance that seems to Override the Field Line Integrated Conductivity, by Russell B. Cosgrove (2022).

Contents of this file

1. The Matrix H_{5ES}
2. Parameter Choices
3. Additional Commentary on Energy Conservation
4. Analytic Dependence for Polarization Vectors
5. Validation Figures and Elaboration

S.1 The Matrix H_{5ES}

The matrix H_{5ES} is shown in detail in Figure S.1.

S.2 Parameter Choices

The ionospheric parameters used in the analysis are the same as used by *Cosgrove* [2016] (see their Appendix B), and are reproduced here for reference. An electron density profile derived from Sondrestrom incoherent scatter radar data, with E region arc and substantial F region, is shown in the top left panel of Figure S.2. However, this profile is used only for the green curve of panel l, Figure 2. Otherwise the density is kept constant with altitude, with values as identified in the text. A profile for the ion mass is shown in the top right panel of Figure S.2. A collision frequency profile, including ν_{in}, ν_{en}, and ν_{ei}, is shown in the bottom left panel of Figure S.2. The collision frequency profiles up to 1000 km come from the textbook by *Gurevich* [1978]. However, ν_{ei} is proportional to density, and so the ν_{ei} profile was rescaled to reflect the density profile in Figure S.2, which is slightly different from that assumed by *Gurevich* [1978]. When used with the various constant vertical density profiles in the text, the ν_{ei} profile is rescaled for each. The neutral collisions, ν_{in} and ν_{en}, are set to zero above 1000 km. The formula

$$\nu_{ei} = 54.5 \frac{n_i Z_i^2}{T_e^{3/2}}, \tag{S.1}$$

from *Schunk and Nagy* [2000] is used above 1000 km, where T_e is the electron temperature in Kelvin, n_i is the ion density in cm^{-3}, and Z_i is the ion charge number. We assume $n_i = n$, $Z_i = 1$, and use the electron temperature profile shown in the middle left panel of Figure S.2. Above 1000 km, the T_e profile is motivated by *Abe et al.* [1997]. In the region below 1000 km, the T_e profile is taken from Gurevich's text [*Gurevich*, 1978, nighttime], and is incorporated in ν_{en} and ν_{ei}. The temperature in the region below 1000 km is used only to compare the collision frequency equation (S.1) with the values from the text by *Gurevich* [1978]. The formula (S.1) is found to match exactly the value given by *Gurevich* [1978] at 1000 km, although (S.1) gives negligibly larger values around the F region peak (323 s^{-1} as compared with 300 s^{-1}, at 315 km). Another formula, $\nu_{ei} = \left[34 + 4.18 \ln\left(T_e^3/n\right)\right] n T_e^{-3/2}$, appears in [*Kelley*, 1989], where it is attributed to *Nicolet* [1953]; it gives nearly identical results to equation (S.1).

We have favored the *Gurevich* [1978] results only because that text seems to have a complete treatment that is possible to follow without too much difficulty. *Gurevich* [1978] expands the electron distribution function in terms of Legendre polynomials and solves the Boltzmann equation with the Boltzmann collision integral and the Rutherford scattering cross section. *Schunk and Nagy* [2000] explain that their collision frequency formula is derived from a 13-moment velocity distribution, which arises from an expansion in

$$
H_{5ES} = i \begin{bmatrix}
\nu_{en} + \nu_{ei} - i\vec{k}\cdot\vec{V}_{e0} + \gamma_x V_{e0x} & \Omega_{e0}\hat{B}_z + \gamma_y V_{e0x} & -\Omega_{e0}\hat{B}_y + \gamma_z V_{e0x} & -\nu_{ei}\sqrt{\frac{m_e}{m_i}} & 0 & 0 & i\hat{k}_x\omega_e & 0 & 0 & \frac{m_i}{m_e}\frac{k_B T_n|\vec{k}|}{m_e\omega_e}(-ik_x-\gamma_x) & 0 \\
-\Omega_{e0}\hat{B}_z + \gamma_x V_{e0y} & \nu_{en} + \nu_{ei} - i\vec{k}\cdot\vec{V}_{e0} + \gamma_y V_{e0y} & \Omega_{e0}\hat{B}_x + \gamma_z V_{e0y} & 0 & -\nu_{ei}\sqrt{\frac{m_e}{m_i}} & 0 & i\hat{k}_y\omega_e & 0 & 0 & \frac{m_i}{m_e}\frac{k_B T_n|\vec{k}|}{m_e\omega_e}(-ik_y-\gamma_y) & 0 \\
\Omega_{e0}\hat{B}_y + \gamma_x V_{e0z} & -\Omega_{e0}\hat{B}_x + \gamma_y V_{e0z} & \nu_{en} + \nu_{ei} - i\vec{k}\cdot\vec{V}_{e0} + \gamma_z V_{e0z} & 0 & 0 & -\nu_{ei}\sqrt{\frac{m_e}{m_i}} & i\hat{k}_z\omega_e & 0 & 0 & \frac{m_i}{m_e}\frac{k_B T_n|\vec{k}|}{m_e\omega_e}(-ik_z-\gamma_z) & 0 \\
-\nu_{ie}\sqrt{\frac{m_i}{m_e}} & 0 & 0 & \nu_{in} + \nu_{ie} - i\vec{k}\cdot\vec{V}_{i0} + \gamma_x V_{i0x} & -\Omega_{i0}\hat{B}_z + \gamma_y V_{i0x} & \Omega_{i0}\hat{B}_y + \gamma_z V_{i0x} & -i\hat{k}_x\omega_i & 0 & 0 & 0 & \frac{k_B T_n|\vec{k}|}{m_e\omega_i}(-ik_x-\gamma_x) \\
0 & -\nu_{ie}\sqrt{\frac{m_i}{m_e}} & 0 & \Omega_{i0}\hat{B}_z + \gamma_x V_{i0y} & \nu_{in} + \nu_{ie} - i\vec{k}\cdot\vec{V}_{i0} + \gamma_y V_{i0y} & -\Omega_{i0}\hat{B}_x + \gamma_z V_{i0y} & -i\hat{k}_y\omega_i & 0 & 0 & 0 & \frac{k_B T_n|\vec{k}|}{m_e\omega_i}(-ik_y-\gamma_y) \\
0 & 0 & -\nu_{ie}\sqrt{\frac{m_i}{m_e}} & -\Omega_{i0}\hat{B}_y + \gamma_x V_{i0z} & \Omega_{i0}\hat{B}_x + \gamma_y V_{i0z} & \nu_{in} + \nu_{ie} - i\vec{k}\cdot\vec{V}_{i0} + \gamma_z V_{i0z} & -i\hat{k}_z\omega_i & 0 & 0 & 0 & \frac{k_B T_n|\vec{k}|}{m_e\omega_i}(-ik_z-\gamma_z) \\
i\omega_e\hat{k}_x & i\omega_e\hat{k}_y & i\omega_e\hat{k}_z & -i\omega_i\hat{k}_x & -i\omega_i\hat{k}_y & -i\omega_i\hat{k}_z & 0 & i\frac{m_i}{m_e}\vec{k}\cdot\vec{V}_{e0} & -i\frac{m_i}{m_e}\vec{k}\cdot\vec{V}_{i0} & 0 & 0 \\
\frac{m_e}{m_i}\frac{\omega_e}{|\vec{k}|}(\gamma_x - ik_x) & \frac{m_e}{m_i}\frac{\omega_e}{|\vec{k}|}(\gamma_y - ik_y) & \frac{m_e}{m_i}\frac{\omega_e}{|\vec{k}|}(\gamma_z - ik_z) & 0 & 0 & 0 & 0 & \vec{\gamma}\cdot\vec{V}_{e0} - i\vec{k}\cdot\vec{V}_{e0} + \xi\bar{n}_0 & \xi\bar{n}_0 & 0 & 0 \\
0 & 0 & 0 & \frac{m_e}{m_i}\frac{\omega_i}{|\vec{k}|}(\gamma_x - ik_x) & \frac{m_e}{m_i}\frac{\omega_i}{|\vec{k}|}(\gamma_y - ik_y) & \frac{m_e}{m_i}\frac{\omega_i}{|\vec{k}|}(\gamma_z - ik_z) & 0 & \xi\bar{n}_0 & \vec{\gamma}\cdot\vec{V}_{i0} - i\vec{k}\cdot\vec{V}_{i0} + \xi\bar{n}_0 & 0 & 0 \\
\frac{m_e}{m_i}A_{eix} & \frac{m_e}{m_i}A_{eiy} & \frac{m_e}{m_i}A_{eiz} & \frac{m_e}{m_i}C_{eix} & \frac{m_e}{m_i}C_{eiy} & \frac{m_e}{m_i}C_{eiz} & 0 & D_{ei} & \frac{2m_e\nu_{ei}P_{io}}{(m_e+m_i)k_B T_n\bar{n}_0} & L_{ei} & -2\frac{m_e\nu_{ei}}{m_e+m_i} \\
\frac{m_e}{m_i}C_{iex} & \frac{m_e}{m_i}C_{iey} & \frac{m_e}{m_i}C_{iez} & \frac{m_e}{m_i}A_{iex} & \frac{m_e}{m_i}A_{iey} & \frac{m_e}{m_i}A_{iez} & 0 & \frac{2m_i\nu_{ie}P_{eo}}{(m_e+m_i)k_B T_n\bar{n}_0} & D_{ie} & -2\frac{m_i\nu_{ie}}{m_e+m_i} & L_{ie}
\end{bmatrix}, \text{ where}
$$

$$
\begin{aligned}
A_{\alpha\beta} &= \frac{\vec{\gamma}}{|\vec{k}|}\frac{P_{\alpha 0}\omega_\alpha}{k_B T_n\bar{n}_0} - \frac{5}{3}\frac{i\vec{k}}{|\vec{k}|}\frac{P_{\alpha 0}\omega_\alpha}{k_B T_n\bar{n}_0} - \frac{4}{3}\frac{(\vec{V}_{\alpha 0} - \vec{V}_n)\frac{\omega_\alpha}{|\vec{k}|}}{k_B T_n/m_\alpha}\frac{m_n\nu_{\alpha n}}{m_\alpha + m_n} - \frac{4}{3}\frac{(\vec{V}_{\alpha 0} - \vec{V}_{\beta 0})\frac{\omega_\alpha}{|\vec{k}|}}{k_B T_n/m_\alpha}\frac{m_\beta\nu_{\alpha\beta}}{m_\alpha + m_\beta}, \\
C_{\alpha\beta} &= \frac{4}{3}\frac{(\vec{V}_{\alpha 0} - \vec{V}_{\beta 0})\frac{\omega_\beta}{|\vec{k}|}}{k_B T_n/m_\alpha}\frac{m_\beta\nu_{\alpha\beta}}{m_\alpha + m_\beta}, \\
D_{\alpha\beta} &= -2\frac{m_\alpha\nu_{\alpha n}}{m_\alpha + m_n} - 2\frac{m_\alpha\nu_{\alpha\beta}P_{\beta 0}}{(m_\alpha + m_\beta)k_B T_n\bar{n}_0} - \frac{2}{3}\frac{m_n\nu_{\alpha n}}{m_\alpha + m_n}\frac{\left|\vec{V}_{\alpha 0} - \vec{V}_n\right|^2}{k_B T_n/m_\alpha} - \frac{2}{3}\frac{m_\beta\nu_{\alpha\beta}}{m_\alpha + m_\beta}\frac{\left|\vec{V}_{\alpha 0} - \vec{V}_{\beta 0}\right|^2}{k_B T_n/m_\alpha}, \\
L_{\alpha\beta} &= -i\vec{k}\cdot\vec{V}_{\alpha 0} + \frac{5}{3}\left(\vec{\gamma}\cdot\vec{V}_{\alpha 0}\right) + 2\frac{m_\alpha\nu_{\alpha n}}{m_\alpha + m_n} + 2\frac{m_\alpha\nu_{\alpha\beta}}{m_\alpha + m_\beta}. \\
\hat{k} &= \frac{\vec{k}}{\left|\vec{k}\right|}
\end{aligned}
$$

Figure S.1: Matrix H_{5ES} containing the linearized and Fourier transformed electrostatic five-moment fluid equations. In this work $\vec{V}_{e0}$ and $\vec{V}_{i0}$ are set to zero, and P_{e0} and P_{i0} are set to $k_B T_n \bar{n}_0$.

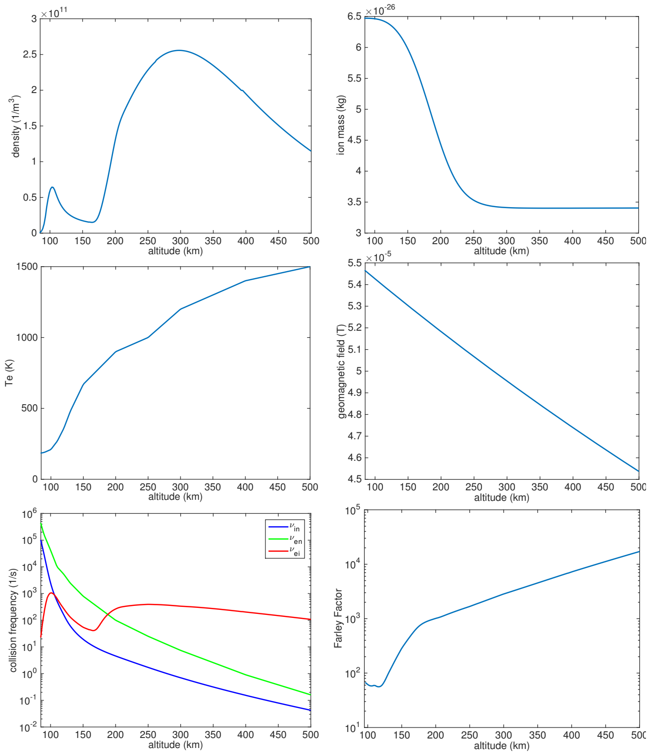

Figure S.2: Ion mass, electron temperature, geomagnetic field, and collision frequencies used in all calculations, along with the associated Farley factor. A density profile is also shown, but is used only for the green curve of panel l, Figure 2. Otherwise the density is kept constant with altitude, with values as identified in the text. This figure is reproduced from *Cosgrove* [2016].

Hermite polynomials about a Gaussian. They reference Chapman and Cowling [1970] for the Chapman-Cowling collision integral, derived for a general inverse power force law. We have compared the results from these two methods and find that they are essentially identical, for our example.

S.3 Additional Commentary on Energy Conservation

Because these are plasma waves, with degrees of freedom and modes of energy storage beyond the electromagnetic, the landscape for energy conservation is more complex than for the usual TL theory.[1] Consider a source with an internal admittance that is positive-real, attached to a TL section with characteristic admittance Y_0. For the usual case where Y_0 is positive-real, it is easy to understand that when there is no back reflected wave in the TL section, the system absorbs energy by carrying it away, and the input admittance ($Y_{in\mathrm{Matched}}$) may be matched to the source. And when there is a back reflected wave the energy is returned, which is consistent with the fact that the input admittance ($Y_{in\mathrm{OC}}$) becomes imaginary, and so is no longer matched to the source.

However, the situation is somewhat different for the ionosphere. For the ionosphere Y_0 is imaginary, and so if there is no back-reflected wave in the TL section the input admittance ($Y_{in\mathrm{Matched}}$) is imaginary, and so not well matched to the source. The mismatch means that the energy is returned, but in this case it is not returned from within the TL section. Rather, the energy never makes it into the TL section at all, that is, the wave amplitudes in the TL section are very small.

On the other hand, when Y_0 is imaginary and there *is* a back-reflected wave in the TL section, the input admittance ($Y_{in\mathrm{OC}}$) is real and so may be matched to the source. In this case the energy may be absorbed even though there is a back-reflected wave within the TL section. How is this possible without wave dissipation, which is not needed for the admittance calculation? In this case the amplitude of the reflected wave may be reduced by nonlinear interaction with the incident wave, with associated heating. Since the heating terms decrease with the square of the wave amplitudes, the fractional reduction of the reflected wave decreases with the wave amplitudes, even as energy conservation is maintained.

And finally we have the case where Y_0 is imaginary and there is a back-reflected wave in the TL section, but the input admittance ($Y_{in\mathrm{OC}}$) becomes negative-real, instead of positive-real. In this case the source and TL section are severely mismatched, and so the net Poynting flux may actually be into the source. Where is this energy coming from? In this case we must appeal to energy conversion. From the analysis surrounding Table 3 in *Cosgrove* [2016], it appears that kinetic energy may be converted into electromagnetic energy [also see *Vasyliunas and Song*, 2005], and so this may supply the energy that is needed to support a negative ionospheric conductance. If so, this is a feature that distinguishes collisional plasma waves, where the mass of the charge carriers may be significant.

S.4 Analytic Dependence for Polarization Vectors

Here we provide details on the method for approximating the eigenvector k_z-dependence by an analytic function. The eigenvectors, $\vec{h}_j$, which make up the columns of the matrix U, are, by convention, constrained to have a complex modulus of one. Hence, variations of the eigenvectors occur through transformations under the 16-dimensional special unitary group, $SU(16)$. It is well known that special unitary transformations can be parameterized about the identity by $\vec{\theta} \in \mathbb{R}^{16^2-1}$ (in the 16-dimensional case), where the transformation is realized by multiplication by the matrix $e^{-i\vec{\theta}\cdot\mathbf{J}}$, and $\mathbf{J}$ is a vector of matrices, which are the $16^2 - 1$ generators of $SU(16)$ [e.g., *Mathews and Walker*, 1970; *Tung*, 1985]. Although we cannot be sure that the dependence of θ on k_z is analytic, we can obtain an analytic function that approximates the eigenvector transformation over the contributing part of the peaked integrand by linearizing the dependence of $\vec{\theta}$ on k_z,

[1] In this work we consider the case where the neutral wind is zero, and so the ionosphere is not a source of energy. Wave amplitudes decrease with time. But when the wind is nonzero it is possible for the ionosphere to be a source of energy, which would manifest as wave amplitudes that increase with time, that is, unstable waves. In this case there is likely no steady state for the system, and so the steady state treatment is not applicable, and neither is the concept of admittance (or conductance). It is necessary to use a time-domain description, and to limit the time horizon such that wave amplitudes remain small, or otherwise to include their non-linear interactions.

that is, $\vec{\theta} \cong (k_z - k_{0z}) \left.\frac{\partial \vec{\theta}}{\partial k_z}\right|_{k_z=k_{0z}}$. Therefore, we will approximate the eigenvector evolution operator by,

$$\Theta(k_z - k_{0z}) = \mathrm{e}^{-i(k_z - k_{0z}) \left.\frac{\partial \vec{\theta}}{\partial k_z}\right|_{k_z=k_{0z}} \cdot \mathbf{J}}. \tag{S.2}$$

The only problem is that it is not completely clear if this exponential function will allow the boundary terms associated with the residue theorem to vanish. However, using again our assumption that the main contribution to the integral can be localized around $k_z = k_{0z}$, there is no harm in multiplying the integrand by an analytic function such as $\mathrm{e}^{\pm i(k_z^3 - k_{0z}^3)/\sigma^3}$, and choosing σ to be sufficiently large that the integral is not affected. Choosing the $\pm$ sign to give exponential decay, the k_z^3 dependence will dwarf any exponential growth that might possibly come from (S.2), regardless of the choice for σ. Thus, we can assume σ is sufficiently large that the integral is not affected, apply the residue theorem, and the residue will not be affected either.

Note that introduction of the form (S.2) is really a formality, so that we can apply the residue theorem and then later argue that $\Theta \cong I$, based on numerical evaluation of the integrals. Hence we need never actually specify the derivatives $\left.\frac{\partial \vec{\theta}}{\partial k_z}\right|_{k_z=k_{0z}}$.

S.5 Validation Figures and Elaboration

Figures S.3-S.5 show comparisons of the exact (i.e., obtained by numerical integration) and eigenmode results at the four corners of the modeling domain, which domain is described in Section 7. While there is some numerical scatter in the integration results, they nevertheless provide a pretty strong indication of convergence to the simple wave-packet interpretation, without corrections. However, there are some anomalies that we discuss in this supplementary section. At this time it is difficult to tell if these anomalies represent actual predictions, numerical inadequacies such as aliasing or windowing, or the result of a failure of our assumption that the source (10) possesses a far zone (which would be a failure of the validation method, and not necessarily an invalidation).

Results for the wavelength and dissipation scale length are shown in Figure S.3, for both modes. Results are shown separately for E_y, B_x, E_x, and B_z, since these are all integrated separately. For reference, the ratio of the dissipation scale to the wavelength is also shown in the bottom row, as it obtains from the simple wavepacket interpretation. With respect to the wavelength, it is evident that the results agree almost exactly in all cases. The only caveat is that it was impossible to obtain results at the lowest altitude (100 km) for one of the four corners, specifically at the corner with transverse wavelength 1000 km and density 10^{11} m^{-3}. This combination of altitude, transverse wavelength, and density produces the lowest ratio of dissipation scale length to parallel wavelength (bottom right panel of Figure S.3), where in this case we mean the dissipation scale length derived from the group velocity. The dissipation scale length for the Whistler mode is about equal to its wavelength, while for the Alfvén mode it is much less, under these conditions. This situation might be expected to pose a challenge to our method of fitting a damped sinusoidal wave to the output from the numerical integration, since the signal may not look like a wave; it may not extend far enough to display oscillation, and it may not extend past the near zone.

It is interesting, however, that in less severe cases it is still possible to fit the Alfvén mode, even though the dissipation length predicted from the group velocity is quite a bit less than the wavelength. In these cases it is still possible to see the Alfvén mode oscillating like a wave, as though it had a much longer dissipation scale: the fits succeed with a reasonable visual appeal, and the dissipation length for the Alfvén mode comes out much longer than that predicted from the group velocity, as can be seen in the low-altitude region of the three right-most columns in Figure S.3. It is entirely possible that this represents a real prediction, and that we should be using a somewhat longer dissipation scale length at the bottom of the E region (for the Alfvén mode). The group velocity is itself difficult for us to calculate, since it requires a finite difference evaluation of the derivative of ω_{jr} (see jittery trace in the bottom right panel of Figure S.3). The fairly-low frequencies that we are evaluating are associated with k_z very close to the value where ω_{jr} becomes zero (for the Alfvén wave), and ω_{jr} is very sensitive to k_z in this area. Concern about this close proximity was actually the major reason we decided it necessary to perform the numerical integrations, as a rigorous test. However, it is not clear which of the two results is more reliable, because the bandwidth and sampling requirements for the

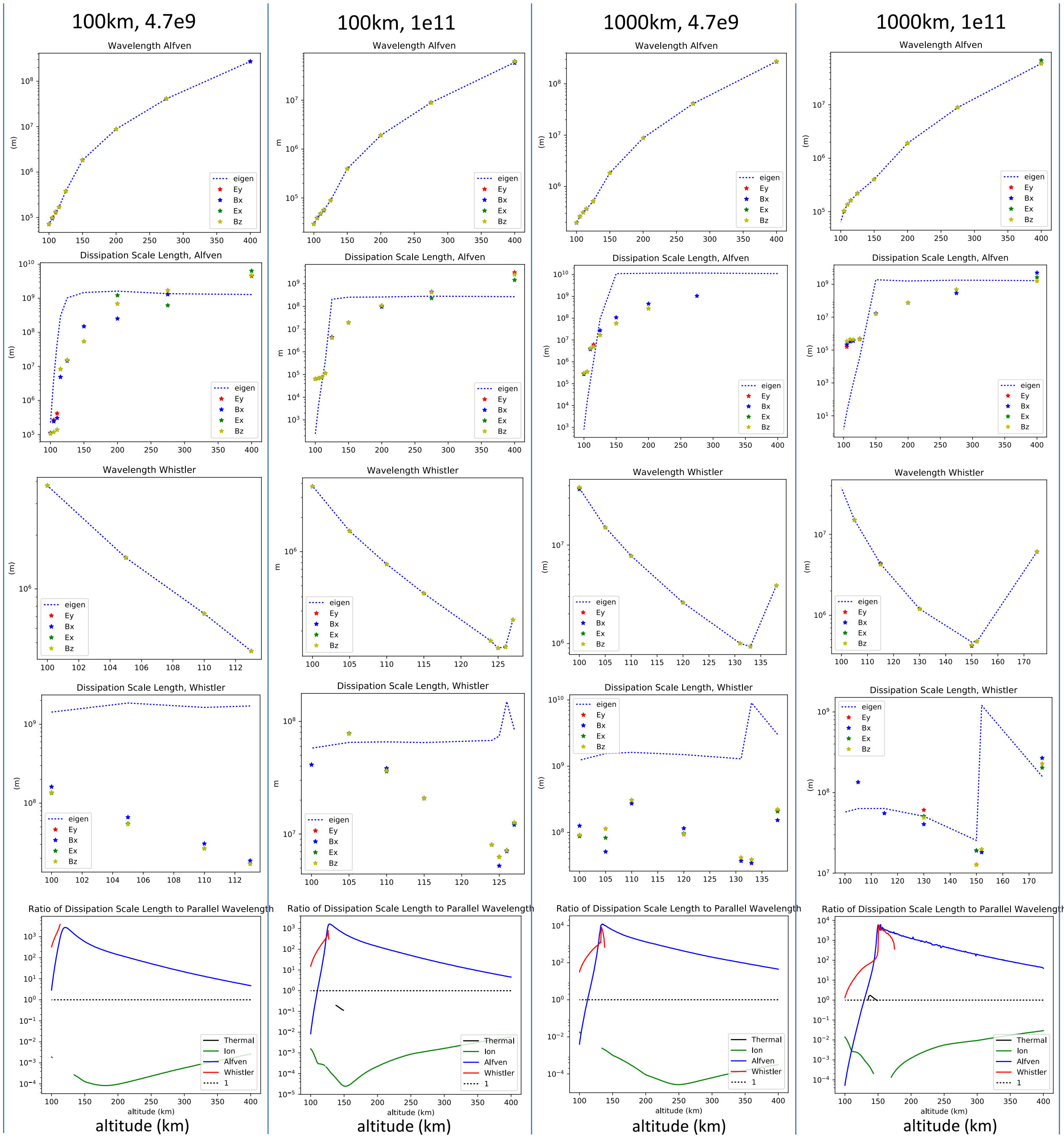

Figure S.3: Validation of parallel wavelength and dissipation scale length. The transverse-wavelength and density are shown at the tops of the columns. For reference, the ratio of dissipation scale length to parallel wavelength under the wavepacket interpretation is shown in the bottom row, for the four modes of Section 6.

bottom of the E region were difficult to realize for the numerical precision (double precision) and number of processors (11) that were available. Also, it may be that we are seeing only a near-zone effect.

Results for the polarization vectors are shown in Figures S.4 and S.5, for the Alfvén and Whistler modes, respectively. Since the polarization vector is arbitrary up to normalization, it is represented by plotting the ratio B_x/B_z, along with the two admittances $B_x/(\mu_0 E_y)$ and $B_x/(\mu_0 E_x)$, where μ_0 is the permeability of free space. Both magnitude and angle are shown for these complex numbers.

For the case of magnitude the agreement is really quite good, with the only exception being, again, the Alfvén mode in the lower E region when the transverse wavelength is 1000 km, and the density 10^{11} m^{-3}. For the case of phase, however, the anomalies in the lower E region extend to the other three cases, and the Whistler wave is also involved. For the most part these anomalies have a scattered appearance, suggesting that they are due either to the numerical limitations mentioned above, or to highly-variable near-zone effects. The regions where this scatter is pronounced generally exhibit evidence of either aliasing (in the case of the Whistler wave), or of a situation where the near zone extends too far from the source, such that the signal does not look completely wavelike (in the case of the Alfvén mode).

Nevertheless, some of the anomalies appear sufficiently consistent as to be worthy of comment. Looking at Figure S.4, in the lower E-region, for the 1000 km wavelength cases, the admittance $B_x/(\mu_0 E_x)$ and the ratio B_x/B_z are negated for the Alfvén wave. This is the area, also noted above, where the dissipation scale length computed from the group velocity is quite a bit less than the wavelength (bottom row of Figure S.3), and also quite a bit less than that calculated by numerical integration. Since the results seem to be consistent in the region below 115 km in altitude it is possible that this represents a real correction to the results obtained from the eigenmodes. However, we do not feel comfortable to implement these corrections until it is possible to verify them with a higher sampling density and wider bandwidth. Also, it may again be that we are seeing a near-zone effect.

Another anomaly concerns the Whistler mode in the altitude regime where, as discussed in the paper, it is probably more analogous to the fast magnetosonic wave than to the usual whistler wave. In this regime the numerical integrations are producing negative phase velocities and admittances that are conjugates of those found from the eigenvectors. These conjugations have been reversed before plotting, since the effect seemed quite clear, and so cannot be seen in the figures. We have tested these possible corrections in the model calculation by applying the conjugations and negating the wavevector over the appropriate altitude regime, and found that the effect is almost unnoticeable. Although we would like to test these results with a higher sampling density and wider bandwidth, it appears that they are not of any consequence.

We have also tested the effect of lengthening the dissipation scale length in the model calculation, since there was an altitude range where the dissipation scale length computed from the group velocity was short enough to have an effect on the Alfvén wave, in the lower E region. In this case we do find a modest effect for the case when the tangent function signature is in evidence, which is the shortest wavelength case. The tangent function signature is somewhat sharpened. The wavelength for the Alfvén wave is very short in the lower E-region, and lengthening the dissipation scale allows this very-short-wavelength mode to participate more in the dynamics. However, the effect at the top of the ionosphere is quite modest, in our testing. And there was no noticeable effect at any altitude for the other three cases, when the tangent function signature was not in evidence.

In the body of the paper we have explained that the anomalies seen in the lower E region may be simply near-zone effects that are particularly associated with the $\delta(z)$ in the source (10), and not relevant to the response of the plasma to radiation-zone waves incident from above. To resolve this matter we would like perform the calculations using a much larger number or processors, and with quadruple precision arithmetic. This would allow us to verify that the computed signal remains unchanged when the sampling density and bandwidth are increased. However, if near-zone effects are actually the culprit then numerical improvements are not going to solve the problem, and it may be necessary to appeal to a time-domain approach for validation and/or correction. But since such a time-domain simulation has not, to our knowledge, been done before, it may be that there are significant obstacles (some of which were mentioned in Section 2). Hence, it may be quite difficult to fully validate (or correct) the eigenvectors and dissipation scale lengths in the lower E region, and it may even be that circuit-theory concepts like conductivity and conductance are not really applicable there, due to a high degree of source-dependency.

However, since the ionospheric conductance is used and will likely continue to be used for quite some time, we have to do something. Therefore, it seems appropriate that for the time being we should content

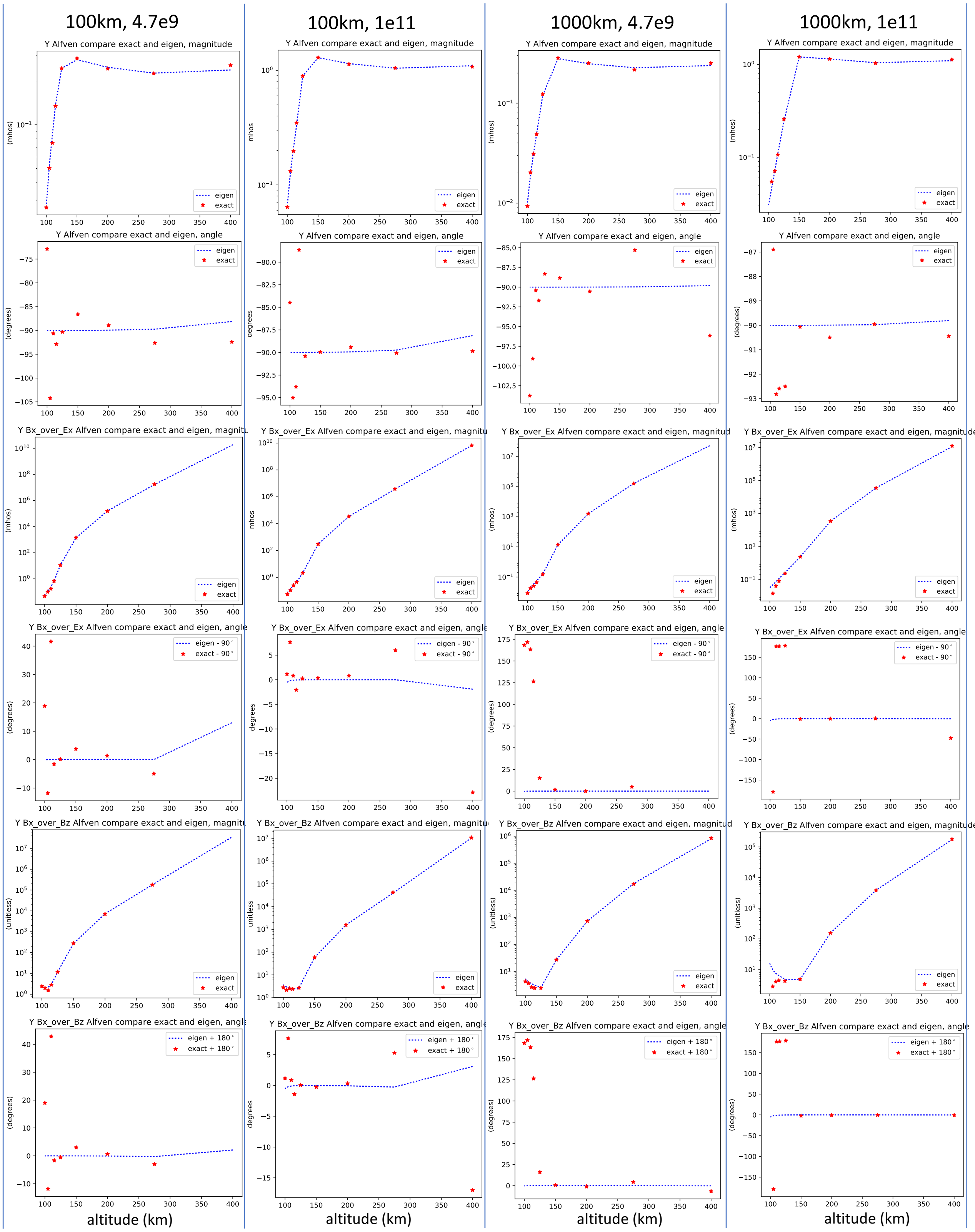

Figure S.4: Validation of the Alfvén wave polarization vectors. The transverse-wavelength and density are shown at the tops of the columns.

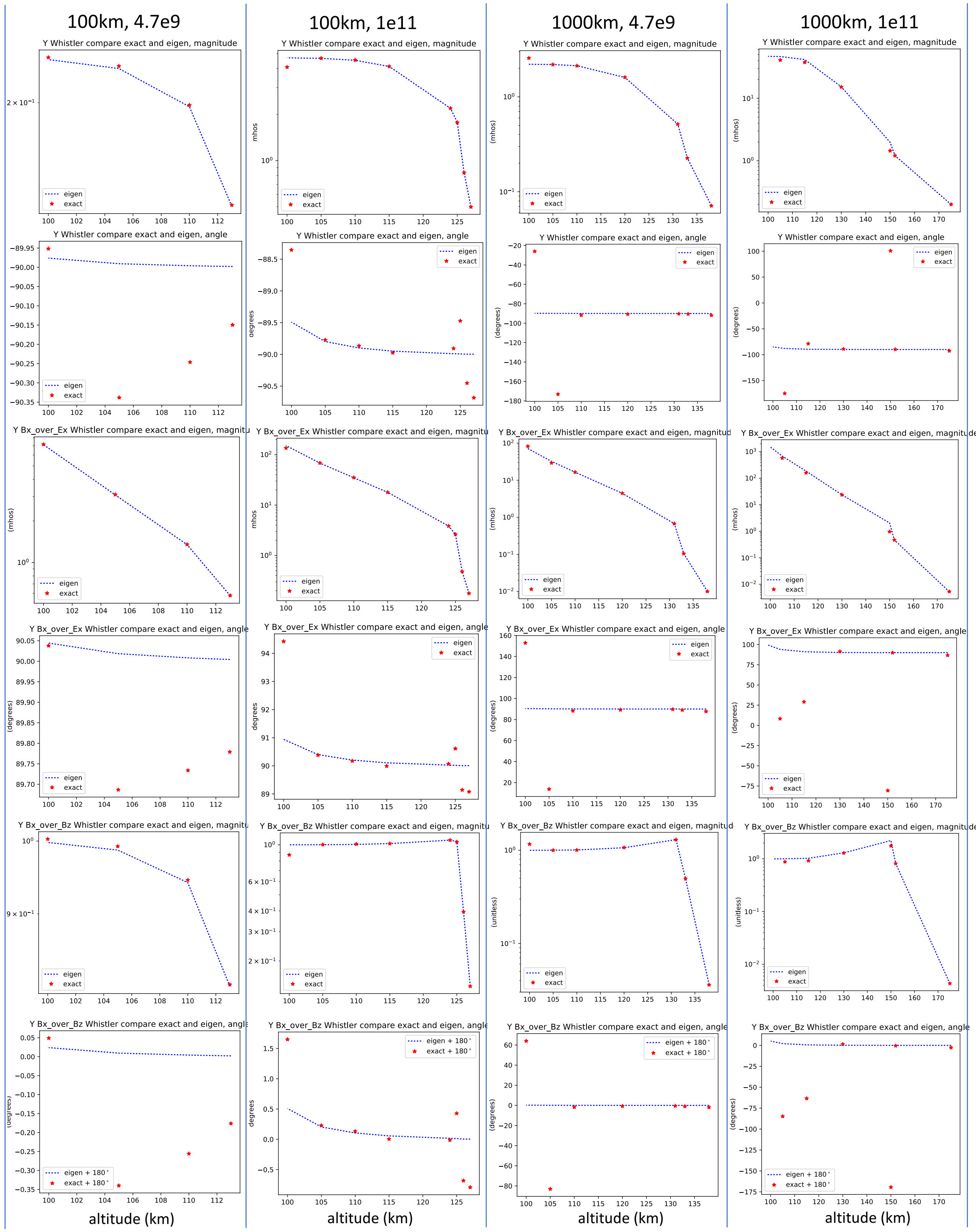

Figure S.5: Validation of the Whistler wave polarization vectors. The transverse-wavelength and density are shown at the tops of the columns.

ourselves with the arguments given in Section 4, that radiation-zone waves traveling into the E region from above will not excite the near-zone effects that the $\delta(z)$ in the source (10) excites, and will instead excite other radiation-zone waves, which may be characterized by the eigenvectors and eigenvalues, and by the usual wave-packet interpretation.

In conclusion, although there are some possible corrections indicated from our attempts at an exact analysis (i.e., the numerical integration of equation (12)), we both do not find the corrections to be sufficiently reliable, and we do not find that the corrections would have a large effect. The altitude regime where the corrections would apply is below the transition region that has been found to block electric field penetration, and this is one likely reason that the effects do not appear very dramatic for a signal entering from above. Another is that the model calculation naturally excludes the Alfvén mode, and favors the Whistler mode, at the bottom of the E region. As long as this exclusion is a correct prediction, the behavior should not be very sensitive to the properties of the Alfvén mode at the bottom of the E region. And one thing seems almost certain, implementing corrections like these is not going to enhance conformance with electrostatic theory in any way. Thus we feel it most appropriate to implement the eigenmode-based model in it's pure form, using the usual wave-packet interpretation. This model is a baseline electromagnetic model that is the most natural extension of the current electrostatic baseline.

References

Abe, T., N. Balan, K. I. Oyama, and G. J. Bailey (1997), Plasmasphere electron temperature–observations and theory, *Adv. Space Res.*, *20(3)*, 401–405.

Chapman, S., and T. G. Cowling (1970), *The Mathematical Theory of Non-Uniform Gasses*, Cambridge University Press, New York.

Cosgrove, R. B. (2016), Does a localized plasma disturbance in the ionosphere evolve to electrostatic equilibrium? evidence to the contrary, *J. Geophys. Res.*, *121*, doi:https://doi.org/10.1002/2015JA021672.

Gurevich, A. V. (1978), *Nonlinear Phenomena in the Ionosphere*, Springer-Verlag, New York.

Kelley, M. C. (1989), *Earth's Ionosphere, Plasma Physics and Electrodynamics*, Academic Press, New York.

Mathews, J., and R. L. Walker (1970), *Mathematical Methods of Physics*, Addison Wesley.

Nicolet, M. (1953), The collision frequency of electrons in the ionosphere, *J. Atmos. Terr. Phys.*, *3*, 200.

Schunk, R. W., and A. F. Nagy (2000), *Ionospheres: Physics, Plasma Physics, and Chemistry*, Cambridge University Press, Cambridge.

Tung, W.-K. (1985), *Group Theory in Physics*, World Scientific.

Vasyliunas, V. M., and P. Song (2005), Meaning of joule heating, *J. Geophys. Res.*, *110*, A02,301.
